# The role of MLO in powdery mildew susceptibility depends on a combination of functional specialization and subcellular localization

**DOI:** 10.1093/plphys/kiag413

**Published:** 2026-06-24

**Authors:** David Bloodgood, Qiong Zhang, Pai Li, Ying Wu, Michael Pan, Christina Zhou, Aspen Hsu, Jun Zhang, Ralph Panstruga, Sharon A Kessler, Ping He, Libo Shan, Cheng-I Wei, Shunyuan Xiao

**Affiliations:** Institute for Bioscience and Biotechnology Research, University of Maryland, Rockville, MD 20850, USA; Institute for Bioscience and Biotechnology Research, University of Maryland, Rockville, MD 20850, USA; Institute for Bioscience and Biotechnology Research, University of Maryland, Rockville, MD 20850, USA; Institute for Bioscience and Biotechnology Research, University of Maryland, Rockville, MD 20850, USA; Institute for Bioscience and Biotechnology Research, University of Maryland, Rockville, MD 20850, USA; Institute for Bioscience and Biotechnology Research, University of Maryland, Rockville, MD 20850, USA; Institute for Bioscience and Biotechnology Research, University of Maryland, Rockville, MD 20850, USA; Institute for Bioscience and Biotechnology Research, University of Maryland, Rockville, MD 20850, USA; Unit of Plant Molecular Cell Biology, Institute for Biology I, RWTH Aachen University, Worringerweg 1, Aachen 52056, Germany; Department of Botany and Plant Pathology, Purdue University, West Lafayette, IN 47907, USA; Department of Molecular, Cellular, and Developmental Biology, University of Michigan, Ann Arbor, MI 48109, USA; Department of Molecular, Cellular, and Developmental Biology, University of Michigan, Ann Arbor, MI 48109, USA; Department of Nutrition and Food Science, University of Maryland, College Park, MD 20742, USA; Institute for Bioscience and Biotechnology Research, University of Maryland, Rockville, MD 20850, USA; Department of Plant Sciences and Landscape Architecture, University of Maryland, College Park, MD 20742, USA

## Abstract

Obligate biotrophic powdery mildew (PM) fungi strictly require living hosts to survive. To search for host factors or processes essential for PM pathogenesis, we conducted a tailored forward genetic screen with the immunocompromised *eds1-2/pad4-1/sid2-2* (*eps*) triple Arabidopsis mutant. This led to the identification of 5 allelic disruptive mutations in *Mildew Locus O 2* (*MLO2*) that are responsible for the compromised immunity yet poor infection (cipi) mutant phenotype upon challenge with an adapted PM isolate. Moreover, the *eds1/pad4/sid2/mlo2/mlo6/mlo12* (*eps3m*) sextuple and the *eds1/pad4/sid2/pen1/pen2/pen3/mlo2/mlo6/mlo12* (*eps3p3m*) nonuple mutants displayed near-complete immunity to adapted and nonadapted PM fungi without signs of defense activation, further strengthening the inference that these 3 clade V MLOs in Arabidopsis may be bona fide host susceptibility factors of PM fungi. Confocal imaging revealed focal accumulation of MLO2-GFP in the peri-penetration peg membranous space, which occurs before and may be required for haustorium differentiation. Ectopic leaf expression analyses of 8 other MLOs belonging to different clades showed that only MLO7 can complement the loss of MLO2, MLO6, and MLO12. Results from domain-swapping analyses between MLO1 and MLO2 suggest a bipartite functional configuration for MLO2: its cytoplasmic C-terminus determines where and when MLO2 functions, while its N-terminal 7 transmembrane domain-containing region executes the cellular function that is critical for PM pathogenesis. Genetic studies further demonstrated that, unlike MLO7 in synergids, focal accumulation of MLO2 does not depend on FERONIA (FER) and its 5 paralogs. Together, these findings define clade V MLOs as host factors co-opted by obligate biotrophic PM fungi for successful host colonization.

## Introduction

Powdery mildew (PM) fungi are obligate biotrophic pathogens that strictly require living host cells to survive and thrive (Schulze-Lefert and Panstruga 2003). Upon landing on a host leaf surface, a PM spore germinates within 6 h, forming an appressorium to penetrate the host cell wall within 7 to 10 h. The sporeling then differentiates the haustorium, a specialized infection structure with presumed functions in nutrient uptake and effector delivery, physically inside the host cell from the tip of the penetration peg in 12 to 14 h (Koh et al. 2005). Concomitant with the development of the haustorium, a host-derived extra-haustorial membrane (EHM) is formed to encase the haustorium (Wang et al. 2009; Berkey et al. 2017). The haustorium is believed to mature in 20 to 24 h, and then it is believed to extract water and nutrients from the host cell to support hyphal growth on the leaf surface. Within 4 to 5 d, the fungus develops an extensive mycelial network, capable of forming reproductive structures (conidiophores) that produce new conidia, thereby completing its asexual life cycle. Like other obligate biotrophic pathogens, PM fungi cannot be cultured and are genetically intractable.

Despite extensive studies into plant–PM fungal interactions, a critical question regarding PM biotrophy remains unresolved: Aside from host-derived nutrients, are there any specific host factors or processes that are truly indispensable for PM pathogenesis? Conventional genetic screens have been conducted to identify mutants that show enhanced resistance to PM fungi. Many such mutants show lesion mimic phenotypes or autoimmunity, with disruptive mutations in likely or proven negative regulators of immunity (Frye and Innes 1998; Tang et al. 2005, 2006; Wang et al. 2008; Zhang et al. 2008). To identify possible host factors or processes that are critical for PM pathogenesis without the complication of autoimmunity, we constructed a triple mutant in which genes encoding 2 essential immune signaling components, EDS1 (Falk et al. 1999) and PAD4 (Jirage et al. 1999), as well as a key enzyme, SID2, required for salicylic acid (SA) biosynthesis (Wildermuth et al. 2001) were mutated. This *eds1-2/pad4-1/sid2-2* (*eps*) triple mutant is super-susceptible to the adapted PM isolate *Golovinomyces cichoracearum* (*Gc*) UCSC1 (Zhang et al. 2018). We then performed a large-scale forward genetic screen using ethyl methanesulfonate (EMS)-mutagenized seeds of *eps* to identify mutants that show compromised immunity yet poor infection (*cipi*), with the goal of identifying potential host susceptibility factors required for PM pathogenesis. Among the 18 *cipi* mutants isolated, 5 mutants with the poorest infection success each contain a causal mutation in *MLO2* (*At1g11310*) (see Results section), suggesting that *mlo2*-conditioned poor infection can be uncoupled from the activation of EDS1-/PAD4-/SA-dependent defense.

The *MILDEW LOCUS O* (*MLO*) was originally discovered to confer broad-spectrum resistance to PM fungal isolates in barley (*Hordeum vulgare*; *Hv*), and recessive mutations in the *HvMLO* gene encoding a 7 transmembrane domain (7TM) protein are responsible for the resistance (Büschges et al. 1997). HvMLO belongs to a 7TM protein family that is restricted to the plant lineage and some algae (Kusch et al. 2016). Similar to barley, the joint impairment of *Arabidopsis thaliana* (*At*) *MLO2*, *MLO6*, and *MLO12* also results in near-complete resistance to adapted PM isolates (Consonni et al. 2006). These earlier findings stimulated targeted mutagenesis of *MLO* genes as a promising avenue for creating PM-resistant crops in recent years (Feechan et al. 2008; Pavan et al. 2011; Zheng et al. 2013; Wang et al. 2014; Kusch and Panstruga 2017; Nekrasov et al. 2017; Acevedo-Garcia et al. 2017b; Wan et al. 2020). Unfortunately, *mlo* mutants with strong or near-complete resistance to PM fungi often display reduced plant stature, elevated levels of SA, increased spontaneous callose deposition, and early leaf senescence, resembling characteristics of autoimmunity (Wolter et al. 1993; Peterhansel et al. 1997; Piffanelli et al. 2002; Consonni et al. 2006; Humphry et al. 2006; Kusch et al. 2019; Freh et al. 2024). Thus, MLOs have been considered to be host susceptibility factors for PM fungi and may act as negative regulators of plant immunity, leading to the inference that *mlo*-mediated resistance is attributable to constitutive or elevated immune responses (Büschges et al. 1997; Acevedo-Garcia et al. 2014). Intriguingly, no genetic components known to function in classical pathogen-associated molecular pattern (PAMP)–triggered immunity (PTI) or effector-triggered immunity (ETI) have been shown to be essential for *mlo*-mediated resistance in Arabidopsis (Consonni et al. 2006; Miklis et al. 2007; Kuhn et al. 2017; Kusch et al. 2019). These observations suggest 2 possibilities: either MLO proteins repress a potent, yet uncharacterized defense pathway, or MLOs are required for a host cellular process critical for PM fungal pathogenesis—such that the observed autoimmunity-like phenotype in *mlo* mutants is a downstream consequence of MLO loss, rather than the primary cause of pathogen resistance.

*MLO* genes in plants belong to a small-to-medium sized gene family with varying members (from a few to a few dozen) that can be divided into 7 clades based on protein sequence similarities (Kusch et al. 2016). The genome of *A. thaliana* contains 15 *MLO* family members, falling into 5 phylogenetic clades. Simultaneous inactivation of the 3 clade V members, *MLO2*, *MLO6*, and *MLO12*, results in near-complete resistance to adapted PM pathogens (Consonni et al. 2006). All *MLO* genes whose functional impairment confers PM resistance in dicots belong to clade V, whereas in monocots, such resistance-associated *MLO*s fall within clade IV (Li and Xiao 2025). The functional equivalence of these 2 clades is suggested by the absence of clade V *MLO*s in monocots (Kusch et al. 2016). Interestingly, mutations in barley *HvMLO* or wheat (*Triticum aestivum*) *TaMLO1*, or *Medicago truncatula MtMLO8*, all of which belong to clade IV, resulted in a reduction in colonization by the arbuscular mycorrhizal fungus *Rhizophagus irregularis* in their respective mutant hosts (Jacott et al. 2020). Notably, clade IV MLOs appear to be restricted to plant species that engage in symbiosis with arbuscular mycorrhizal fungi (Bravo et al. 2016; Jacott et al. 2020). These findings suggest that clade IV/V MLOs serve a conserved and important role in enabling host cell entry of some biotrophic fungi regardless of whether the interaction is mutualistic or pathogenic (Jacott et al. 2021). Several other Arabidopsis *MLO* genes have been shown to be involved in distinct biological processes, including trichome cell wall development (*MLO6*) (Hübbers et al. 2024), root gravitropism and thigmomorphogenesis (*MLO4* and *MLO11*) (Chen et al. 2009; Bidzinski et al. 2014; Zhu et al. 2021), root hair expansion (*MLO15*) (Ogawa et al. 2025), pollen tube growth and guidance (*MLO5*, *MLO9*, *MLO10*, *MLO15*) (Meng et al. 2020; Zhang et al. 2020), and pollen tube reception involving synergid cells (*MLO7*, also known as *NORTIA*, *NTA*) (Kessler et al. 2010; Jones et al. 2017).

All predicted land plant MLO proteins are characterized by an N-terminal extracellular region, a central portion containing 7 transmembrane (7-TM) domains, and a cytosolic C-terminal tail (Kusch et al. 2016). This C-terminal region harbors a calmodulin-binding domain (CaMBD), which interacts with calmodulin (CaM) or CaM-like (CML) proteins (Kim et al. 2002; von Bongartz et al. 2023). Several studies have shown that calmodulin binding and/or calcium signaling are required for the full function of distinct MLOs (Kim et al. 2002; Meng et al. 2020; Zhu et al. 2021; Yuan et al. 2025). Excitingly, several Arabidopsis MLO proteins, including MLO2, have been proposed to possess calcium channel activity when expressed in animal cells (Cui et al. 2022; Gao et al. 2023), suggesting that MLOs may also play conserved roles in calcium-homeostasis and calcium signaling in plant cells. However, how MLOs; presumed calcium channel activity is associated with their distinct biological functions in different cells/tissues or subcellular compartments remains unclear.

A GFP-tagged version of barley MLO (HvMLO-GFP) was shown to accumulate focally at sites of cell wall penetration by the barley PM pathogen *Blumeria graminis* f. sp. *hordei* (*Bgh*), now termed *B. hordei*, in barley leaf epidermal cells (Bhat et al. 2005). This observation led to the speculation that HvMLO is actively recruited by the fungus to modulate vesicle-associated processes at the plant cell periphery, thereby facilitating host entry by *Bgh* (Panstruga 2005). Interestingly, in Arabidopsis, unlike *MLO2*, *MLO6*, and *MLO12*, which are expressed in diverse tissues (Chen et al. 2006; Li and Xiao 2025), MLO7 is prominently expressed in the synergids of the female gametophyte and is relocalized from Golgi bodies to the filiform apparatus of synergid cells upon arrival of the pollen tube (Kessler et al. 2010), which is reminiscent of HvMLO's focal accumulation at the fungal penetration site (Bhat et al. 2005). Moreover, expressing MLO2-GFP from the *MLO7* native promoter largely restored fertility of the *mlo7* mutant, whereas MLO1-GFP failed to do so (Jones et al. 2017). Interestingly, the chimeric protein NTA-MLO1^CTerm^ (also named faNTA), resulting from swapping the C-terminal CaMBD of MLO1 with that of MLO7, was found to be constitutively localized to the filiform apparatus of the synergids, similar to MLO1, and it fully restored fertility of the *mlo7* mutant (Ju et al. 2021). This suggests that the C-terminus of MLO7 specifies its Golgi-to-filiform apparatus trafficking capacity upon pollen tube arrival (Ju et al. 2021), which may be regulated through binding of cognate CaM proteins (Yuan et al. 2025). More recently, MLO6 has been shown to interact and colocalize with EXO70H4, a subunit of the exocyst complex, in trichome cells. Each of the 2 proteins depends on the other for correct subcellular localization, and both are required for callose deposition in the trichome cell wall (Hübbers et al. 2024). These findings suggest that MLO6, and likely its 2 close homologs, MLO2 and MLO12, play an important role in exocytosis of cell wall components in trichomes (Hübbers et al. 2024).

In this study, we show that loss-of-function mutations in *MLO2*, *MLO6*, and *MLO12* result in a near-complete failure of PM pathogenesis, even in *eds1/pad4/sid2* triple and *eds1/pad4/sid2/pen1/pen2/pen3* sextuple mutant backgrounds, both of which are severely compromised in immune signaling and defense against various (potential) pathogens. These observations suggest that the failure of PM infection in *mlo2/mlo6/mlo12* mutants is not immunity-driven. Instead, it likely reflects disruption of a host cellular process essential for PM fungal invasion, which may be co-opted by the pathogen during pathogenesis. Ectopic expression of 8 other Arabidopsis *MLO* homologs from the *MLO2* promoter in leaf tissues of *eds1/pad4/sid2/mlo2/mlo6/mlo12* showed that only MLO7 can significantly complement the loss of MLO2, MLO6, and MLO12. We further demonstrate that MLO2's focal accumulation around the PM penetration peg is determined by its CaMBD-containing C-terminus and that the receptor-like kinase FERONIA (FER), which regulates MLO7's relocalization in synergid cells, is dispensable for MLO2's focal accumulation and function that is co-opted by PM fungi for host entry and colonization.

## Results

### Identification of 5 allelic *MLO2* mutations conferring resistance to PM independent of EDS1, PAD4, and SID2

**Figure 1:**
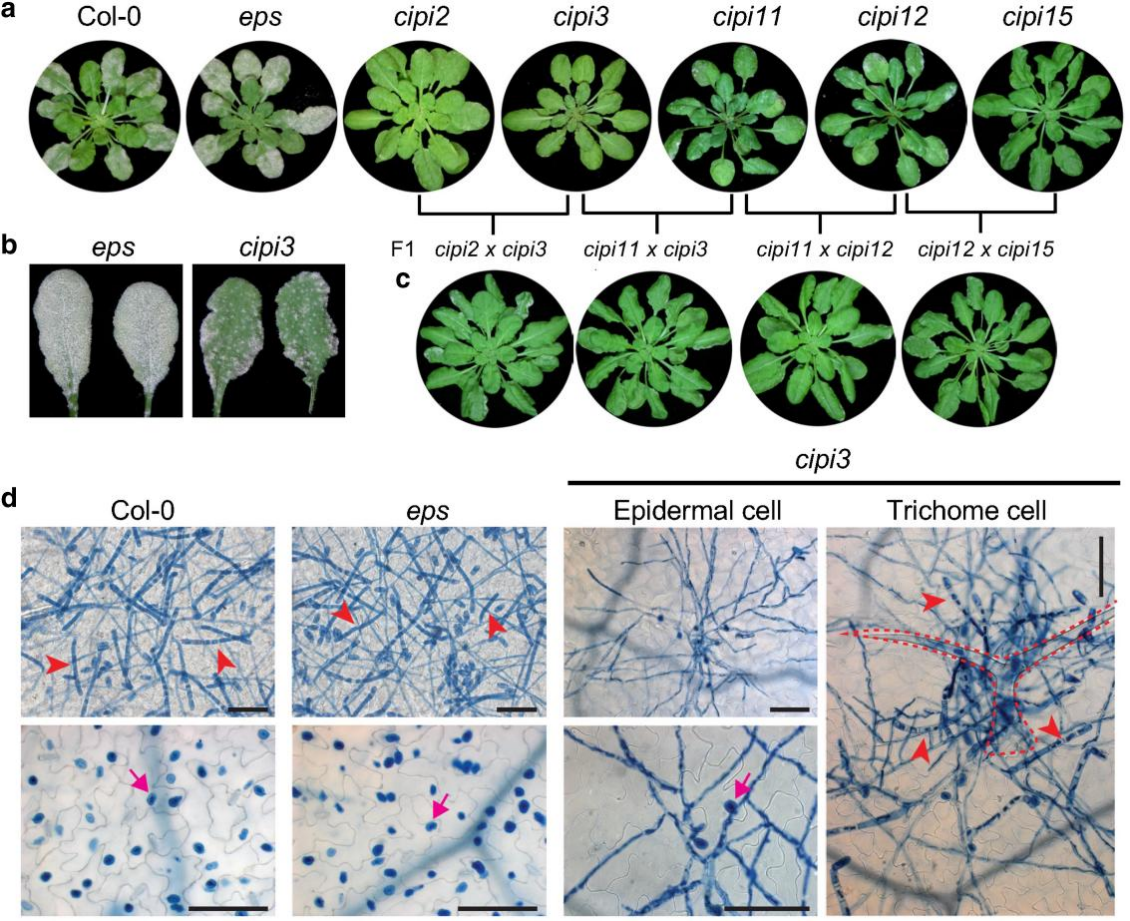
Isolation and phenotypic characterization of 5 allelic *cipi* mutants. Plants were inoculated with the adapted PM isolate *G. cichoracearum* (*Gc*) UCSC1. Photos of infected plants or leaves were taken at 10 to 15 dpi. a) Representative plants of the 5 *cipi* mutants along with Col-0 and the *eps* parental line showing their infection phenotypes at 11 dpi. b) Representative leaves from *eps* and *cipi3* at 13 dpi. Note the trichome-based infection in *cipi3*. c) Infection phenotypes of F1 hybrid plants derived from crosses between the indicated mutants at 12 dpi. d) Microscopic images showing fungal structures stained by trypan blue on the leaf surface (upper panel) or inside the epidermal cells (lower panel) of the indicated genotypes at 11 dpi. Note that the trichome cells support fungal sporulation in the *cipi3* mutant. Arrowheads indicate rod-shaped conidiophores. Arrows indicate haustoria. Dashed lines highlight a trichome cell. Bar=100 μm.

*eds1/pad4/sid2* (*eps*) mutant plants are super-susceptible to the adapted PM isolate *G. cichoracearum (Gc*) UCSC1 (Fig. 1a, Figure S1a) (Zhang et al. 2018). To identify host genes crucial for biotrophic pathogenesis of PM fungi, we designed and conducted a large-scale mutant screen in the background of the *eps* triple mutant. We aimed to isolate higher-order mutants with cipi phenotypes. We reasoned that such *cipi* mutations would more likely disrupt genes essential for PM pathogenesis, independent of immunity activation. Eighteen *cipi* mutants were isolated, of which 5 (*cipi2*, *cipi3*, *cipi11*, *cipi12*, and *cipi15*) showed similarly poor infection success by *Gc* UCSC1 (Fig. 1a; Figure S1a). Spore quantification revealed that sporulation of *Gc* UCSC1 in *cipi3* plants was reduced to only ∼16% of that in *eps* plants, which is only 1/3 of that in Col-0 wild-type plants (Figure S1b). Interestingly, we noticed that rosette leaf trichomes of those 5 *cipi* mutants tend to support more fungal growth and sporulation (Fig. 1b). To determine if the causal mutations in the 5 *cipi* mutants occur in the same gene, we performed crosses between these *cipi* mutants and found that all resulting F_1_ plants showed similarly poor infection success by *Gc* UCSC1, as the respective parental lines (Fig. 1c). This result indicates that the causal mutations in the 5 *cipi* mutants are allelic. Microscopic examination of PM-infected leaves revealed greatly reduced haustorium formation and conidiophore development, except for occasionally infected trichomes in *cipi3* (Fig. 1d) and other allelic *cipi* mutants.

**Figure 2:**
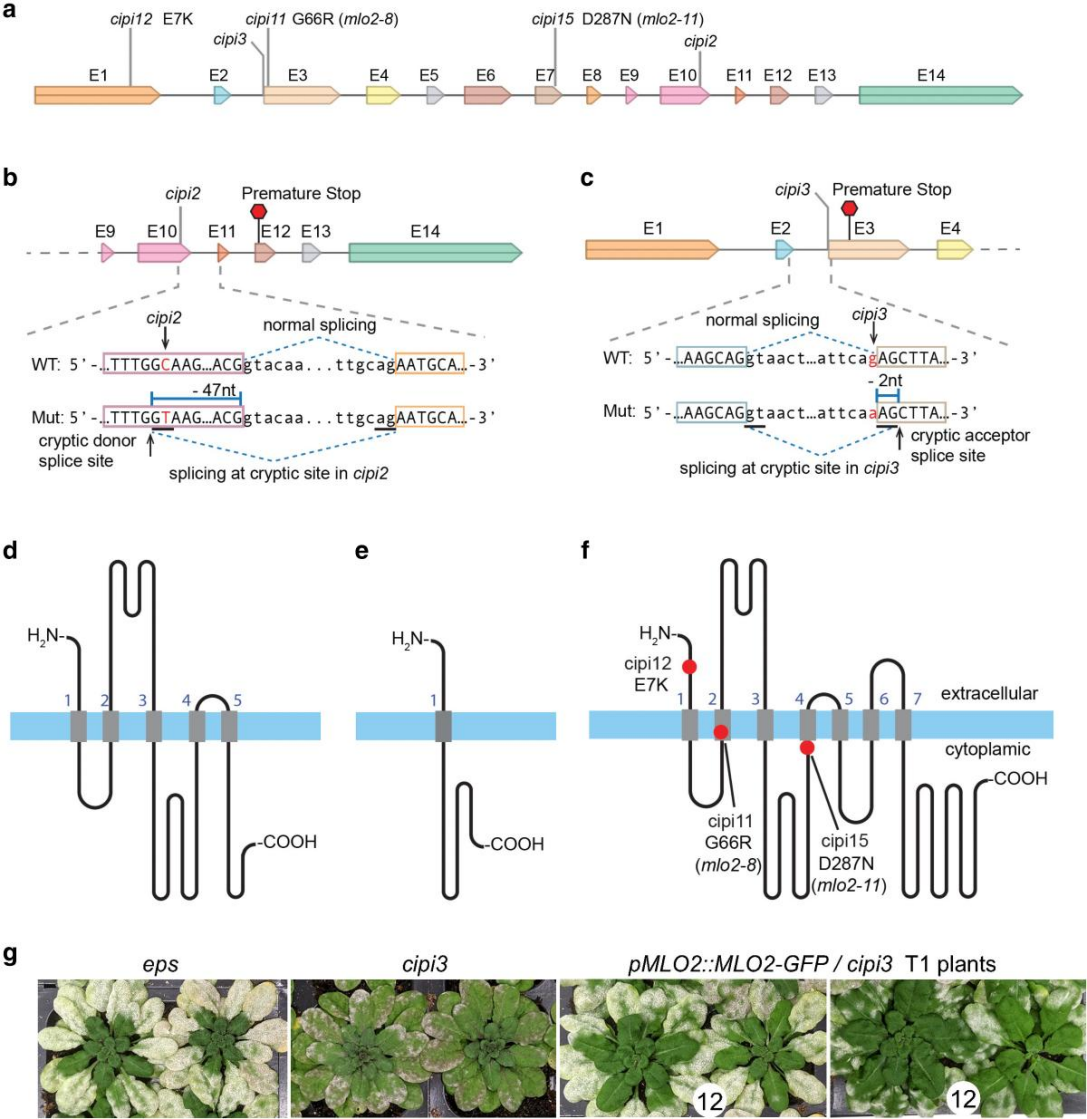
Identification of the 5 allelic causal mutations in *MLO2.* Bulked segregant pool-based whole-genome sequencing identified the candidate causal mutations in *cipi2* and *cipi3* to be in *MLO2*. Targeted sequencing of *MLO2* in *cipi11*, *cipi12*, and *cipi15* revealed disruptive mutations as their respective candidate causal mutations. a) Schematic of the *MLO2* gene structure showing the positions of the nucleotide substitutions in the 5 *cipi* mutants. “E” indicates exon. b and c) Schematic illustration of the positions of the cryptic splice sites (underlined) and the resulting premature stop codons (marked by red hexagons) caused by the mutations in *cipi2* (b) and *cipi3* (c). d to f) Topological illustration of the deduced MLO2 mutant proteins resulting from the *cipi2* (d), *cipi3* (e), and the remaining 3 *cipi* mutations (f). Highlighted in red dots are 3 amino acid substitutions in *cipi11*, *cipi12*, and *cipi15* (f). Blue horizontal bars represent membrane bilayers and gray vertical bars represent transmembrane domains. g) Representative plants from the indicated genotypes infected with *Gc* UCSC1 at 12 dpi. Numbers in white circles indicate the number of T1 transgenic plants showing that infection phenotype.

We then crossed *cipi2* with the *eps* parental line and derived an F_2_ segregating population for genetic mapping of the *cipi2* causal mutation. Whole-genome sequencing of the bulked segregant pool consisting of 65 F_2_ individuals with a *cipi2*-like resistance phenotype revealed a C-to-T synonymous substitution in *MLO2* (*At1g11310*) in the *cipi2* mutant to be co-segregating with the *cipi* phenotype. This mutation is predicted to create an exonic cryptic donor splice site in the 10th exon of *MLO2* (Fig. 2a), which is predicted to result in a 47 nt deletion of the 10th exon starting from the mutation site, generating a premature stop codon in the 12th exon (Fig. 2a and b). Using the same strategy, we identified a G-to-A mutation in the acceptor splice site in the 2nd intron of *MLO2* in *cipi3* (Fig. 2a). This substitution is predicted to activate an immediate downstream exonic cryptic acceptor splice site at the beginning of the 3rd exon, which in turn causes a frameshift resulting in a premature stop codon (Fig. 2c). To investigate if the *MLO2* transcripts in these 2 mutants are indeed mis-spliced, we performed reverse transcriptase-polymerase chain reaction (RT-PCR) analysis using total leaf RNA extracted from *cipi2* and *cipi3* plants and found that the transcripts were mis-spliced (Figure S2). The aberrant transcripts are expected to produce truncated MLO2 proteins of 380 amino acids (*cipi2*) or 97 amino acids (*cipi3*), respectively (Fig. 2d and e), compared to the 573 amino acids of wild-type MLO2. These truncated proteins are probably nonfunctional. Based on these findings, we sequenced the *MLO2* genomic DNA in *cipi11*, *cipi12*, and *cipi15* and found that each of the 3 mutants contains a single nonsynonymous exonic mutation in *MLO2*. As depicted in Fig. 2f, *cipi11* contains a G-to-A mutation that changes a glycine to an arginine (G66R) in the second transmembrane domain, which is identical to the previously reported *mlo2-8* allele (Consonni et al. 2006). *cipi12* contains a G-to-A mutation, resulting in a glutamic acid to lysine exchange (E7K) in the N-terminal extracellular domain, which has not been reported before. Finally, *cipi15* harbors a G-to-A mutation, resulting in an aspartic acid to asparagine substitution (D287N) in the second cytoplasmic loop, which is identical to the *mlo2-11* allele reported before (Consonni et al. 2006).

To provide further genetic evidence that mutations in *MLO2* are responsible for the poor infection phenotype of the 5 *cipi* mutants, we generated transgenic *cipi3* plants expressing *MLO2-GFP* from the native *MLO2* promoter. Half of the 24 T1 *cipi3* plants transgenic for *pMLO2:MLO2-GFP* restored susceptibility to *Gc* UCSC1 to a level close to that of *eps* (Fig. 2g), and the remaining 12 showed medium to low levels of susceptibility, possibly reflecting different levels of *MLO2-GFP* transgene expression (see also below and Fig. 5). These results further demonstrate that the poor PM infection phenotypes in *cipi3* (and likely the other 4 *cipi* mutants) were indeed caused by functional impairment of *MLO2* and that the MLO2-GFP fusion protein is probably fully functional.

Taken together, these genetic data demonstrate that loss of MLO2 significantly reduces PM infection in the *eps* triple mutant background, mirroring the enhanced resistance observed in *mlo2* mutants of Col-0 (Vogel and Somerville 2000; Consonni et al. 2006). These findings further indicate that the reduced PM infection is independent of the combinatory defense programs regulated by EDS1, PAD4, and SID2, which is consistent with previous findings derived from tests with *mlo2/pad4* or *mlo2/sid2* double mutants (Consonni et al. 2006).

### Loss of *MLO2*, *MLO6*, and *MLO12* blocks PM pathogenesis in the *eds1/pad4/sid2* background

**Figure 3:**
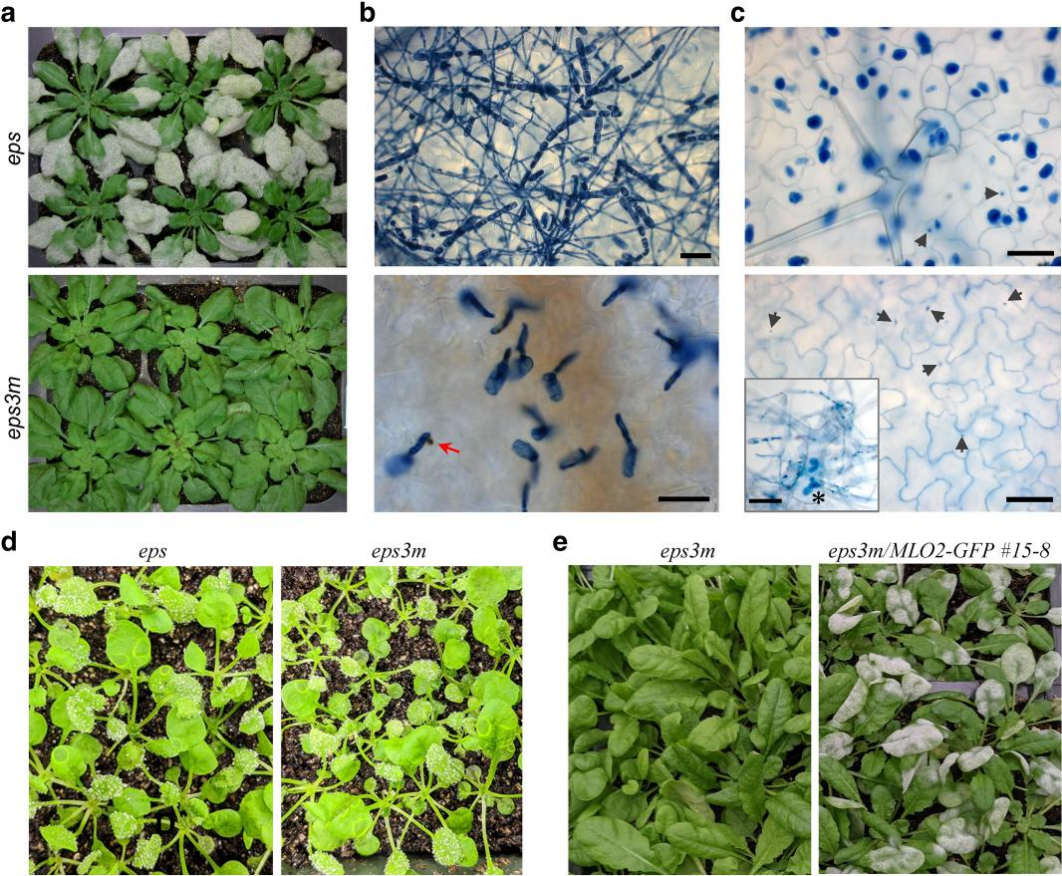
Loss of *MLO2/6/12* in *eps* results in a complete lack of infection by powdery mildew. An *eds1-2/pad4-1/sid2-2 mlo2/mlo6/mlo12* sextuple mutant line (*eps3m-7*) was created using CRISPR/Cas9-targeted mutagenesis. Plants of this line were subjected to infection tests and transformation. a to c) Plants of the indicated genotypes inoculated with *Gc* UCSC1. Plant photos (a) and micrographs with leaf mycelia intact (b) or removed (c) were acquired at 10 and 6 dpi with *Gc* UCSC1, respectively. The fungal structures were stained with trypan blue. The red arrow in b) indicates a penetration peg developed from the appressorium. The arrowheads in c) indicate the attempted penetration sites. Inset in c) shows an infected trichome cell of *eps3m* with the development of haustoria (∗). Bar=50 μm. d) Infection phenotypes of plants of the indicated genotypes inoculated with a virulent oomycete isolate *H. arabidopsidis* Noco2. Photos were taken at 7 dpi. e) *Gc* UCSC1 infection phenotypes of *eps3m* and 1 *eps3m* transgenic line expressing MLO2-GFP from the *MLO2* promoter. Photos were taken at 10 dpi.

**Figure 4:**
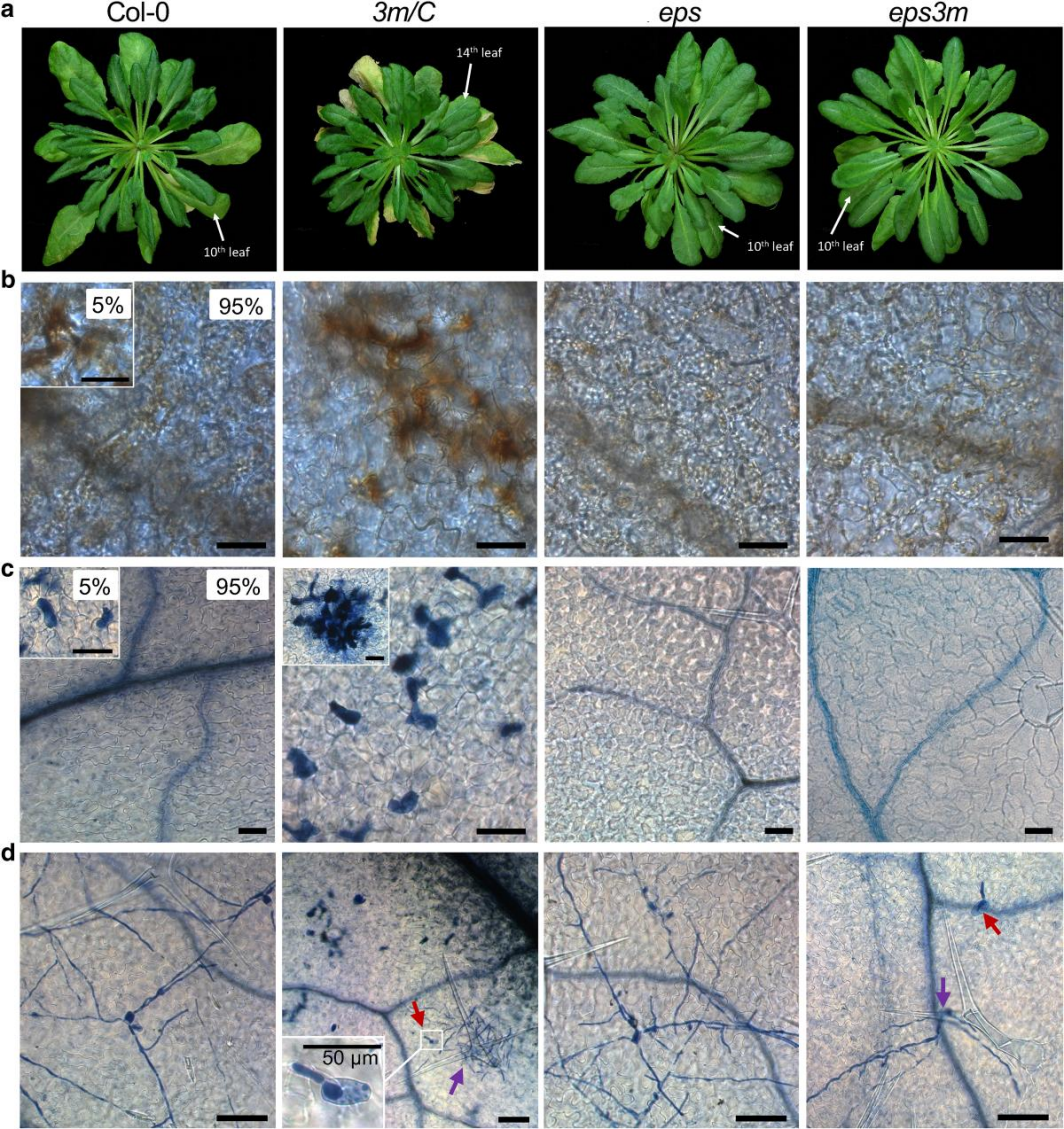
*mlo*-conditioned early leaf senescence is fully suppressed in *eps3m*, and *mlo*-mediated immunity is not associated with cell death. Plants of Col-0, *mlo2-5/mlo6-2/mlo12-1* (*3m/*Col-0), *eps*, and *eps3m* were grown under short-day conditions for 14 wk. Selected leaves were subjected to phenotypical analysis. a) Representative plants of the indicated genotypes showing different degrees of leaf senescence (yellowing). b and c) Representative micrographs showing a leaf section of the 10th leaf of Col-0, *eps* and *eps3m*, and the 14th leaf of *3m/*C after 3,3′-diaminobenzidine staining for in situ detection of H_2_O_2_ (brownish precipitates in b) or trypan blue staining for cell death (dark blue in c). Bar = 50 μm, unless otherwise indicated. d) Representative micrographs showing the *Gc* UCSC1-infected 11th leaves of Col-0, *eps* and *eps3m*, and 15th leaves of *3m/*Col-0 plants at 3 dpi after trypan blue staining. Red arrows indicate germinated sporelings that failed to develop further; purple arrows indicate incidental trichome-supported fungal growth. Bar =100 μm, unless otherwise indicated.

*mlo2-5/mlo6-2/mlo12-1* triple mutant plants in the Col-0 background (designated *3m/C*) exhibit near-complete resistance to PM (Consonni et al. 2006). To see if *3m*-mediated resistance can be recapitulated in the *eps* background, we knocked out *MLO6* and *MLO12* in *cipi3* by CRISPR/Cas9-based mutagenesis to create 3 independent *eds1/pad4/sid2/mlo2/mlo6/mlo12* (*eps3m*) mutants with indel mutations resulting in early stop codons in *MLO6* and *MLO12* (Figure S3). Infection tests with *Gc* UCSC1 showed that plants of these 3 sextuple mutants support no visible fungal growth (Fig. 3a). Microscopic examination revealed invariable arrest of PM sporelings shortly after development of the appressorium (arrow in Fig. 3b) and no haustorium formation in the foliar pavement cells (Fig. 3c). Intriguingly, haustorium formation and limited hyphal development could occasionally be found in rosette leaf trichomes (inset in Fig. 3c), albeit hardly visible to the naked eye, suggesting that the trichome cell physiology is different from that of epidermal pavement cells. Interestingly, the uninfected or infected plants of the *eps3m* mutants did not show early leaf senescence reported for the *3m/C* triple mutant (Consonni et al. 2006), nor did they exhibit any other obvious developmental phenotypes (Fig. 4a), suggesting that the near-complete resistance to *Gc* UCSC1 is unlikely due to constitutive activation of host defense programs in this genotype.

To further evaluate this inference, we used CRISPR/Cas9-based mutagenesis to knock out *MLO2*, *MLO6*, and *MLO12* in the background of the *eds1/pad4/sid2/pen1/pen2/pen3* (*eps3p*) sextuple mutant (Figure S4a). Plants of *eps-pen2* or *eps3p* show super-susceptibility to adapted *Gc* UCSC1 and support the growth of nonadapted dicot PM fungi (Zhang et al. 2018; Wu et al. 2023). As expected, the *eps3p3m* nonuple mutant remained immune to *Gc* UCSC1 and the nonadapted *Gc* UMSG1, which infects sow thistle (Wen et al. 2011) (Figure S4b and c). We also tested *eps* and *eps3m* seedlings with a virulent oomycete pathogen, *Hyaloperonospora arabidopsidis* Noco2. Plants of both genotypes exhibited similar levels of super-susceptibility to this pathogen (Fig. 3d). This further suggests that the failure of PM fungi on *eps3m* plants is not due to the activation of constitutive defenses.

We then created *eps3m* transgenic lines expressing *MLO2-GFP* from the *MLO2* promoter and found that plants of such transgenic lines largely restored susceptibility to *Gc* UCSC1 (Fig. 3e), reinforcing the finding that MLO2 is the major contributor to PM susceptibility among the 3 clade V *MLO* genes (Consonni et al. 2006).

### *Eps* mutations uncouple signs of early leaf senescence in the *3m* mutant from PM resistance

To further ascertain whether the near-complete resistance of *eps3m* to *Gc* UCSC1 can be uncoupled from host defense, we first grew plants of Col-0, *3m/C*, *eps*, and *eps3m* under short-day conditions for 14 wk when old rosette leaves of Col-0 wild-type plants started to show weak leaf yellowing (senescence) (Fig. 4a). At this time, plants of *3m/C* showed massive leaf senescence and reduced stature as anticipated, whereas plants of *eps* and *eps3m* showed no sign of leaf senescence (Fig. 4a). Leaf senescence initiation is known to be associated with reactive oxygen species (ROS) production and accumulation in leaf mesophyll cells, which eventually leads to the collapse of mesophyll and epidermal cells, resulting in leaf yellowing, a typical feature of senescence (Jing et al. 2008; Mayta et al. 2019). To explore if there is any ROS accumulation in leaves of *eps3m* plants, we subjected the 10th leaf from plants of Col-0, *eps*, and *eps3m*, and the 14th leaf of *3m/C* plants to 3,3′-diaminobenzidine (DAB) staining for the visualization of in situ H_2_O_2_ accumulation. While the 10th leaf of the plants of Col-0, *eps*, and *eps3m* showed no or little (in the case of Col-0) visible yellowing, the 10th to the 13th leaves of *3m/C* plants exhibited obvious yellowing, and their 14th leaf showed little yellowing. Subsequent microscopic analysis revealed frequent H_2_O_2_-positive mesophyll cells, detected by brownish precipitates, occurring either individually or in clusters in the 14th leaves of all 6 3*m/C* plants analyzed (Fig. 4b), indicative of the onset of senescence in these leaves. By contrast, such H_2_O_2_-positive cells were completely absent from the 10th leaves of *eps* and *eps3m* plants and were only rarely (∼5% leaf areas) observed in the 10th leaves of Col-0 plants (Fig. 4b), indicating (largely) absence of senescence. We then used trypan blue staining to visualize dead or dying cells in the 10th or 14th leaves of these 4 genotypes and found very similar patterns as observed for H_2_O_2_ accumulation: While cell death was not detected from the 10th leaves of *eps* and *eps3m* plants, and only rarely seen in the 10th leaves of Col-0 plants, collapse of individual and clustered mesophyll cells, and occasionally more than a dozen of both mesophyll and epidermal cells, was frequently found in the 14th leaves of *3m/C* plants (Fig. 4c).

To examine if PM inoculation can trigger cell death in the *3m/C* genotype, and whether such cell death (if any) contributes to the termination of fungal development in *3m/C*, we inoculated the detached 11th or 15th leaves of these 14-wk-old plants with *Gc* UCSC1 and examined the host–fungal interaction by trypan blue staining at 3 d post inoculation (dpi). As shown in Fig. 4d, normal fungal development occurred without cell death in the 11th leaves of the susceptible Col-0 and *eps* plants, whereas the sporelings were completely arrested shortly after germination on pavement cells of both the 15th leaves of *3m/C* and 11th leaves of *eps3m* (indicated by red arrows in Fig. 4d). Notably, while no cell death was observed in *eps3m*, the cell death in *3m/C* was not associated with the early termination of fungal development. As observed before, sporadic hyphal development could be supported by trichome cells in both *3m/C* and *eps3m* (indicated by purple arrows in Fig. 4d).

### Failure of PM infection on *eps3m* plants is not associated with the marked activation of canonical defense markers

We analyzed the expression of canonical defense marker genes in mature rosette leaves of 7-wk-old short-day-grown plants of wild-type (Col-0), *3m/C*, *eps*, and *eps3m* genotypes prior to and at 6, 12, or 48 hpi with *Gc* UCSC1. Before inoculation, none of the plants exhibited signs of early senescence. Four representative marker genes, *FRK1*, *PR1*, *PDF1.2*, and *SAG101*, were chosen: *FRK1* (Flg22-Induced Receptor-Like Kinase 1) is a marker of PTI activation (Asai et al. 2002), *PR1* induction reports the activation of SA-dependent defense responses during ETI (and PTI) and systemic acquired resistance (SAR) (Jing et al. 2008; Tsuda et al. 2013), *PDF1.2* is a marker for the activation of the jasmonic acid (JA) and ethylene (ET) dependent defense pathways (Penninckx et al. 1998), while *SAG101* is a senescence-associated marker gene (He and Gan 2002). As shown in Figure S5a and b, prior to inoculation with *Gc* UCSC1, transcript accumulation of *FRK1* in *3m/C* was higher, though not statistically significant, than that in Col-0, suggesting weak constitutive activation of PTI in *3m/C* plants. At 6 hpi, *FRK1* was induced to higher levels in all 4 genotypes. At 12 hpi, while the expression of *FRK1* reached significantly higher levels in *3m/C*, it subsided almost to basal levels in Col-0, *eps*, and *eps3m*. The expression patterns of *PR1* in the 4 genotypes were similar to those of *FRK1*, except that *PR1* transcript levels were low in all 4 genotypes at 6 hpi. These observations suggest that functional impairment of the 3 *MLO* genes in Col-0 may lead to slightly elevated defenses that are EDS1/PAD4/SID2-dependent, as *eps* and *eps3m* plants showed no or little induction of *FRK1* and *PR1* before or after inoculation. *PDF1.2* transcripts were barely detectable in plants of all the genotypes prior to inoculation and induced to higher levels to varying degrees in each case. However, there were no statistically significant differences among the 4 genotypes at any time points (Figure S5c). Transcript levels of *SAG101* remained stable and were even slightly reduced after PM inoculation, and there were small differences among the 4 genotypes at any time point (Figure S5d), which is consistent with the lack of any visible leaf senescence in these plants during the period of this experiment.

Taken together, the data shown above reinforce the notion that *MLO2*, *MLO6*, and *MLO12* may negatively regulate some *EDS1*/*PAD4*- and SA-associated defense responses. These are nonetheless unlikely to be meaningful in the context of the pronounced *mlo*-based resistance to PM fungi, as both *eps* and *eps3m* plants lack the somewhat higher transcript levels of the marker genes seen in the *3m/C* mutant upon PM challenge (Figure S5).

### MLO2 dosage positively correlates with host susceptibility to *Gc* UCSC1

**Figure 5:**
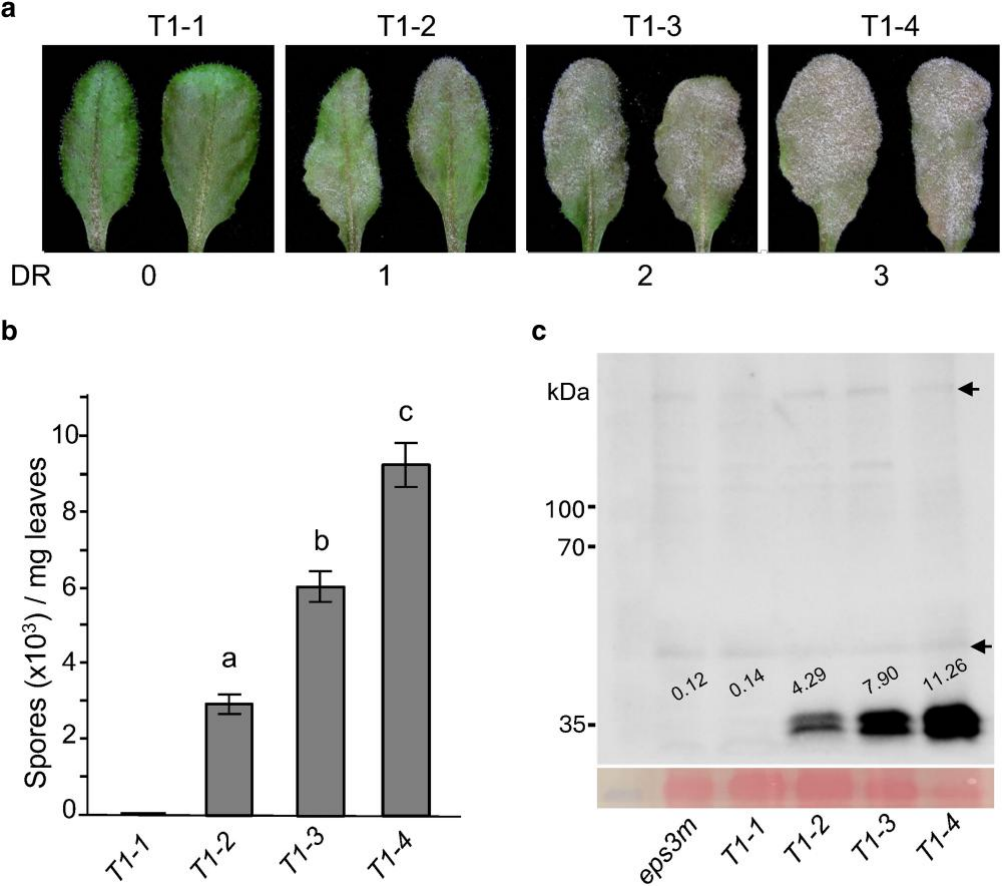
MLO2-GFP expression levels positively correlate with susceptibility to *Gc* UCSC1. a) Representative leaves of 4 selected *eps3m* lines transgenic for *pMLO2::MLO2-GFP* showing different levels of susceptibility to *Gc* UCSC1. Photos were taken at 11 dpi. DR, disease reaction score judged visually based on leaf coverage of fungal mass. b) Quantification of the total number of spores per mg of infected leaf tissues of the 4 lines. Data represent mean±standard deviation of 3 replicates. Different letters indicate statistically significant differences (*P* < 0.05) between the 3 lines, as determined by multiple comparisons using 1-way ANOVA, followed by Tukey's HSD test. This experiment was repeated once with similar results. c) Western blot using anti-GFP antibody to measure MLO2-GFP protein levels in the 4 lines. The *eps3m* parental line served as a negative control. Numbers above the bands indicate the intensity of the 2 bands against the blank background measured with ImageJ. Ponceau S staining at the bottom and nonspecific bands (indicated by arrows) serve as loading control.

Reminiscent of the varied susceptibility observed among *cipi3* mutant plants expressing MLO2-GFP (Fig. 2g), we found that 23 out of 28 total T1 plants of *eps3m* mutants transgenic for *pMLO2::MLO2-GFP* showed varied degrees of infection by *Gc* UCSC1, ranging from minimal, barely visible fungal growth to complete leaf coverage, while the remaining 5 T1 plants lacked any visible fungal infection (Fig. 5a and b). This suggests that MLO2 may facilitate PM pathogenesis in a dosage-dependent manner, depending on transgene expression levels, which was also inferred from the reported differential levels of susceptibility of the transgenic *mlo* genotypes overexpressing an orthologous wheat or rice *MLO* gene (Piffanelli et al. 2004; Elliott et al. 2005; Ge et al. 2020). To determine if the degree of susceptibility in the T1 lines indeed positively correlates with the levels of MLO2-GFP, we performed an immunoblot blot to assess MLO2-GFP levels using an anti-GFP antibody in *Gc* UCSC1-inoculated plants of the 4 independent *eps3m-MLO2-GFP* transgenic lines shown in Fig. 5a and b. The full-length MLO2-GFP (with an expected molecular mass of 93 kDa) was not detectable in this setting; however, 2 bands of lower mass (∼35 kDa) with ascending intensity were detected in the 4 lines with increased levels of susceptibility (Fig. 5b and c). These bands presumably represent C-terminal degradation products of the MLO2-GFP fusion protein. Because the levels of these 2 small bands most likely reflect those of the undetectable full-length MLO2 protein, it is plausible to assume that the MLO2 dosage in those transgenic plants positively correlates with their levels of susceptibility to *Gc* UCSC1. In this context, it is interesting to note that a C-terminal cleavage product was also detected in *E coli-*expressed GST-tagged C-terminus of MLO2, and the cleavage product was diminished when 2 conserved residues of the C-terminus were mutated (von Bongartz et al. 2023). Whether such inferred cleavages have biological relevance to MLO2's functionality remains to be determined.

### ER/Golgi-localized MLO2 is targeted to the membranous space surrounding the penetration peg

**Figure 6:**
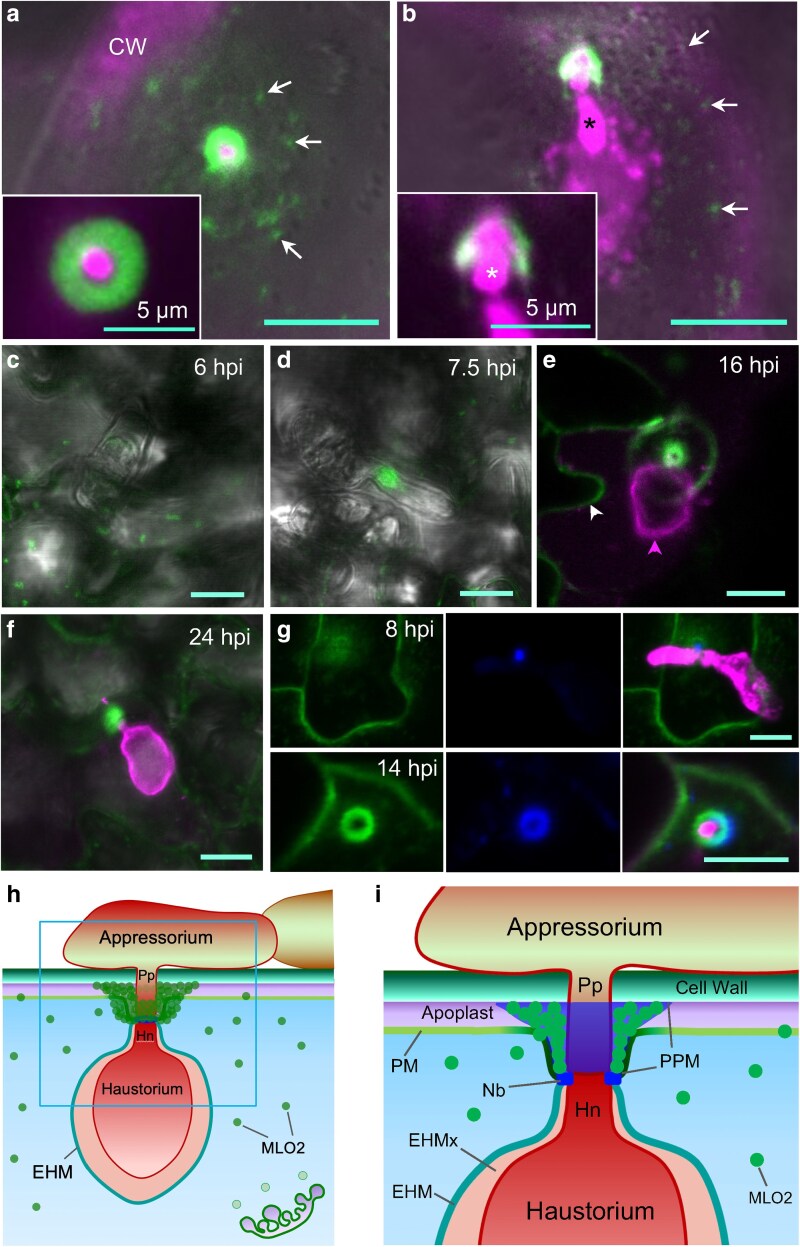
MLO2-GFP is localized to the peri-penetration peg membranous space (PPM). Plants of *eps3m* expressing MLO2-GFP (a and b) or plants of *eps3m* expressing both MLO2-GFP and RPW8.2-RFP (c to f) were used to determine MLO2-GFP's localization by confocal imaging. All images are Z-stack projections of 5 to 15 optical sections. Bar=10 μm, unless otherwise indicated. a and b) Representative images showing localization of MLO2-GFP to the plasma membrane of a leaf epidermal cell penetrated by *Gc* UCSC1. Image in a) is a top-down view of a fungal penetration site, whereas image in b) is a side view of the same site. Insets are a close-up view of a single optical section. The fungal structure is stained with propidium iodide and visualized in magenta. White arrows indicate MLO2-GFP puncta, which may be MVBs or endosomes. A white asterisk indicates the penetration peg, while a dark asterisk indicates the haustorial neck. CW, cell wall. c) A representative image showing punctum distribution of MLO2-GFP at 6 hpi. d) A representative image showing focal accumulation of MLO2-GFP at 7.5 hpi. e) A representative image showing localization of MLO2-GFP and RPW8.2-RFP at 16 hpi. A white arrowhead indicates the plasma membrane, while a magenta arrowhead indicates the extra-haustorial membrane (EHM). f) A representative image showing MLO2-GFP accumulates around the penetration peg, next to the haustorial neck (also see Figure S7). g) MLO2-GFP and callose (shown in blue, stained by sirofluor) largely occupy the same physical space but do not precisely colocalize at the penetration site. h) A cartoon depicting the focal accumulation of MLO2 from Golgi bodies to the penetration site. Pp, penetration peg; Hn, haustorial neck; EHM, extra-haustorial membrane. i) A zoom-in cross-section of h) illustrating the membrane junction where MLO2 focally accumulates. We propose that exosomes derived from MLO2-containing vesicles are secreted into the space between the penetration peg and the host cell wall and plasma membrane to seal this membrane junction. We designate this junction filled with MLO2-containing exosomes or paramural bodies as the peri-penetration peg membranous space (PPM). PPM may also include the perturbed plasma membrane (Pm) and the haustorial neckband (Nb), which is believed to be a diffusion barrier between the apoplast and the extra-haustorial matrix (EHMx).

Barley HvMLO-GFP and Arabidopsis MLO2-GFP have been shown to accumulate at attempted fungal penetration sites (Bhat et al. 2005; Qin et al. 2020). Because the term “penetration site” is rather vague, we sought to determine MLO2's subcellular localization with higher spatiotemporal resolution to better understand the cellular functions of MLO2 in facilitating PM pathogenesis. To this end, we first inoculated plants of the *eps3m*-*MLO2-GFP* transgenic line #4 with *Gc* UCSC1 for refined localization analysis. A close microscopic examination of infected leaves stained with propidium iodide (a dye for fungal structures) at 2 dpi revealed accumulation of MLO2-GFP in a collar-like structure around the penetration peg underneath the appressorium, with MLO2-GFP puncta distributed around the penetration site (Fig. 6a and b). To better examine MLO2-GFP's dynamic spatiotemporal localization during appressorium-haustorium differentiation, we first co-expressed MLO2-GFP with an ER marker (HDEL-mCherry) or a Golgi marker (MAN1 (1-49)-mCherry) (Nelson et al. 2007) in leaves of *Nicotiana benthamiana* via agroinfiltration and found that MLO2-GFP is partially colocalized with these 2 markers (Figure S6). Interestingly, when MLO2-GFP was detected as “grape string”-like aggregations, it exhibited little, if any, colocalization with the ER marker, but good colocalization with the Golgi marker (Figure S6). Next, we introduced the *pRPW8.2::RPW8.2-RFP* construct into the *eps3m*-*MLO2-GFP* background. RPW8.2 is an Arabidopsis PM resistance protein (Xiao et al. 2001) specifically targeted to the extra-haustorial membrane (EHM), encasing the haustorium that emanates from the tip of the penetration peg; hence, RPW8.2 can serve as a reporter of the spatiotemporal biogenesis of the EHM and the haustorium (Wang et al. 2009). A time course analysis showed that PM spores germinated at ∼6 hpi and MLO2-GFP first exhibited detectable focal accumulation at ∼7.5 hpi (Fig. 6c and d), whereas RPW8.2-RFP was first detectable at the EHM around 16 hpi (Fig. 6e), as observed previously (Wang et al. 2009). Importantly, MLO2-GFP was found to accumulate in a membrane compartment (∼1 to 2 μm thick and ∼1 to 3 μm long) surrounding the penetration peg but never in the EHM, which initiates at the haustorial neck connecting to the penetration peg (Fig. 6f; Figure S7). We tentatively named this MLO2-GFP-residing compartment the peri-penetration peg membranous space (PPM). Callose (β-1,3-glucan), synthesized by the callose synthase PMR4, is rapidly deposited at attempted fungal penetration sites and is a major structural component of the papilla defense barrier (Jacobs et al. 2003; Nishimura et al. 2003). To determine whether MLO2 localizes to the same physical space as the developing papilla, we examined callose deposition and MLO2−GFP distribution in leaves of *eps3m–MLO2-GFP* plants following inoculation with *Gc* UCSC1. Callose accumulation was readily detectable by 8 hpi and largely coincides with the focal enrichment of MLO2−GFP (Fig. 6g, upper panel). By 14 hpi, both callose and MLO2−GFP signals intensified at the fungal penetration site and showed substantial, though not complete, colocalization (Fig. 6g, lower panel). These observations imply that MLO2 is specifically recruited to the PPM, where it may play an important role in sealing and stabilizing the convoluted membrane junction (Fig. 6h and i), thereby supporting haustorium differentiation (see Discussion).

### Ectopic expression of *MLO7* but not *MLO1* partially restores susceptibility of *eps3m* to *Gc* UCSC1

**Figure 7:**
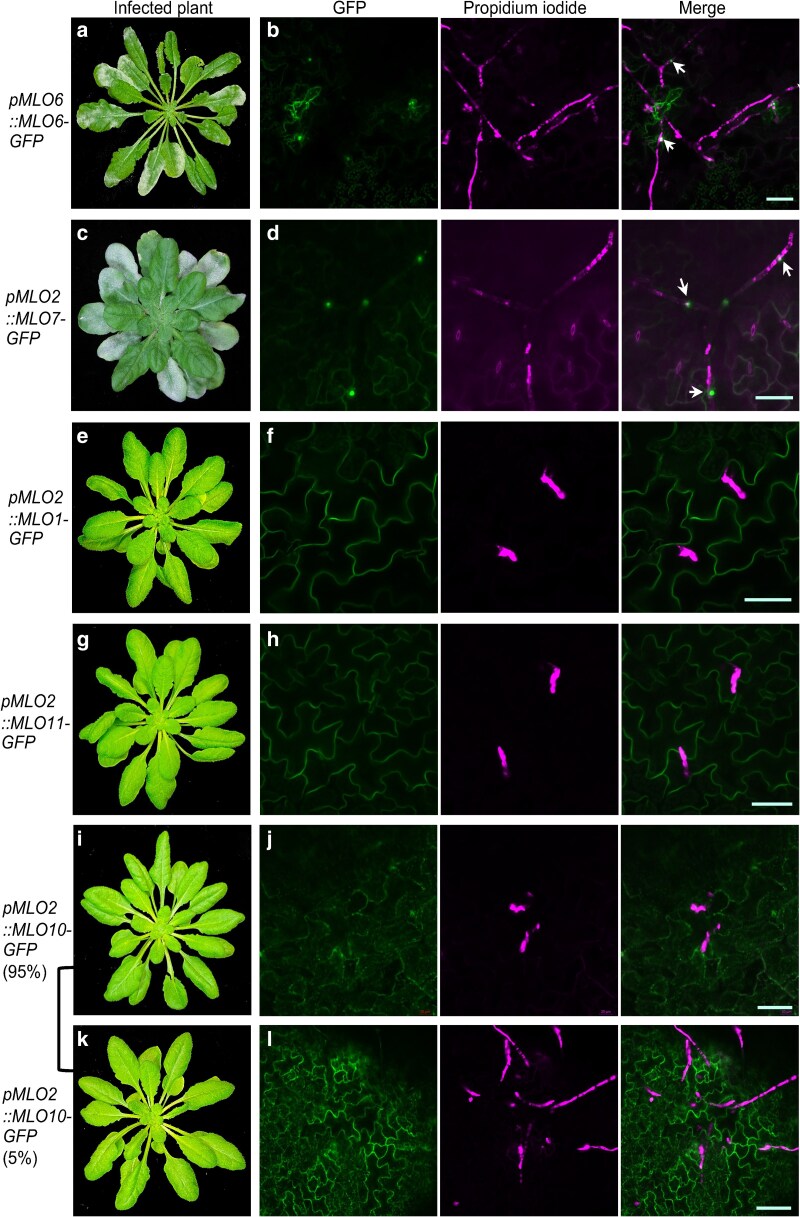
Functional complementation tests with other MLO family members in *eps3m.* Plants of *eps3m* lines transgenic for the indicated *MLOx-GFP* DNA constructs were inoculated with *Gc* UCSC1. Infection phenotypes were visually and microscopically examined. Plant photos were taken at 10 to 11 dpi. Infected leaves were subjected to confocal imaging at 3 dpi to assess the localization of each MLOx-GFP fusion protein. Images shown are Z-stack projections of 3 to 5 optical sections. Fungal structures were stained with propidium iodide and visualized in magenta. White arrows indicate penetration sites. Bar=50 μm, unless otherwise indicated. a, c, e, g, i, and k) A representative plant transgenic for the indicated transgene showing either visible whitish fungal mass (a and c) or lack of it (e, g, i, and k) at 10 to 11 dpi. b, d, f, h, j, and l) Representative confocal images showing expression and localization of the indicated MLOx-GFP fusion protein and fungal development in infected leaf epidermal cells at 3 dpi. Note, a small percentage of MLO10-GFP-expressing plants showed limited hyphal growth (l).

Several *MLO* family members in Arabidopsis exhibit distinct expression patterns and serve distinct biological functions (Chen et al. 2006; Davis et al. 2017; Jones and Kessler 2017; Li and Xiao 2025). Given that MLO1, MLO2, and several other MLOs assayed exhibit calcium channel activity when ectopically expressed in mammalian cells (Gao et al. 2022, 2023), we wondered if MLOs belonging to other clades and expressed in other organs/tissues share the same molecular functions as MLO2 in facilitating PM pathogenesis when ectopically expressed in leaf epidermal cells. Since the *eps* triple mutant is super-susceptible to *Gc* UCSC1, while *eps3m* is essentially immune to *Gc* UCSC1, we reasoned that any functional complementation of the loss of *MLO2/6/12* in *eps3m* should be readily manifested by restored fungal growth visible to the naked eye. To test this, we generated *eps3m* transgenic plants expressing barley *MLO* (*HvMLO*) from the constitutive cauliflower mosaic virus *35S* promoter and found that 9 of 20 T1 transgenic plants supported varied degrees of susceptibility to *Gc* UCSC1 (Figure S8a), indicating that HvMLO can largely perform the same molecular function as MLO2 in Arabidopsis, despite its association with a different clade (clade IV) in the MLO phylogenetic tree (Kusch et al. 2016). We then generated transgenic *eps3m* lines expressing MLO6-GFP from the *MLO6* promoter. Five of 14 *Gc* UCSC1-infected T1 plants transgenic for *MLO6-GFP* showed visible but limited fungal growth (Fig. 7a) with ∼10% of spore production compared to those expressing *MLO2-GFP* from the *MLO2* promoter (Figure S8b and c). Confocal imaging showed that MLO6-GFP also exhibited similar focal accumulation at the fungal penetration site in the 5 transgenic lines (Fig. 7b). These results support the notion that *MLO2* plays a major role while *MLO*6 and *MLO12* play a minor role in permitting PM pathogenesis (Consonni et al. 2006) and that protein accumulation at PPM may be a common feature of MLOs that function as susceptibility factors in PM pathogenesis. The above results also demonstrate that PM infection of *eps3m* transformants can be used as a sensitive reporter to evaluate whether any candidate wild-type or mutant *MLO* gene can perform the same molecular function as *MLO2* by ectopically expressing it in leaf epidermal cells of *eps3m* from the *MLO2* promoter.

A similar strategy was previously employed to demonstrate that expression of *MLO2* in synergid cells from the *MLO7* (*NTA*) promoter significantly restored the fertility of the *mlo7* mutant (Jones et al. 2017), suggesting that MLO2 and MLO7 share a similar molecular function in synergid cells. To provide concrete evidence for the converse scenario, we generated *eps3m* plants expressing MLO7-GFP from the *MLO2* promoter and found that 5 of 18 T1 plants were moderately susceptible and 6 T1 plants were weakly susceptible to *Gc* UCSC1 (Fig. 7c; Figure S8d). Not surprisingly, like MLO2-GFP, MLO7-GFP also exhibited focal accumulation at the PPM (Fig. 7d). This indicates that MLO7 can partially complement the loss of *MLO2/6/12* if ectopically expressed in leaf epidermal cells.

To further expand such functional assays to other MLO clades, *MLO4* and *MLO11* (belonging to clade I), *MLO1* (belonging to clade II), *MLO5* and *MLO10* (belonging to clade III), and *MLO3* (belonging to clade VI) were cloned into the same binary vector that contains the *MLO2* promoter for translational fusion with *GFP* (Figure S9). At least 8 *eps3m* T1 transgenic plants were produced for each construct. All T1 plants were inoculated with *Gc* UCSC1 to determine (i) if any plants can support fungal growth and sporulation and (ii) whether the fusion proteins are detectable by confocal microscopy and, if so, where they are located.

Intriguingly, none of the T1 plants expressing any of the 6 tested *MLO* genes (*MLO1*, *MLO3*, *MLO4*, *MLO5*, *MLO10*, and *MLO11*) supported visible growth of *Gc* UCSC1 (Fig. 7e, g, i, and k), and no GFP signal was reliably detected in the T1 plants transgenic for *MLO3-GFP*, *MLO4-GFP*, and *MLO5-GFP*. Among the 60 *eps3m* T1 plants expressing *MLO1-GFP*, GFP fluorescence was readily detected at the plasma membrane (labeled by the lipophilic dye FM4-64) of leaf epidermal cells (Figure S10a), which is consistent with the MLO1-GFP localization pattern observed in epidermal cells of *N. benthamiana* leaves after agrobacterium-mediated transient expression (Jones and Kessler 2017). Notably, sporelings were arrested probably due to failure in host entry in *eps3m* plants expressing MLO1-GFP, and there was no detectable change of MLO1-GFP subcellular localization in response to attempted fungal penetration (Fig. 7f). Similarly, GFP fluorescence was detected at the plasma membrane of leaf epidermal cells in the 8 *eps3m* T1 plants transgenic for MLO11-GFP (Figure S10b), and the sporelings failed to develop further (Fig. 7h). Interestingly, although no fungal growth was visible to the naked eye in any of the 15 *eps3m* T1 plants transgenic for *MLO10-GFP* (Fig. 7i and k), a few small fungal colonies with very limited hyphal growth and without conidiophore formation were detectable in T2 progenies of 2 independent T1 lines (Fig. 7l). MLO10-GFP was detected in puncta and likely at the plasma membrane, but there was no obvious focal accumulation even in the case of successful penetration reported by limited mycelial growth (Fig. 7l). These observations suggest that, in terms of subcellular localization, MLO10 is similar to MLO2 but distinct from MLO1 and MLO11. Given that MLO10 was able to restore fertility of *mlo7* when expressed in synergid cells (Jones et al. 2017), the very limited ability of MLO10-GFP to support PM susceptibility in the *eps3m* background was unexpected, especially given its close sequence-relatedness to MLO7.

To validate the 3 MLO-GFP fusion constructs, ie *MLO3-GFP*, *MLO4-GFP*, and *MLO5-GFP*, whose expression was not detectable in the respective transgenic plants, and to assess their subcellular localization, we transiently co-expressed each of these 3 constructs with HDEL-mCherry and Man-mCherry (Nelson et al. 2007) in *N. benthamiana* leaves via agroinfiltration. Confocal imaging at 3 d post-infiltration revealed that all 3 fusion proteins were detectable and exhibited similar patterns of partial colocalization with HDEL-mCherry and/or Man1-mCherry (Figures S11 to S13).

These observations suggest that MLO3-GFP, MLO4-GFP, and MLO5-GFP are also likely localized to the ER and/or Golgi compartments in *Arabidopsis*; however, their low expression and/or rapid turnover may prevent their reliable detection in stable *eps3m* transgenic plants.

### Domain-swapping between MLO1 and MLO2 reveals the C-terminal CaM-binding domain as a key determinant of subcellular localization

**Figure 8:**
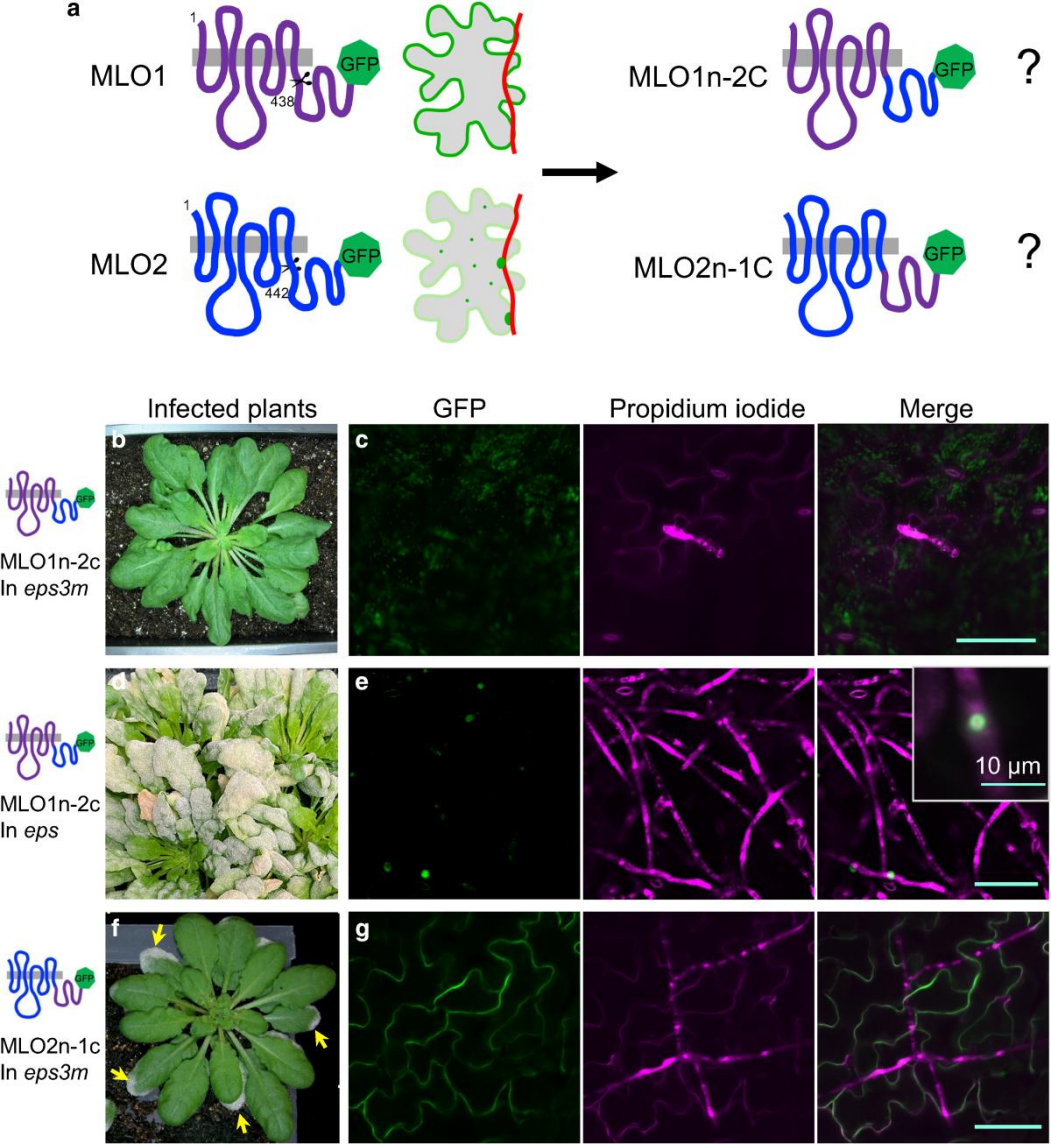
Domain-swapping analysis between MLO1 and MLO2 reveals 2 functional arms of MLO2. a) Schematic illustration of the experimental design. b, d, and f) Infection phenotypes of representative plants expressing MLO1n-2c-GFP or MLO2n-1c-GFP from the *MLO2* promoter in either the *eps3m* background (b and f) or the *eps* background (d). Photos were taken at 12 dpi with *Gc* UCSC1. Arrows in f) point to infected leaf tips with visible fungal mass. c, e, and g) Representative confocal images showing localization of the indicated chimeric fusion proteins in leaf epidermal cells at 3 dpi. Images are Z-stack projections of 3 to 5 optical sections. Fungal structures were stained with propidium iodide and visualized in magenta. Inset in e) is a close-up view of a single penetration site where MLO1n-2c accumulates. Bar=50 μm, unless otherwise indicated.

MLO7 (ie NTA) localizes to Golgi bodies in synergid cells and redistributes to the plasma membrane at the filiform apparatus during pollen tube reception (Jones et al. 2017; Ju et al. 2021). A chimeric MLO protein that contains the N-terminal 7TM portion of NTA and the cytoplasmic CaMBD-containing C-terminus of MLO1 (designated faNTA) exhibits constitutive localization at the filiform apparatus and is able to restore the fertility of *nta* mutant plants (Ju et al. 2021). This finding suggests that the C-termini of these MLO proteins govern their respective localization. To determine whether the CaMBD-containing C-termini of MLO1 and MLO2 dictate their respective subcellular localization in leaf epidermal cells, we constructed 2 chimeric genes by mutually swapping the fragments encoding their C-terminal domains. The first resulting chimeric protein consists of MLO1's N-terminal 7TM section (amino acids 1 to 438) and MLO2's C-terminus (amino acids 442 to 574) (designated MLO1n-2c), while the second comprises MLO2's N-terminal 7TM section (amino acids 1 to 442) with MLO1's C-terminus (amino acids 439 to 526) (designated MLO2n-1c) (Fig. 8a). These chimeric genes, *MLO1n-2c* and *MLO2n-1c*, were translationally fused with GFP at their C-termini, and the resulting fusion constructs were stably expressed from the *MLO2* promoter in *eps3m* plants. More than 20 *eps3m* T1 plants transgenic for either of the 2 chimeric fusion genes were generated and inoculated with *Gc* UCSC1. None of the 23 T1 plants transgenic for *MLO1n-2c-GFP* supported any visible fungal growth, and propidium iodide staining showed that sporelings were arrested shortly after germination (Fig. 8b). The chimeric MLO1n-2c-GFP protein exhibited punctate distribution (Fig. 8c). To test if successful penetration followed by haustorial differentiation can induce MLO1n-2c-GFP's focal accumulation, we introduced the same *pMLO2::MLO1n-2c-GFP* construct into the *eps* background to allow host cell entry and growth of *Gc* UCSC1. The T1 transgenic plants were as susceptible as *eps* plants (Fig. 8d), indicating that MLO1n-2c expression has no dominant negative effect. Interestingly, MLO1n-2c-GFP was found to accumulate at the fungal penetration site in the *eps* transgenic plants (Fig. 8e). In contrast to MLO1n-2c, 7 out of 21 e*ps3m* T1 plants transgenic for *MLO2n-1c-GFP* supported fungal hyphal growth as revealed by propidium iodide staining at 3 dpi (Fig. 8g), and 3 of them also supported weak *Gc* UCSC1 infection visible to the naked eye at 13 dpi (Fig. 8f). Interestingly, similar to MLO1-GFP (Fig. 7f), MLO2n-1c-GFP exhibited plasma membrane localization in leaf epidermal cells, and there was no obvious focal accumulation around the fungal penetration site (Fig. 8g).

Collectively, the above results indicate that the C-terminus of MLO1 confers plasma membrane localization, whereas the C-terminus of MLO2 endows distribution in puncta and fungal penetration-induced focal accumulation at the PPM. The results also suggest that (i) the N-terminal 7TM portion of MLO2, but not that of MLO1, specifies the function of MLO2 in accommodating entry of the PM pathogen and (ii) the full function of MLO2 requires its enrichment at the PPM, which is governed by its CaMBD-containing C-terminus.

### Loss of *FERONIA* does not affect MLO2's localization and role in facilitating PM pathogenesis

**Figure 9:**
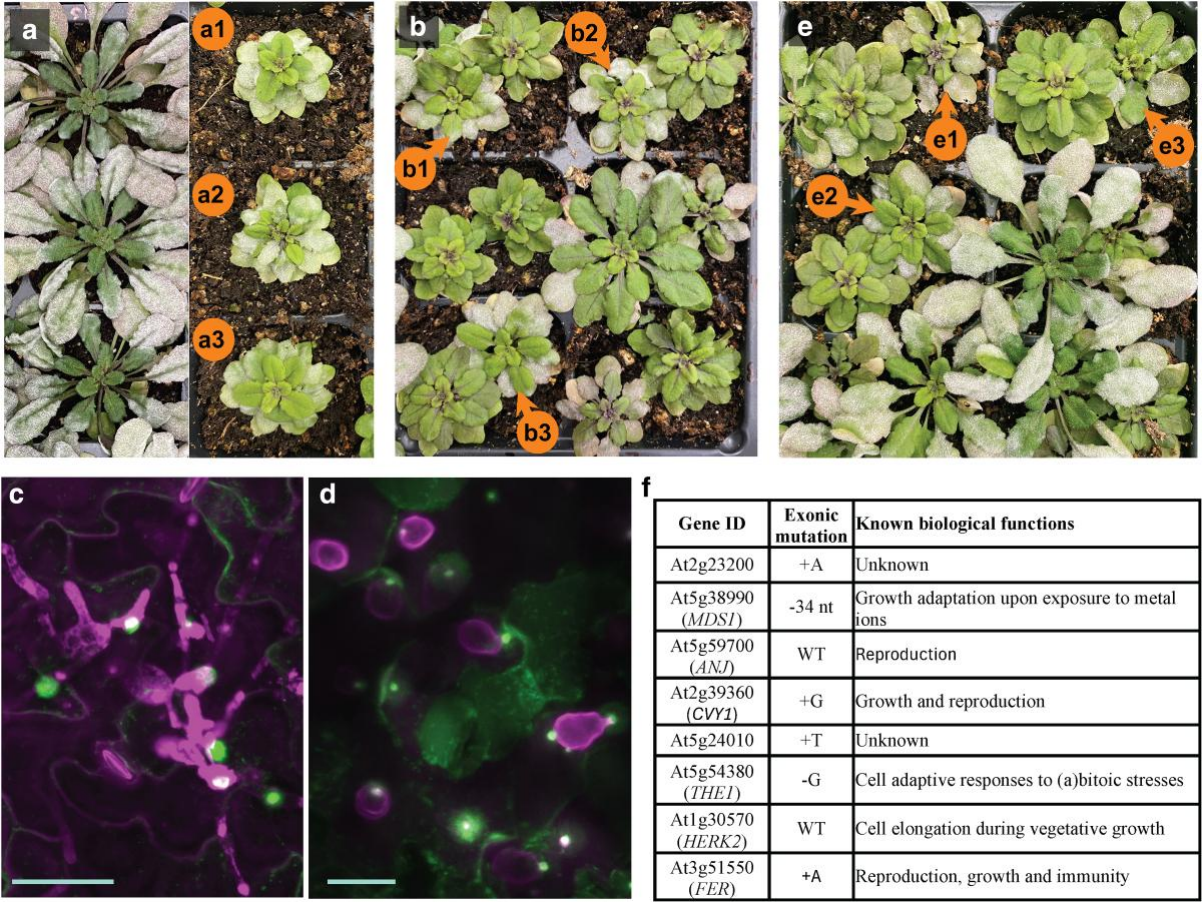
FERONIA and its 5 family members are dispensable for MLO2's focal accumulation and MLO2-mediated susceptibility. CRISPR/Cas9-targeted mutagenesis was used to knock out *Feronia* (*FER*) alone, or *FER* and 5 other members of the *CrRLK1L* gene family in *eps* or *eps3m* plants expressing MLO2-GFP and RPW8.2-RFP. Independent T1 plants were inoculated with *Gc* UCSC1, and photos were taken at 10 to 12 dpi. Mutations in *FER* and other family members were identified by targeted sequencing (see Figures S14 and S15). a) Infection phenotypes of representative *eps* plants and 3 T1 transgenic lines of *eps3* expressing the CRISPR construct targeting *FER* (a1 to a3). Sanger sequencing results are shown in Figure S14a. b) Infection phenotypes of T1 plants of *eps3m/pMLO2-MLO2-GFP/pRPW8.2-RPW8.2-RFP* transgenic for the CRISPR construct targeting *FER*. Three susceptible *fer-*like mutant plants (b1 to b3) were subjected to sequencing analysis of *FER* (see Figure S14b). c and d) Representative confocal images showing typical focal accumulation of MLO2-GFP (c) and EHM-localization of RPW8.2-RFP (d). Images are Z-stack projections of 3 to 5 optical sections. Fungal structures were stained with propidium iodide and visualized in magenta. Bar = 20 μm. e) Infection phenotypes of T1 plants of *eps3m/pMLO2-MLO2-GFP/pRPW8.2-RPW8.2-RFP* transgenic for the CRISPR construct targeting 8 *CrRLK1L* family genes. Three susceptible *fer-*like mutant plants (e1 to e3) were subjected to sequencing analysis to reveal the indel mutations. f) A chart showing the mutations in the 8 *CrRLK1L* genes targeted by CRISPR mutagenesis in line e2 shown in e). The Sanger sequencing chromatograms are shown in Figure S15.

FERONIA (FER), a member of the *Catharanthus roseus* receptor-like kinase 1-like (CrRLK1L) protein subfamily (Escobar-Restrepo et al. 2007; Guo et al. 2009), has been shown to be important for the proper localization of MLO7 in synergid cells (Ju et al. 2021). Additionally, *fer* mutant plants exhibit reduced susceptibility to PM disease (Kessler et al. 2010). Given the molecular functions shared by MLO2 and MLO7 (Fig. 7c and d; Jones et al. 2017), we asked whether FER is also required for the focal localization of MLO2 at the PPM in leaf epidermal cells. To test this, we first performed CRISPR/Cas9-targeted mutagenesis in the *eps* background. Loss-of-function *fer* mutants display a more compact rosette phenotype, readily distinguishable from wild-type plants (Deslauriers and Larsen 2010). We obtained 7 presumable *eps*-*fer* mutants with a compact rosette and found that all of them showed similar levels of susceptibility to *Gc* UCSC1 as *eps* plants (Fig. 9a). Sequencing 3 of the 7 mutants revealed disruptive indel mutations close to the sgRNA target sites in the second exon of *FER* (Figure S14a), predicted to lead to truncated FER proteins. This result indicates that *FER* is dispensable for PM pathogenesis in *eps* and implies that, different from the immunity observed in *eps3m* and *3m*/C mutants (Figs 3 and 4), the previously reported enhanced resistance in *fer* single mutants (Kessler et al. 2010) may be due to activation of EDS1/PAD4/SID-dependent immunity. Next, we introduced the same CRISPR/Cas9 construct into *eps3m* plants transgenic for *MLO2-GFP* and *RPW8.2-RFP* to determine if focal accumulation of MLO2-GFP is affected in the absence of FER. Among 27 T1 transgenic plants obtained, 18 displayed the *fer*-characteristic compact rosette phenotype. Of these 18 plants, 8 were susceptible to *Gc* UCSC1; the remaining 10 plants did not support any growth of *Gc* UCSC1 visible to the naked eye (Fig. 9b). Targeted sequencing of *FER* in 3 of the 8 susceptible plants with a compact rosette identified disruptive indels in *FER*, as expected (Figure S14b). Confocal imaging showed that MLO2-GFP fluorescence was not detectable in the 10 T1 plants with a compact rosette but lacking infection, suggesting that the *MLO2-GFP* transgene is silenced in these lines, which was frequently observed in transgenic plants generated in the *eps3m*-*MLO2-GFP;RPW8.2-RFP* background. Importantly, MLO2-GFP, as well as RPW8.2-RFP, exhibited normal subcellular localization in the 8 T1 plants with a compact rosette and susceptible to the fungus (Fig. 9c and d), indicating that loss of FER does not affect MLO2's localization to the PPM and its role in supporting PM pathogenesis in leaf epidermal cells. To assess if there is functional redundancy among FER and its paralogs, we performed multiplexed CRISPR/Cas9-based mutagenesis to target 8 *CrRLK1L* family members (Fig. 9f), including *FER*, that are known to be involved in immunity and/or expressed in leaves (https://plantrnadb.com/athrdb/). Among 25 T1 transgenic lines, 11 displayed the *fer*-characteristic compact rosette phenotype, among which 5 were susceptible to *Gc* UCSC1 (Fig. 9e). We selected one of the susceptible lines for sequencing of all 8 targeted *CrRLK1L* genes, which revealed that this line contains disruptive mutations in *FER* and 5 additional *CrRLK1Ls* (Fig. 9f; Figure S15). Yet MLO2-GFP's subcellular localization and role in supporting PM entry remained unaffected (Figure S16), indicating that those 6 CrRLK1Ls are dispensable for MLO2's localization and function.

## Discussion

Clade IV/V MLO proteins have long been considered to be host susceptibility or compatibility factors of powdery mildew (Panstruga 2003). Yet, to date, there is no definitive genetic evidence to exclude the ectopic activation of defense as the molecular basis of *mlo*-mediated “resistance” despite the fact that no specific defense pathways or components have rigorously been shown to contribute to the near-complete resistance of the *mlo2/mlo6/mlo12* triple mutants (Kuhn et al. 2017). In this study, using a tailored forward genetic screen designed to identify mutants that display *cipi* phenotypes, we found that disruptive mutations in *MLO2* underlie the *cipi* phenotype of the 5 mutants with the strongest resistance phenotypes. Subsequent CRISPR/Cas9-mediated mutagenesis of *MLO6* and *MLO12* in the *cipi3* background, along with downstream analyses, further revealed that *mlo2/6/12*-conditioned suppression of mildew infection can be uncoupled from the ectopic activation of canonical defense pathways. These findings support the notion that MLO proteins might be bona fide host susceptibility factors that are essential for PM pathogenesis. Moreover, leaf expression analyses of 7 other MLO family members (MLO1, MLO3, MLO4, MLO5, MLO7, MLO10, and MLO11) from the *MLO2* promoter revealed that, except for MLO7, none could significantly compensate for the loss of MLO2, MLO6, and MLO12, highlighting functional diversification within the MLO family in Arabidopsis.

### *mlo*-mediated resistance is independent of activation of canonical defense programs

*mlo*-mediated resistance to PM fungi has long been linked to defense-associated phenotypes, including increased spontaneous callose deposition, early leaf senescence, and reduced plant stature (Wolter et al. 1993; Piffanelli et al. 2002; Consonni et al. 2006; Humphry et al. 2006; Wan et al. 2020). Previous genetic studies in Arabidopsis indicated that *mlo2*-mediated resistance partially requires SA-dependent and SA-independent defenses (Consonni et al. 2006) and correlates with the increased production of antifungal indolic glucosinolates (Consonni et al. 2010). However, no specific plant defense pathways were found to be indispensable for the near-complete resistance of the *mlo2/6/12* triple mutants (Kuhn et al. 2017), leading to the speculation that a broad and fast activation of immune responses in *mlo2 mlo6 mlo12* plants can compensate for the lack of a single or a few defense pathways (Acevedo-Garcia et al. 2017a). In this study, we discovered that *mlo2*-mediated partial resistance and *mlo2/6/12*-mediated near-complete resistance to the adapted PM isolate *Gc* UCSC1 and the nonadapted PM isolate *Gc* UMSG1 are independent of EDS1/PAD4/SID2, 3 key immune components (Figs 1 to 3). We further demonstrated that loss of MLO2, MLO6, and MLO12 conferred near-complete resistance to adapted and nonadapted PM fungi in the background of the *eds1/pad4/sid2/pen1/pen2/pen3* mutant (Figure S4). Our results regarding ROS production and senescence-associated cell death (Fig. 4) as well as expression analyses of defense reporter genes (Figure S5) further support the notion that *mlo*-mediated resistance to PM fungi can be uncoupled from activation of major SA-dependent defenses. Interestingly, Munch et al. (2026) very recently reported that *mlo2/6/12*-mediated near-complete penetration resistance against adapted PM isolate can be partially overcome in a genetic background where the 2 syntaxin genes *SYP121* (*PEN1*) and *SYP122*, as well as the *FMO1* immunity gene, are mutated, and a bacterial SA hydroxylase gene *NahG* is ectopically overexpressed (Munch et al. 2026). Notably, despite the remarkably high penetration success observed in this transgenic line (∼45%, compared with ∼80% in *syp121/syp122/fmo1* and nearly 0% in *mlo2/6/12/fmo1*), the adapted PM fungus exhibited only very limited secondary hyphal growth (ie less than twice the length of the conidium) and was invariably arrested at this early developmental stage (Munch et al. 2026). Thus, these findings do not entirely contradict our proposal that clade V MLO2, MLO6, and MLO12 in Arabidopsis may serve as bona fide susceptibility factors required for PM pathogenesis. We cannot, however, rule out the possibility that the strong PM resistance phenotype of *mlo2/6/12* mutant plants arises from activation of defense pathways partially dependent on SYP121/122-regulated trafficking.

### Localization of MLO2 may hint at its role in PM pathogenesis

What, then, is absent in Arabidopsis mutants lacking MLO2, MLO6, and MLO12 that is indispensable for PM pathogenesis? Because PM fungi strictly depend on living host cells for successful invasion, we propose that they exploit an MLO-dependent host cellular process to enable penetration and haustorium differentiation. In this context, the accumulation of MLO2-GFP to the PPM, the extracellular membrane domain (Fig. 6h and i) that matches mostly or exactly the same physical space as the papilla where callose is deposited (Fig. 6g), or the paramural body where the syntaxin PEN1 (SYP121) exosomes accumulate (Assaad et al. 2004; Bhat et al. 2005; Meyer et al. 2009), may provide important mechanistic insights. Upon spore inoculation, the earliest focal accumulation of MLO2-GFP was detected at 7.5 hpi (Fig. 6c and d), well before haustorium formation, which happens at 12 to 14 hpi (Koh et al. 2005). Given that germinated sporelings can develop normal appressoria and penetration pegs in *eps3m* (pointed by an arrow in Fig. 3b) but fail to differentiate haustoria in pavement epidermal cells (Fig. 3c), the accumulation of a sufficient amount of functional clade V MLOs in the PPM may be required for haustorial differentiation. MLOs may be exocytosed into the PPM and function as scaffolding crucial for stabilizing and resealing the plasma membrane damaged by penetration, thereby enabling haustorium differentiation from the tip of the penetration peg (Fig. 6f to i). In addition, a poorly characterized membranous structure, the haustorial neckband (which is indicated as a blue band in Fig. 6i), is thought to form before or concomitantly with haustorial biogenesis and to seal the space between the apoplast and the extra-haustorial matrix (Gil and Gay 1977; Bushnell and Gay 1978). MLO2 may also be localized to the haustorial neckband and/or required for its formation. Higher-resolution microscopy with immunogold labeling could help assess this possibility. It is worth noting that our MLO2-GFP localization analyses were performed in immunocompromised *eps3m* plants expressing *pMLO2::MLO2-GFP*. Consequently, the spatiotemporal dynamic distribution of MLO2-GFP may differ to some extent in Col-0 or *3m*/C genetic backgrounds. Furthermore, given the proposed calcium channel activity of MLO2 (Gao et al. 2022, 2023), MLO2-mediated localized calcium influx may be necessary for the resealing and stabilization of the PPM junction, including neckband formation, thereby permitting haustorium differentiation inside a host cell.

### Functional diversification within the MLO family in Arabidopsis

Different MLO family members are known to fulfill distinct biological roles (Li and Xiao 2025). However, it remains unclear whether other members can perform the same or similar molecular functions of MLO2, MLO6, and MLO12 when expressed in leaf epidermal cells. Results from our analyses through *MLO* transgene expression driven by the *MLO2* promoter demonstrate that there is clear functional diversification among MLO11 (clade I), MLO1 (clade II), MLO7 (clade III), and MLO2 and MLO6 (clade V), both in terms of subcellular localization and their ability to enable PM pathogenesis (Fig. 7). While MLO2, MLO6, and MLO7 were also found in the plasma membrane of leaf epidermal cells (Figs 6e, 7b and d), they mainly exhibit accumulation in puncta and relocalize to the PPM in PM-challenged cells to accommodate PM pathogenesis. By contrast, MLO1 and MLO11 are homogenously localized at the plasma membrane, show no obvious focal accumulation at the PPM, and cannot substitute MLO2 in permitting PM pathogenesis (Fig. 7f and h). Given that MLO1, MLO2, and MLO7 all exhibit calcium channel activity when expressed in animal cells (Gao et al. 2022, 2023), their functional diversification may stem from their regulation by different interacting proteins, rather than from their ability to conduct calcium ions across the plasma membrane. Alternatively, they might not possess calcium channel activity in epidermal cells and/or such activity may not be important for their biological function(s).

Results from domain swapping between MLO1 and MLO2 (Fig. 8) and between MLO1 and MLO7 (Jones et al. 2017) suggest that there are at least 2 functional regions in MLO proteins: the N-terminal, 7TM domain-containing portion performs the cellular function of a particular MLO protein, whereas the cytoplasmic C-terminus specifies its subcellular localization. Our identification of the E7K mutation in MLO2 as the causal mutation in *cipi12* (Fig. 2a and f) renders support to this inference. Such a functional separation can explain the activity of chimeric proteins such as MLO2n-1c (in conferring partial susceptibility of *eps3m* to *Gc* UCSC1 [Fig. 8f and g]) and NTA (MLO7)-MLO1^Cterm^ (also named faNTA, in restoration of fertility of *mlo7* mutant plants [Ju et al. 2021]). Notably, because MLO2n-1c does not show significant focal accumulation, its presence in the PPM is likely limited. This probably explains the weak susceptibility of *eps3m* lines expressing MLO2n-1c to *Gc* UCSC1, underscoring the importance of a “dosage” effect through MLO2's C-terminal domain-mediated focal accumulation. This also aligns with the observed correlation between MLO2 expression levels and the degree of susceptibility of transgenic lines (Fig. 5). On the other hand, MLO1n-2c exhibited punctate distribution and focal accumulation induced by sporelings in *eps* (Fig. 8e), but it did not confer susceptibility in *eps3m* plants (Fig. 8b and c), supporting the requirement of the MLO2 C-terminus for focal accumulation.

It is also worth noting that MLO10, MLO7's closest paralog in clade III, displayed accumulation in puncta (Fig. 7j and l) but only allowed very limited PM growth in the best-case scenario (Fig. 7l). In synergid cells, MLO10 but not MLO8 (which also belongs to clade III), and surprisingly MLO2 (clade V), could restore fertility in *mlo7* mutant plants (Jones et al. 2017). Combined, these observations suggest that (i) there is clear functional diversification among these 3 clade III family members and (ii) some sequence or structural features shared by MLO2 and MLO7, but not by MLO8 and MLO10 (to a lesser extent), may underscore their functional exchangeability. Future studies may be directed to revealing this commonality between MLO2 and MLO7. Given this complexity and the lack of localization data for MLO3, MLO4, and MLO5 in Arabidopsis leaf epidermal cells, it is challenging to infer which sequence features or polymorphisms underlie the contrasting molecular functions of MLOs as reflected by permitting PM pathogenesis and displaying stimulus-induced subcellular relocalization.

### Regulation of MLO2's focal accumulation at the PPM

Regarding PM penetration-induced MLO2 subcellular relocalization to the PPM (Fig. 6), a key question is what specific signal(s) trigger(s) its polarizing trafficking and how this process is regulated. Given that MLO2's CaMBD-containing cytoplasmic C-terminus directs its focal accumulation, it is conceivable that changes in cytosolic calcium concentration—triggered by calcium influxes during fungal penetration of the host cell wall or invagination of the host plasma membrane—could induce MLO2-calmodulin binding or dissociation, thereby triggering MLO2's redistribution to the PPM. Such a mechanism would imply that changes of cytosolic calcium levels differentially impact MLOs residing in different subcellular compartments (eg MLO2 mostly at the ER/Golgi versus MLO1 at the plasma membrane), likely due to structural differences in their C-termini and specific calmodulins they interact with. In this context, it is worth noting that MLO1n-2c remained localized in puncta in leaf epidermal cells of *eps3m* mutant plants insulted by fungal sporelings (Fig. 8c). However, MLO1n-2c were relocalized to the PPM in the *eps* mutant (Fig. 8g), suggesting that host cell wall or plasma membrane disruption induced by successful pathogen entry triggers a PPM-oriented trafficking pathway, resulting in dispatch of ER/Golgi-localized MLO2 and the nonfunctional MLO1n-2c to the PPM. Because successful host cell entry requires functional MLO2, this would imply a positive feedback mechanism for MLO2's focal accumulation at the PPM. Without functional plasma membrane-localized MLO2 to initiate the ER/Golgi-to-PPM-directed trafficking, MLO1n-2c would remain sequestered at the ER/Golgi.

How MLO2's ER/Golgi-to-PPM trafficking polarity is established remains unknown. In synergid cells, FER is required for MLO7's subcellular relocalization from Golgi bodies to the filiform apparatus in synergid cells (Jones et al. 2017; Ju et al. 2021). However, our genetic data indicate that FER and probably 5 other CrRLK1L family members are dispensable for MLO2's focal accumulation and its mediated PM pathogenesis (Fig. 9). This finding suggests that the cellular environment of leaf epidermis may obviate the need of FER for polarized trafficking of MLO2 to the PPM. As such, this may reflect previously unrecognized functional redundancy within the 17-member CrRLK1L family (Lindner et al. 2012), or the involvement of a CrRLK1L-independent regulatory mechanism, such as phosphorylation by a distinct kinase, in leaf epidermal cells that differs from the pathway mediating MLO7 relocalization in synergid cells.

In conclusion, data from this study strongly support that MLO2, MLO6, and MLO12 are bona fide susceptibility factors of PM fungi, uncoupling the failure of PM pathogenesis due to the loss of these 3 MLOs from the activation of canonical defense programs. MLO2 (and, by inference, other clade IV and V MLOs) appears to adopt a bipartite functional configuration: While the N-terminal 7TM domain-containing portion executes a specific cellular function, the CaMBD-containing cytoplasmic C-terminus orchestrates its spatiotemporal activity via stimulus-induced subcellular relocalization and enrichment. In line with the functional diversification of MLOs in different clades, the requirement of FER for the relocalization of MLO2 and MLO7 differs greatly: Whereas FER plays a key role in MLO7's enrichment in the filiform apparatus of the synergid cell upon reception of the pollen tube (Gao et al. 2022, 2023), FER and 5 other CrRLK1L family members are dispensable for the focal accumulation of MLO2 at the PPM. Future work will investigate how clade IV/V MLO proteins focally accumulate and function at the PPM to permit PM pathogenesis.

## Materials and methods

### Plant lines and growth conditions

All mutants used in this study were in the background of *A. thaliana* accession Col-0. Mutants *eds1-2* (Bartsch et al. 2006), *pad4-1* (Jirage et al. 1999), and *sid2-2* (Wildermuth et al. 2001) have been previously described. The *eds1-2/pad4-1/sid2-2* triple mutant was generated by genetic crosses. The *mlo2-5/mlo6-2/mlo12-1* triple mutant was previously described (Consonni et al. 2006). Seeds were sown in SunGro Horticulture (Agawam, MA, United States) and cold-treated (4 ℃ for 2 d) before moving to growth chambers. Seedlings were transplanted and kept growing under 22 ℃, 75% relative humidity, short day (8 h light at ∼125 μmol m^−2^ s^−1^, 16 h dark) conditions for up to 14 wk before use.

### EMS mutagenesis of seeds and mutant screening

About 10,000 *eps* seeds (approx. 200 mg) were placed in a 250 mL glass flask. Then, 15 mL dH_2_O and 30 μL 0.2% EMS were added, and the seeds were shaken overnight. After washing with dH_2_O, seeds were blotted dry and mixed with 250 g fine sand and aliquoted into about 50 parts. Each aliquot of ∼5 g sand with seeds was sown evenly in SunGro Horticulture (Agawam, MA, United States) in 50 flats. Seedlings were grown in a greenhouse for about 2 mo until maturity. Seeds from ∼90 M1 plants were collected to make 1 M1 seed pool. A total of 102 M1 seed pools were obtained. For mutant screening, about ∼450 M2 plants per pool were prepared in 2 flats for inoculation with *Gc* UCSC1. Putative *cipi* mutants were transplanted into individual pots for further growth till maturity.

### Pathogen infection, disease phenotyping, and quantification

The Arabidopsis-adapted powdery mildew isolate *G. cichoracearum* (*Gc*) UCSC1 was maintained on live Col-0 or *eds1-2* plants. The nonadapted *Gc* UMSG1 was maintained on its natural host, sow thistle (Wen et al. 2011). Inoculation, visual scoring of disease reaction phenotypes, and spore quantification were done as previously described (Xiao et al. 2005).

### Mapping of causal mutations

Bulked segregant pool-genome sequencing was used to identify candidate causal mutations. *cipi2* and *cipi3* mutants were crossed with the *eps* parental line, and their corresponding F2 segregating populations were inoculated with *Gc* UCSC1. Genomic DNA was isolated from the mixture of the leaf tissues harvested from 65 or more F2 individuals showing the *cipi* phenotypes using NucleoSpin Plant II kit (MACHEREY-NAGEL, #740770.50). About 2 μg of each DNA sample was sent to BGI (Beijing Genomics Institute, Shenzhen, China) for deep sequencing using Illumina Hiseq 2000 platform at an average coverage of ∼50× with 100 bp paired-end reads. The subsequent sequence analysis was done according to the method previously described (Wang et al. 2018). Briefly, sequencing reads were mapped against the TAIR10 Arabidopsis reference genome using Bowtie (Langmead et al. 2009) and variants were called by SAMtools (Li et al. 2009). Only G-to-A and C-to-T conversions predominantly caused by EMS mutagenesis were picked for further analysis. The effect of each EMS-induced SNP (single-nucleotide polymorphism) on the corresponding gene was annotated using snpEffect (Cingolani et al. 2012). The effects of the SNPs were classified into 3 categories: very high (stop gained, splice site donor, splice site acceptor), high (nonsynonymous coding, start gained, stop lost), and other (intergenic, intron, 3′ UTR, 5′ UTR, synonymous coding).

### DNA constructs and Arabidopsis transformation

The pK7FWG2 plasmid (Karimi et al. 2002) was used for cloning of all *MLO* genes in translational fusion with eGFP. To replace the original *35S* promoter with the *MLO2* native promoter (*pMLO2*), restriction enzymes *Xba*I and *Bam*HI were used to linearize the plasmid, and a ∼2 kb *pMLO2* fragment amplified from genomic DNA was inserted, leading to a *pMLO2::ccdB-eGFP* cassette used for cloning *MLOx-eGFP* fusion genes under control of the *MLO2* promoter. To create various *MLO* expression constructs, corresponding *MLO* genes were first cloned into the pENTR/D-TOPO vector via TOPO cloning (https://www.thermofisher.com/us/en/home/life-science/cloning/topo.html). Then, the *MLO* genes were shuttled to the binary vector containing *pMLO2::ccdB-eGFP* via Gateway LR reaction. All vectors were verified by Sanger sequencing.

Amplification of fragments for the creation of chimeric *MLOs* was done using primers in Table S1 by overlap-extension PCR. Specifically, the 2 fragments were amplified from Col-0 cDNA with overlapped chimeric primers. The 2 products were then mixed at the same molar concentration and served as a template for the amplification of the full-length chimeric gene. All vectors were verified by Sanger sequencing.

All binary vectors containing the *MLOx-eGFP* constructs were transfected into *Agrobacterium tumefaciens* GV3101. Arabidopsis transformation was conducted following the floral dipping protocol described previously (Clough and Bent 1998).

### Agrobacterium-mediated transient expression in *N. benthamiana* leaves

*N. benthamiana* plants were grown in a growth chamber under 22 ℃, 75% relative humidity, long day (16 h light at ∼125 μmol m^−2^ s^−1^, 8 h dark) conditions for 4 wk, and then were moved to short day conditions (8 h light at ∼125 μmol m^−2^ s^−1^, 16 h dark) for 1 wk before agroinfiltration. *A. tumefaciens* GV3101 cells containing corresponding vector(s) were suspended in infiltration buffer (10 mM MgCl_2_, 10 mM MES [pH 5.6], and 200 μM acetosyringone) to a final OD_600_ value of 0.4 to 0.6. The agrobacterium suspension was incubated in the dark for 2 h before infiltration into *N. benthamiana* leaves using a blunt syringe. The agroinfiltrated leaves were examined for MLOx-eGFP expression by confocal microscopy following the method described below.

### Confocal microscopy

The expression and localization of the MLOx-eGFP fusion proteins and RPW8.2-RFP were examined by confocal microscopy using a Zeiss LSM710 microscope. The methods for revealing fungal structures using propidium iodide, the plasma membrane using FM4-64, and the callose using sirofluor were the same as previously reported (Berkey et al. 2017). Saturated pixels are intentionally avoided. Confocal images were post-processed using Zeiss ZEN Microscopy Software with global linear adjustments applied consistently and in accordance with academic best practices for image processing.

### Detection of H_2_O_2_ accumulation and cell death

DAB (3,3′-diaminobenzidine) staining was used to detect in situ H_2_O_2_ production and accumulation, while trypan blue staining was used to detect dead or dying cells as well as fungal structures in leaves. These methods were previously described (Xiao et al. 2003).

### Reverse transcription quantitative polymerase chain reaction (RT-qPCR) analysis

Three leaf samples of 7-wk-old plants (∼100 mg) per genotype were harvested before and at 0, 6, 12, and 48 hpi after *Gc* UCSC1 infection. Total RNA was extracted using TRIzol Reagent and reverse transcribed into cDNA using SuperScript III Reverse Transcriptase (Invitrogen, Thermo Fisher Scientific Inc.). For each experiment, RT-qPCR was performed with 3 biological replicates per treatment and 3 technical replicates per sample using the Applied Biosystems 7300 Real-Time PCR System with SYBR^TM^ Green PCR Master Mix (Thermo Fisher Scientific Inc.). The transcript levels of the target genes were normalized to those of *UBC9* (*Ubiquitin conjugating enzyme 9*, *AT4G27960*). Data were analyzed using the Applied Biosystems 7300 Real-Time PCR System Software and the comparative ΔΔCt method. Primers used for RT-qPCR are listed in Table S2.

### Immunoblot

Leaf tissue (150 mg per sample) was frozen in liquid nitrogen and ground into fine powder. Two volumes (w/v) Ripa buffer containing 100 μM PMSF, 1× protease inhibitor, and 100 mM DTT was added to the frozen sample and then vortexed for homogenization. Samples were incubated at 95 ℃ for 5 min in 1× SDS sample buffer, then centrifuged at 12,000 × *g* for 10 min. Samples were loaded onto a 4% to 12% Bis-Tris SurePAGE gel (GenScript #M00652) for electrophoresis following the manufacturer's instructions. Membrane transfer was conducted at 100 V for 2 h. The membrane was blocked with 5% BSA in TBST for 1 h and then incubated with anti-GFP antibody (ab290, Abcam) overnight at 4 ℃. After 3× 10 min washing with TBST buffer (20 mM Tris and 150 mM NaCl with 0.1% Tween-20, pH 7.4), the membrane was loaded with secondary antibody and incubated at room temperature for 1 h with shaking, followed by 3×10 min wash. The signal was generated by Clarity Western ECL Substrate (BIO-RAD #1705061) and imaged with a BIO-RAD ChemiDoc Imaging System.

### CRISPR/Cas9-targeted mutagenesis

Two CRISPR/Cas9 genome editing systems were used for targeted mutagenesis of genes of interest in this study, and all related plasmids were purchased from Addgene (https://www.addgene.org). The first utilizes an Arabidopsis egg cell-specific promoter to drive the expression of Cas9 (Wang et al. 2015). This system was used to knock out *MLO2*, *MLO6*, and *MLO12*. The other system was developed for multiplexed CRISPR/Cas9-based mutagenesis (Stuttmann et al. 2021). It utilizes an “intronized” *Cas9* nuclease gene to improve Cas9 expression and editing efficiency. This system was used to knock out *FER* and its related *CrRLKL1* family members. The pDGE347 binary destination vector was used for assembling the guide RNA constructs. All recombinant plasmids containing the guide RNA cassettes were confirmed by sequencing. All guide RNA sequences were listed in Table S3.

### Genotyping of mutants and transgenic lines

All primers used for genotyping are listed in Table S4. To detect the *mlo2* allele from *cipi2*, the fragment containing the mutation was amplified by MLO2-e9F/MLO2-e11R followed by *Mwo*I digestion (New England Biolabs, R0573S). The wild-type allele is cut into 2 smaller fragments (161 + 242 bp), while the *cipi2* mutant allele is intact (403 bp). Similarly, to detect the *mlo2* allele from *cipi3*, the fragment containing the mutation was amplified by MLO2-e2F/MLO2-e3R followed by *Hind*III digestion (New England Biolabs, R3104S). While the wild-type allele is intact (341 bp), the *cipi3* mutant allele is digested into 2 fragments (179 + 162 bp). The remaining *mlo2* alleles *cipi11*, *cipi12*, and *cipi15* were amplified with MLO2e2F/MLO2-e3R, MLO2-e1F/MLO2-e3R, and MLO2-e6F/MLO2-e8R, respectively, followed by sequencing to detect the respective mutations. To detect mutations in *FER* and its family members, the guide RNA target regions were amplified with gene-specific primers (listed in Table S4). PCR products were Sanger-sequenced. Benchling (https://benchling.com/), a cloud-based platform for molecular biology data analysis, was used to make sequence alignments for the identification of indels.

### Accession numbers

DNA sequences of the *A. thaliana* genes studied in this study can be found in the National Center for Biotechnology Information (NCBI) or The Arabidopsis Information Resource under the following gene ID numbers: *AT4G02600* (*MLO1*), *AT1G11310* (*MLO2*), *AT3G45290* (*MLO3*), *AT1G11000* (*MLO4*), *AT2G33670* (*MLO5*), *AT1G61560* (*MLO6*), *AT2G17430* (*MLO7*), *AT5G65970* (*MLO10*), *AT5G53760* (*MLO11*), *AT2G39200* (*MLO12*), *AT2G19190* (*FRK1*), *AT5G44420* (*PDF1.2*), *AT5G14930* (*SAG101*), *AT2G14610* (*PR1*), *AT5G38990* (*MDS1*), *AT5G59700* (*ANJ*), *AT5G54380* (*THE1*), *AT1G30570* (*HERK2*), *AT3G51550* (*FER*), *AT2G39360* (*CVY1*), and unnamed *CrRLK1L* family members, *AT2G23200* and *AT5g24010*.

## Supplementary Material

kiag413_Supplementary_Data

## Data Availability

The data that support the findings of this study are available from the corresponding author upon request.

## References

[kiag413-B1] Acevedo-Garcia J et al 2017a. The powdery mildew-resistant Arabidopsis *mlo2 mlo6 mlo12* triple mutant displays altered infection phenotypes with diverse types of phytopathogens. Sci Rep. 7:9319. 10.1038/s41598-017-07188-7. 28839137 PMC5570895

[kiag413-B2] Acevedo-Garcia J et al 2017b. *mlo*-based powdery mildew resistance in hexaploid bread wheat generated by a non-transgenic TILLING approach. Plant Biotechnol J. 15:367–378. 10.1111/pbi.12631. 27565953 PMC5316926

[kiag413-B3] Acevedo-Garcia J, Kusch S, Panstruga R. 2014. Magical mystery tour: MLO proteins in plant immunity and beyond. New Phytol. 204:273–281. 10.1111/nph.12889. 25453131

[kiag413-B4] Asai T et al 2002. MAP kinase signalling cascade in Arabidopsis innate immunity. Nature. 415:977–983. 10.1038/415977a. 11875555

[kiag413-B5] Assaad FF et al 2004. The PEN1 syntaxin defines a novel cellular compartment upon fungal attack and is required for the timely assembly of papillae. Mol Biol Cell. 15:5118–5129. 10.1091/mbc.e04-02-0140. 15342780 PMC524786

[kiag413-B6] Bartsch M et al 2006. Salicylic acid-independent ENHANCED DISEASE SUSCEPTIBILITY1 signaling in Arabidopsis immunity and cell death is regulated by the monooxygenase FMO1 and the Nudix hydrolase NUDT7. Plant Cell. 18:1038–1051. 10.1105/tpc.105.039982. 16531493 PMC1425861

[kiag413-B7] Berkey R et al 2017. Homologues of the RPW8 resistance protein are localized to the extrahaustorial membrane that is likely synthesized de novo. Plant Physiol. 173:600–613. 10.1104/pp.16.01539. 27856916 PMC5210751

[kiag413-B8] Bhat RA, Miklis M, Schmelzer E, Schulze-Lefert P, Panstruga R. 2005. Recruitment and interaction dynamics of plant penetration resistance components in a plasma membrane microdomain. Proc Natl Acad Sci U S A. 102:3135–3140. 10.1073/pnas.0500012102. 15703292 PMC549507

[kiag413-B9] Bidzinski P et al 2014. Physiological characterization and genetic modifiers of aberrant root thigmomorphogenesis in mutants of *Arabidopsis thaliana MILDEW LOCUS O* genes. Plant Cell Environ. 37:2738–2753. 10.1111/pce.12353. 24738718

[kiag413-B10] Bravo A, York T, Pumplin N, Mueller LA, Harrison MJ. 2016. Genes conserved for arbuscular mycorrhizal symbiosis identified through phylogenomics. Nat Plants. 2:15208. 10.1038/nplants.2015.208. 27249190

[kiag413-B11] Büschges R et al 1997. The barley *Mlo* gene: a novel control element of plant pathogen resistance. Cell. 88:695–705. 10.1016/S0092-8674(00)81912-1. 9054509

[kiag413-B12] Bushnell WR, Gay JL. 1978. Accumulation of solutes in relation to the structure and function of haustoria in powdery mildews. In: Spencer DM, editors. The powdery mildews. Academic Press. p. 183–235.

[kiag413-B13] Chen Z et al 2006. Expression analysis of the *AtMLO* gene family encoding plant-specific seven-transmembrane domain proteins. Plant Mol Biol. 60:583–597. 10.1007/s11103-005-5082-x. 16525893

[kiag413-B14] Chen Z et al 2009. Two seven-transmembrane domain MILDEW RESISTANCE LOCUS O proteins cofunction in Arabidopsis root thigmomorphogenesis. Plant Cell. 21:1972–1991. 10.1105/tpc.108.062653. 19602625 PMC2729597

[kiag413-B15] Cingolani P et al 2012. A program for annotating and predicting the effects of single nucleotide polymorphisms, SnpEff: SNPs in the genome of Drosophila melanogaster strain w1118; iso-2; iso-3. Fly (Austin). 6:80–92. 10.4161/fly.19695. 22728672 PMC3679285

[kiag413-B16] Clough SJ, Bent AF. 1998. Floral dip: a simplified method for Agrobacterium-mediated transformation of *Arabidopsis thaliana*. Plant J. 16:735–743. 10.1046/j.1365-313x.1998.00343.x. 10069079

[kiag413-B17] Consonni C et al 2006. Conserved requirement for a plant host cell protein in powdery mildew pathogenesis. Nat Genet. 38:716–720. 10.1038/ng1806. 16732289

[kiag413-B18] Consonni C et al 2010. Tryptophan-derived metabolites are required for antifungal defense in the Arabidopsis *mlo2* mutant. Plant Physiol. 152:1544–1561. 10.1104/pp.109.147660. 20023151 PMC2832281

[kiag413-B19] Cui H et al 2022. *CmPMRl* and *CmPMrs* are responsible for resistance to powdery mildew caused by *Podosphaera xanthii* race 1 in melon. Theor Appl Genet. 135:1209–1222. 10.1007/s00122-021-04025-4. 34989827

[kiag413-B20] Davis TC et al 2017. *Arabidopsis thaliana MLO* genes are expressed in discrete domains during reproductive development. Plant Reprod. 30:185–195. 10.1007/s00497-017-0313-2. 29159588

[kiag413-B21] Deslauriers SD, Larsen PB. 2010. FERONIA is a key modulator of brassinosteroid and ethylene responsiveness in Arabidopsis hypocotyls. Mol Plant. 3:626–640. 10.1093/mp/ssq015. 20400488

[kiag413-B22] Elliott C et al 2005. Conserved extracellular cysteine residues and cytoplasmic loop-loop interplay are required for functionality of the heptahelical MLO protein. Biochem J. 385:243–254. 10.1042/BJ20040993. 15352871 PMC1134693

[kiag413-B23] Escobar-Restrepo J-M et al 2007. The FERONIA receptor-like kinase mediates male-female interactions during pollen tube reception. Science. 317:656–660. 10.1126/science.1143562. 17673660

[kiag413-B24] Falk A et al 1999. EDS1, an essential component of *R* gene-mediated disease resistance in Arabidopsis has homology to eukaryotic lipases. Proc Natl Acad Sci U S A. 96:3292–3297. 10.1073/pnas.96.6.3292. 10077677 PMC15935

[kiag413-B25] Feechan A, Jermakow AM, Torregrosa L, Panstruga R, Dry IB. 2008. Identification of grapevine *MLO* gene candidates involved in susceptibility to powdery mildew. Funct Plant Biol. 35:1255–1266. 10.1071/FP08173. 32688872

[kiag413-B26] Freh M, Reinstadler A, Neumann KD, Neumann U, Panstruga R. 2024. The development of pleiotropic phenotypes in powdery mildew-resistant barley and *Arabidopsis thaliana mlo* mutants is linked to nitrogen availability. Plant Cell Environ. 47:2362–2376. 10.1111/pce.14884. 38515393

[kiag413-B27] Frye CA, Innes RW. 1998. An Arabidopsis mutant with enhanced resistance to powdery mildew. Plant Cell. 10:947–956. 10.1105/tpc.10.6.947. 9634583 PMC144036

[kiag413-B28] Gao Q et al 2022. A receptor-channel trio conducts Ca^2+^ signalling for pollen tube reception. Nature. 607:534–539. 10.1038/s41586-022-04923-7. 35794475 PMC9308748

[kiag413-B29] Gao Q et al 2023. RALF signaling pathway activates MLO calcium channels to maintain pollen tube integrity. Cell Res. 33:71–79. 10.1038/s41422-022-00754-3. 36588121 PMC9810639

[kiag413-B30] Ge C et al 2020. Physiological changes in barley *mlo-11* powdery mildew resistance conditioned by tandem repeat copy number. Int J Mol Sci. 21:8769. 10.3390/ijms21228769. 33233522 PMC7699567

[kiag413-B31] Gil F, Gay JL. 1977. Ultrastructural and physiological properties of the host interfacial components of the haustoria of *Erysiphe pisi in vivo* and *in vitro*. Physiol Plant Pathol. 10:1–12. 10.1016/0048-4059(77)90002-9.

[kiag413-B32] Guo H et al 2009. Three related receptor-like kinases are required for optimal cell elongation in *Arabidopsis thaliana*. Proc Natl Acad Sci U S A. 106:7648–7653. 10.1073/pnas.0812346106. 19383785 PMC2678668

[kiag413-B33] He Y, Gan S. 2002. A gene encoding an acyl hydrolase is involved in leaf senescence in Arabidopsis. Plant Cell. 14:805–815. 10.1105/tpc.010422. 11971136 PMC150683

[kiag413-B34] Hübbers JW et al 2024. Interplay of EXO70 and MLO proteins modulates trichome cell wall composition and susceptibility to powdery mildew. Plant Cell. 36:1007–1035. 10.1093/plcell/koad319. 38124479 PMC10980356

[kiag413-B35] Humphry M, Consonni C, Panstruga R. 2006. mlo-based powdery mildew immunity: silver bullet or simply non-host resistance? Mol Plant Pathol. 7:605–610. 10.1111/j.1364-3703.2006.00362.x. 20507473

[kiag413-B36] Jacobs AK et al 2003. An Arabidopsis callose synthase, GSL5, is required for wound and papillary callose formation. Plant Cell. 15:2503–2513. 10.1105/tpc.016097. 14555698 PMC280557

[kiag413-B37] Jacott CN, Charpentier M, Murray JD, Ridout CJ. 2020. Mildew locus O facilitates colonization by arbuscular mycorrhizal fungi in angiosperms. New Phytol. 227:343–351. 10.1111/nph.16465. 32012282 PMC7317859

[kiag413-B38] Jacott CN, Ridout CJ, Murray JD. 2021. Unmasking mildew resistance locus O. Trends Plant Sci. 26:1006–1013. 10.1016/j.tplants.2021.05.009. 34175219

[kiag413-B39] Jing H-C et al 2008. Early leaf senescence is associated with an altered cellular redox balance in Arabidopsis *cpr5/old1* mutants. Plant Biol (Stuttg). 10:85–98. 10.1111/j.1438-8677.2008.00087.x. 18721314

[kiag413-B40] Jirage D et al 1999. *Arabidopsis thaliana PAD4* encodes a lipase-like gene that is important for salicylic acid signaling. Proc Natl Acad Sci U S A. 96:13583–13588. 10.1073/pnas.96.23.13583. 10557364 PMC23991

[kiag413-B41] Jones DS et al 2017. MILDEW RESISTANCE LOCUS O function in pollen tube reception is linked to its oligomerization and subcellular distribution. Plant Physiol. 175:172–185. 10.1104/pp.17.00523. 28724621 PMC5580752

[kiag413-B42] Jones DS, Kessler SA. 2017. Cell type-dependent localization of MLO proteins. Plant Signal Behav. 12:e1393135. 10.1080/15592324.2017.1393135. 29039994 PMC5703261

[kiag413-B43] Ju Y et al 2021. Polarized NORTIA accumulation in response to pollen tube arrival at synergids promotes fertilization. Dev Cell. 56:2938–2951.e6. 10.1016/j.devcel.2021.09.026. 34672969

[kiag413-B44] Karimi M, Inze D, Depicker A. 2002. GATEWAY vectors for Agrobacterium-mediated plant transformation. Trends Plant Sci. 7:193–195. 10.1016/S1360-1385(02)02251-3. 11992820

[kiag413-B45] Kessler SA et al 2010. Conserved molecular components for pollen tube reception and fungal invasion. Science. 330:968–971. 10.1126/science.1195211. 21071669

[kiag413-B46] Kim MC et al 2002. Calmodulin interacts with MLO protein to regulate defence against mildew in barley. Nature. 416:447–451. 10.1038/416447a. 11919636

[kiag413-B47] Koh S, Andre A, Edwards H, Ehrhardt D, Somerville S. 2005. *Arabidopsis thaliana* subcellular responses to compatible *Erysiphe cichoracearum* infections. Plant J. 44:516–529. 10.1111/j.1365-313X.2005.02545.x. 16236160

[kiag413-B48] Kuhn H et al 2017. Key components of different plant defense pathways are dispensable for powdery mildew resistance of the Arabidopsis *mlo2 mlo6 mlo12* triple mutant. Front Plant Sci. 8:1006. 10.3389/fpls.2017.01006. 28674541 PMC5475338

[kiag413-B49] Kusch S et al 2019. Arabidopsis *mlo3* mutant plants exhibit spontaneous callose deposition and signs of early leaf senescence. Plant Mol Biol. 101:21–40. 10.1007/s11103-019-00877-z. 31049793

[kiag413-B50] Kusch S, Panstruga R. 2017. *mlo*-based resistance: an apparently universal “weapon” to defeat powdery mildew disease. Mol Plant Microbe Interact. 30:179–189. 10.1094/MPMI-12-16-0255-CR. 28095124

[kiag413-B51] Kusch S, Pesch L, Panstruga R. 2016. Comprehensive phylogenetic analysis sheds light on the diversity and origin of the MLO family of integral membrane proteins. Genome Biol Evol. 8:878–895. 10.1093/gbe/evw036. 26893454 PMC4824068

[kiag413-B52] Langmead B, Trapnell C, Pop M, Salzberg SL. 2009. Ultrafast and memory-efficient alignment of short DNA sequences to the human genome. Genome Biol. 10:R25. 10.1186/gb-2009-10-3-r25. 19261174 PMC2690996

[kiag413-B53] Li H et al 2009. The sequence alignment/map format and SAMtools. Bioinformatics. 25:2078–2079. 10.1093/bioinformatics/btp352. 19505943 PMC2723002

[kiag413-B54] Li P, Xiao S. 2025. Diverse functions of plant MLO proteins: from mystery to elucidation. Annu Rev Phytopathol. 63:147–173. 10.1146/annurev-phyto-030625-035800. 40903423

[kiag413-B55] Lindner H, Muller LM, Boisson-Dernier A, Grossniklaus U. 2012. CrRLK1L receptor-like kinases: not just another brick in the wall. Curr Opin Plant Biol. 15:659–669. 10.1016/j.pbi.2012.07.003. 22884521

[kiag413-B56] Mayta ML, Hajirezaei M-R, Carrillo N, Lodeyro AF. 2019. Leaf senescence: the chloroplast connection comes of age. Plants (Basel). 8:495. 10.3390/plants8110495. 31718069 PMC6918220

[kiag413-B57] Meng J-G et al 2020. Integration of ovular signals and exocytosis of a Ca^2+^ channel by MLOs in pollen tube guidance. Nat Plants. 6:143–153. 10.1038/s41477-020-0599-1. 32055051

[kiag413-B58] Meyer D, Pajonk S, Micali C, O'Connell R, Schulze-Lefert P. 2009. Extracellular transport and integration of plant secretory proteins into pathogen-induced cell wall compartments. Plant J. 57:986–999. 10.1111/j.1365-313X.2008.03743.x. 19000165

[kiag413-B59] Miklis M et al 2007. Barley MLO modulates actin-dependent and actin-independent antifungal defense pathways at the cell periphery. Plant Physiol. 144:1132–1143. 10.1104/pp.107.098897. 17449647 PMC1914182

[kiag413-B60] Munch DH et al 2026. Distinct membrane trafficking pathways defined by the requirement for GNOM or BIG1 to BIG4 mediate preinvasive immunity toward filamentous fungal pathogens. New Phytol. 250:472–486. 10.1111/nph.70936. 41574444

[kiag413-B61] Nekrasov V et al 2017. Rapid generation of a transgene-free powdery mildew resistant tomato by genome deletion. Sci Rep. 7:482. 10.1038/s41598-017-00578-x. 28352080 PMC5428673

[kiag413-B62] Nelson BK, Cai X, Nebenfuhr A. 2007. A multicolored set of *in vivo* organelle markers for co-localization studies in Arabidopsis and other plants. Plant J. 51:1126–1136. 10.1111/j.1365-313X.2007.03212.x. 17666025

[kiag413-B63] Nishimura MT et al 2003. Loss of a callose synthase results in salicylic acid-dependent disease resistance. Science. 301:969–972. 10.1126/science.1086716. 12920300

[kiag413-B64] Ogawa ST, Zhang W, Staiger CJ, Kessler SA. 2025. MLO-mediated Ca(2+) influx regulates root hair tip growth in Arabidopsis. New Phytol. 248:3024–3039. 10.1111/nph.70378. 40653710 PMC12630461

[kiag413-B65] Panstruga R . 2003. Establishing compatibility between plants and obligate biotrophic pathogens. Curr Opin Plant Biol. 6:320–326. 10.1016/S1369-5266(03)00043-8. 12873525

[kiag413-B66] Panstruga R . 2005. Serpentine plant MLO proteins as entry portals for powdery mildew fungi. Biochem Soc Trans. 33:389–392. 10.1042/BST0330389. 15787613

[kiag413-B67] Pavan S et al 2011. Pea powdery mildew *er1* resistance is associated to loss-of-function mutations at a *MLO* homologous locus. Theor Appl Genet. 123:1425–1431. 10.1007/s00122-011-1677-6. 21850477

[kiag413-B68] Penninckx IA, Thomma BP, Buchala A, Metraux JP, Broekaert WF. 1998. Concomitant activation of jasmonate and ethylene response pathways is required for induction of a plant defensin gene in Arabidopsis. Plant Cell. 10:2103–2113. 10.1105/tpc.10.12.2103. 9836748 PMC143966

[kiag413-B69] Peterhansel C, Freialdenhoven A, Kurth J, Kolsch R, Schulze-Lefert P. 1997. Interaction analyses of genes required for resistance responses to powdery mildew in barley reveal distinct pathways leading to leaf cell death. Plant Cell. 9:1397–1409. 10.2307/3870390. 12237388 PMC157006

[kiag413-B70] Piffanelli P et al 2002. The barley MLO modulator of defense and cell death is responsive to biotic and abiotic stress stimuli. Plant Physiol. 129:1076–1085. 10.1104/pp.010954. 12114562 PMC166502

[kiag413-B71] Piffanelli P et al 2004. A barley cultivation-associated polymorphism conveys resistance to powdery mildew. Nature. 430:887–891. 10.1038/nature02781. 15318221

[kiag413-B72] Qin L et al 2020. Specific recruitment of phosphoinositide species to the plant-pathogen interfacial membrane underlies Arabidopsis susceptibility to fungal infection. Plant Cell. 32:1665–1688. 10.1105/tpc.19.00970. 32156686 PMC7203932

[kiag413-B73] Schulze-Lefert P, Panstruga R. 2003. Establishment of biotrophy by parasitic fungi and reprogramming of host cells for disease resistance. Annu Rev Phytopathol. 41:641–667. 10.1146/annurev.phyto.41.061002.083300. 14527335

[kiag413-B74] Stuttmann J et al 2021. Highly efficient multiplex editing: one-shot generation of 8x *Nicotiana benthamiana* and 12x Arabidopsis mutants. Plant J. 106:8–22. 10.1111/tpj.15197. 33577114

[kiag413-B75] Tang D, Ade J, Frye CA, Innes RW. 2005. Regulation of plant defense responses in Arabidopsis by EDR2, a PH and START domain-containing protein. Plant J. 44:245–257. 10.1111/j.1365-313X.2005.02523.x. 16212604 PMC1797612

[kiag413-B76] Tang D, Ade J, Frye CA, Innes RW. 2006. A mutation in the GTP hydrolysis site of Arabidopsis dynamin-related protein 1E confers enhanced cell death in response to powdery mildew infection. Plant J. 47:75–84. 10.1111/j.1365-313X.2006.02769.x. 16824181 PMC1797611

[kiag413-B77] Tsuda K et al 2013. Dual regulation of gene expression mediated by extended MAPK activation and salicylic acid contributes to robust innate immunity in *Arabidopsis thaliana*. PLoS Genet. 9:e1004015. 10.1371/journal.pgen.1004015. 24348271 PMC3861249

[kiag413-B78] Vogel J, Somerville S. 2000. Isolation and characterization of powdery mildew-resistant *Arabidopsis* mutants. Proc Natl Acad Sci U S A. 97:1897–1902. 10.1073/pnas.030531997. 10677553 PMC26533

[kiag413-B79] von Bongartz K et al 2023. Comprehensive comparative assessment of the *Arabidopsis thaliana* MLO2-CALMODULIN2 interaction by various *in vitro* and *in vivo* protein-protein interaction assays. Biochem J. 480:1615–1638. 10.1042/BCJ20230255. 37767715 PMC10586775

[kiag413-B80] Wan D-Y et al 2020. CRISPR/Cas9-mediated mutagenesis of *VvMLO3* results in enhanced resistance to powdery mildew in grapevine (*Vitis vinifera*). Hortic Res. 7:116. 10.1038/s41438-020-0339-8. 32821399 PMC7395163

[kiag413-B81] Wang W et al 2008. An inositolphosphorylceramide synthase is involved in regulation of plant programmed cell death associated with defense in Arabidopsis. Plant Cell. 20:3163–3179. 10.1105/tpc.108.060053. 19001565 PMC2613663

[kiag413-B82] Wang W, Sijacic P, Xu P, Lian H, Liu Z. 2018. Arabidopsis TSO1 and MYB3R1 form a regulatory module to coordinate cell proliferation with differentiation in shoot and root. Proc Natl Acad Sci U S A. 115:E3045–E3054. 10.1073/pnas.1715903115. 29535223 PMC5879663

[kiag413-B83] Wang W, Wen Y, Berkey R, Xiao S. 2009. Specific targeting of the Arabidopsis resistance protein RPW8.2 to the interfacial membrane encasing the fungal haustorium renders broad-spectrum resistance to powdery mildew. Plant Cell. 21:2898–2913. 10.1105/tpc.109.067587. 19749153 PMC2768920

[kiag413-B84] Wang Y et al 2014. Simultaneous editing of three homoeoalleles in hexaploid bread wheat confers heritable resistance to powdery mildew. Nat Biotechnol. 32:947–951. 10.1038/nbt.2969. 25038773

[kiag413-B85] Wang Z-P et al 2015. Egg cell-specific promoter-controlled CRISPR/Cas9 efficiently generates homozygous mutants for multiple target genes in Arabidopsis in a single generation. Genome Biol. 16:144. 10.1186/s13059-015-0715-0. 26193878 PMC4507317

[kiag413-B86] Wen Y et al 2011. Identification and utilization of a sow thistle powdery mildew as a poorly adapted pathogen to dissect post-invasion non-host resistance mechanisms in Arabidopsis. J Exp Bot. 62:2117–2129. 10.1093/jxb/erq406. 21193574 PMC3060691

[kiag413-B87] Wildermuth MC, Dewdney J, Wu G, Ausubel FM. 2001. Isochorismate synthase is required to synthesize salicylic acid for plant defence. Nature. 414:562–565. 10.1038/35107108. 11734859

[kiag413-B88] Wolter M, Hollricher K, Salamini F, Schulze-Lefert P. 1993. The *mlo* resistance alleles to powdery mildew infection in barley trigger a developmentally controlled defence mimic phenotype. Mol Gen Genet. 239:122–128. 10.1007/BF00281610. 8510641

[kiag413-B89] Wu Y, Sexton W, Yang B, Xiao S. 2023. Genetic approaches to dissect plant nonhost resistance mechanisms. Mol Plant Pathol. 24:272–283. 10.1111/mpp.13290. 36617319 PMC9923397

[kiag413-B90] Xiao S et al 2001. Broad-spectrum mildew resistance in Arabidopsis thaliana mediated by RPW8. Science. 291:118–120. 10.1126/science.291.5501.118. 11141561

[kiag413-B91] Xiao S et al 2005. The atypical resistance gene, RPW8, recruits components of basal defence for powdery mildew resistance in Arabidopsis. Plant J. 42:95–110. 10.1111/j.1365-313X.2005.02356.x. 15773856

[kiag413-B92] Xiao S, Brown S, Patrick E, Brearley C, Turner JG. 2003. Enhanced transcription of the Arabidopsis disease resistance genes *RPW8.1* and *RPW8.2* via a salicylic acid-dependent amplification circuit is required for hypersensitive cell death. Plant Cell. 15:33–45. 10.1105/tpc.006940. 12509520 PMC143449

[kiag413-B93] Yuan J et al 2025. Regulation of MILDEW RESISTANCE LOCUS-O trafficking by calmodulin-binding domains. J Exp Bot. 76:3332–3344. 10.1093/jxb/eraf047. 40036315

[kiag413-B94] Zhang Q et al 2018. Arabidopsis phospholipase Dalpha1 and Ddelta oppositely modulate EDS1- and SA-independent basal resistance against adapted powdery mildew. J Exp Bot. 69:3675–3688. 10.1093/jxb/ery146. 29912376 PMC6022666

[kiag413-B95] Zhang Q et al 2020. Interaction between AtCML9 and AtMLO10 regulates pollen tube development and seed setting. Front Plant Sci. 11:1119. 10.3389/fpls.2020.01119. 32793269 PMC7394235

[kiag413-B96] Zhang Z et al 2008. A lesion-mimic syntaxin double mutant in Arabidopsis reveals novel complexity of pathogen defense signaling. Mol Plant. 1:510–527. 10.1093/mp/ssn011. 19825557

[kiag413-B97] Zheng Z et al 2013. Loss of function in *Mlo* orthologs reduces susceptibility of pepper and tomato to powdery mildew disease caused by *Leveillula taurica*. PLoS One. 8:e70723. 10.1371/journal.pone.0070723. 23923019 PMC3726601

[kiag413-B98] Zhu L, Zhang X-Q, Ye D, Chen L-Q. 2021. The mildew resistance locus O 4 interacts with CaM/CML and is involved in root gravity response. Int J Mol Sci. 22:5962. 10.3390/ijms22115962. 34073116 PMC8198571

