# Supplementary material for: The role of MLO in powdery mildew susceptibility depends on a combination of functional specialization and subcellular localization
Source: Plant Physiol. 2026 Jun 24;201(3):kiag413. doi: 10.1093/plphys/kiag413 (PMC13360292; doi:10.1093/plphys/kiag413)
Supplement: kiag413_Supplementary_Data [file kiag413_supplementary_data.pdf]

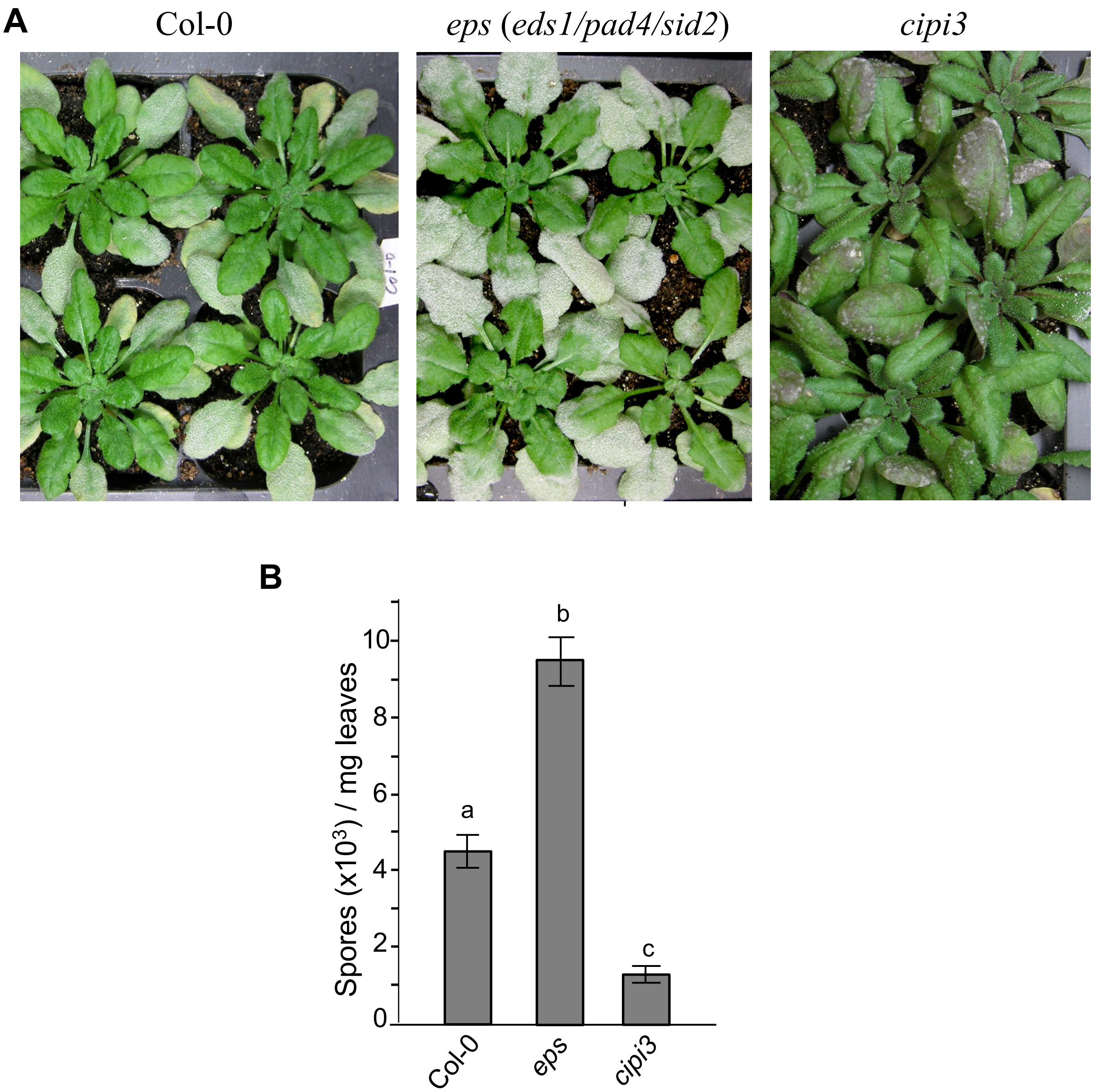


**Supplementary Figure S1.** *cipi3* mutant plants exhibit strong resistance to *Golovinomyces cichoracearum* (*Gc*) UCSC1.

**A**, Greatly reduced susceptibility of *cipi3* to *Gc* UCSC1 compared with wild-type Col-0 and the *eds1/pad4/sid2* (*eps*) parental line. Eight-week-old plants of the indicated genotypes were inoculated with *Gc* UCSC1. Photos were taken at 10 dpi. Note the trichome-based infection in *cipi3*.
**B**, Quantification of disease susceptibility of the three genotypes to *Gc* UCSC1 as measured by the total number of spores per mg infected leaves at 12 dpi. Data represent mean ± standard error (n=4). Different letters indicate statistically significant differences ($P$<0.001) between the three genotypes, as determined by multiple comparisons using one-way ANOVA, followed by Tukey's HSD test. This experiment was repeated once with similar results.

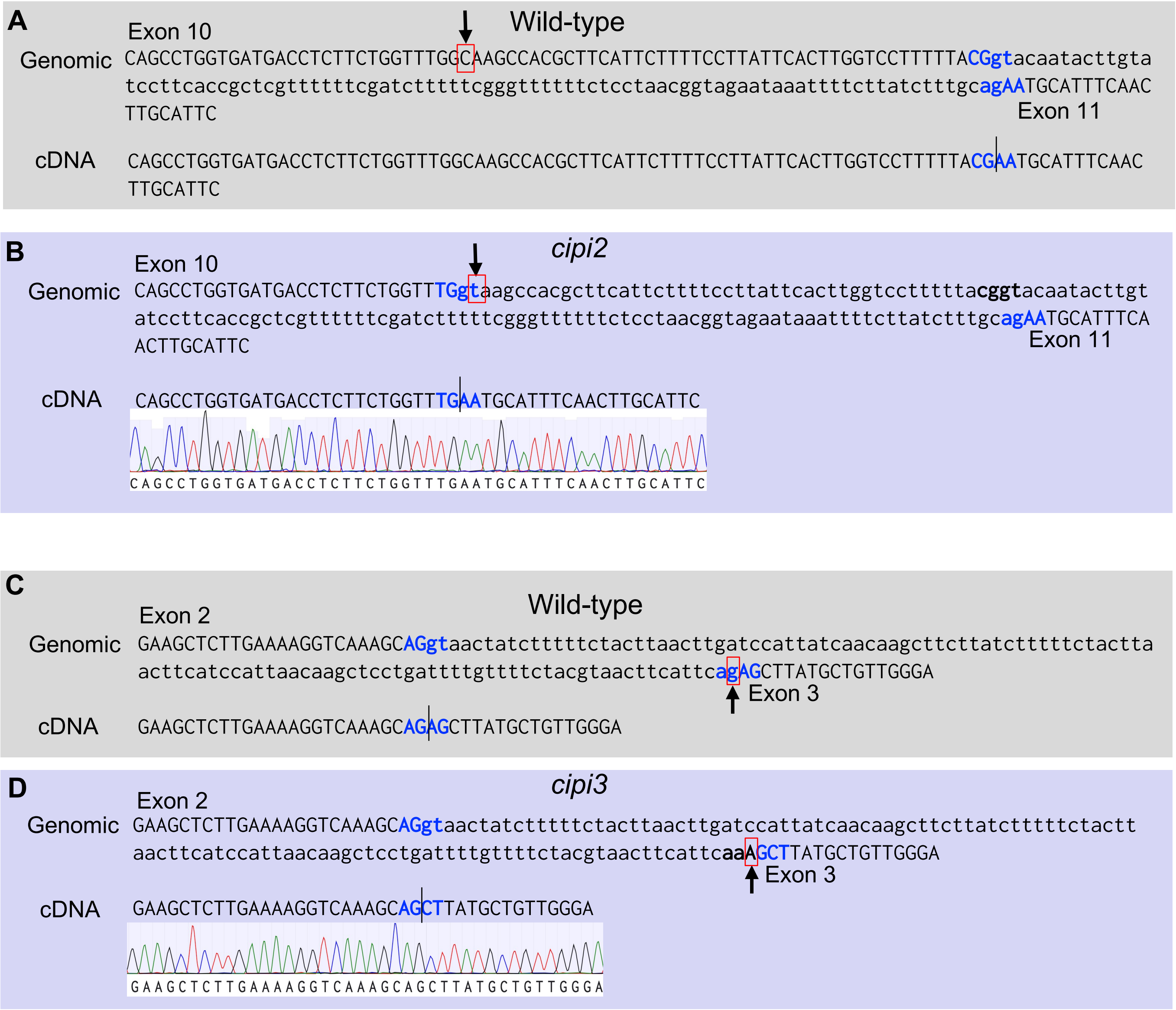


**Supplementary Figure S2.** Predicted and sequence-confirmed mis-splicing of *MLO2* mRNA in the *cipi2* and *cipi3* mutants.

Shown are the genomic DNA and the corresponding complementary DNA (cDNA) coding sequences of *MLO2* in the regions that contain the *cipi2* (**A**, **B**) or the *cipi3* (**C**, **D**) mutation (red boxed). The nucleotides of the introns are in lower case. The splicing motifs are highlighted in blue. The vertical bar indicates the splice boundary after removal of the intron in the cDNA. Arrows indicate the C-to-T mutation in *cipi2* (**B**) or G-to-A the mutation in *cipi3* (**D**) confirmed by the respective Sanger sequencing chromatographs.

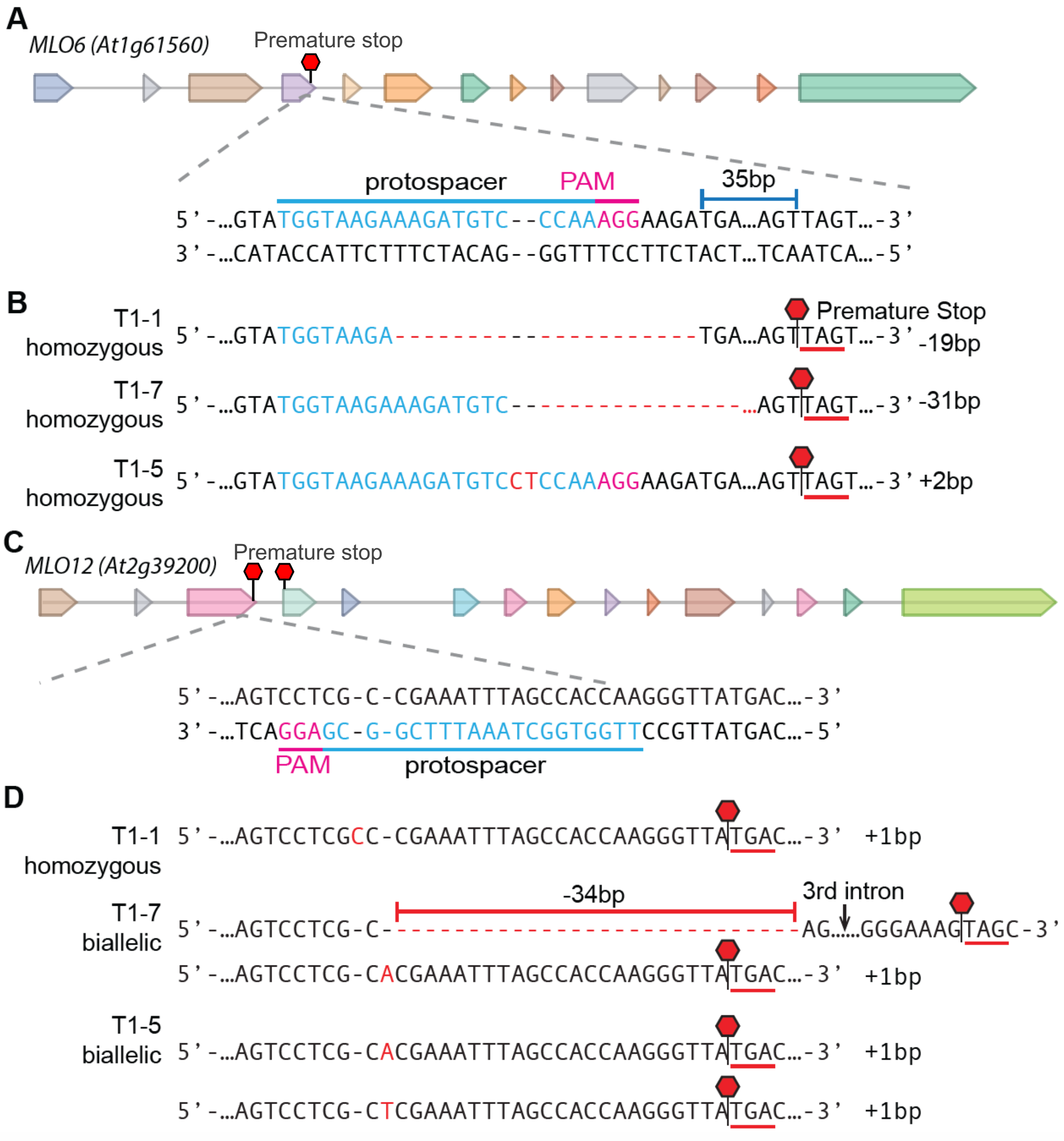


**Supplementary Figure S3.** CRISPR/Cas9-targeted mutagenesis of *MLO6* and *MLO12* in *cipi3*.

**A**, Gene structure of *MLO6* with the protospacer and PAM sequence marked.
**B**, Three independent lines with indels in *MLO6* and the position of the premature stop codon marked with red hexagons.
**C**, Gene structure of *MLO12* with the protospacer and PAM sequence marked.
**D**, Three independent lines with indels in *MLO12* and the position of premature stop codon marked with red hexagons.

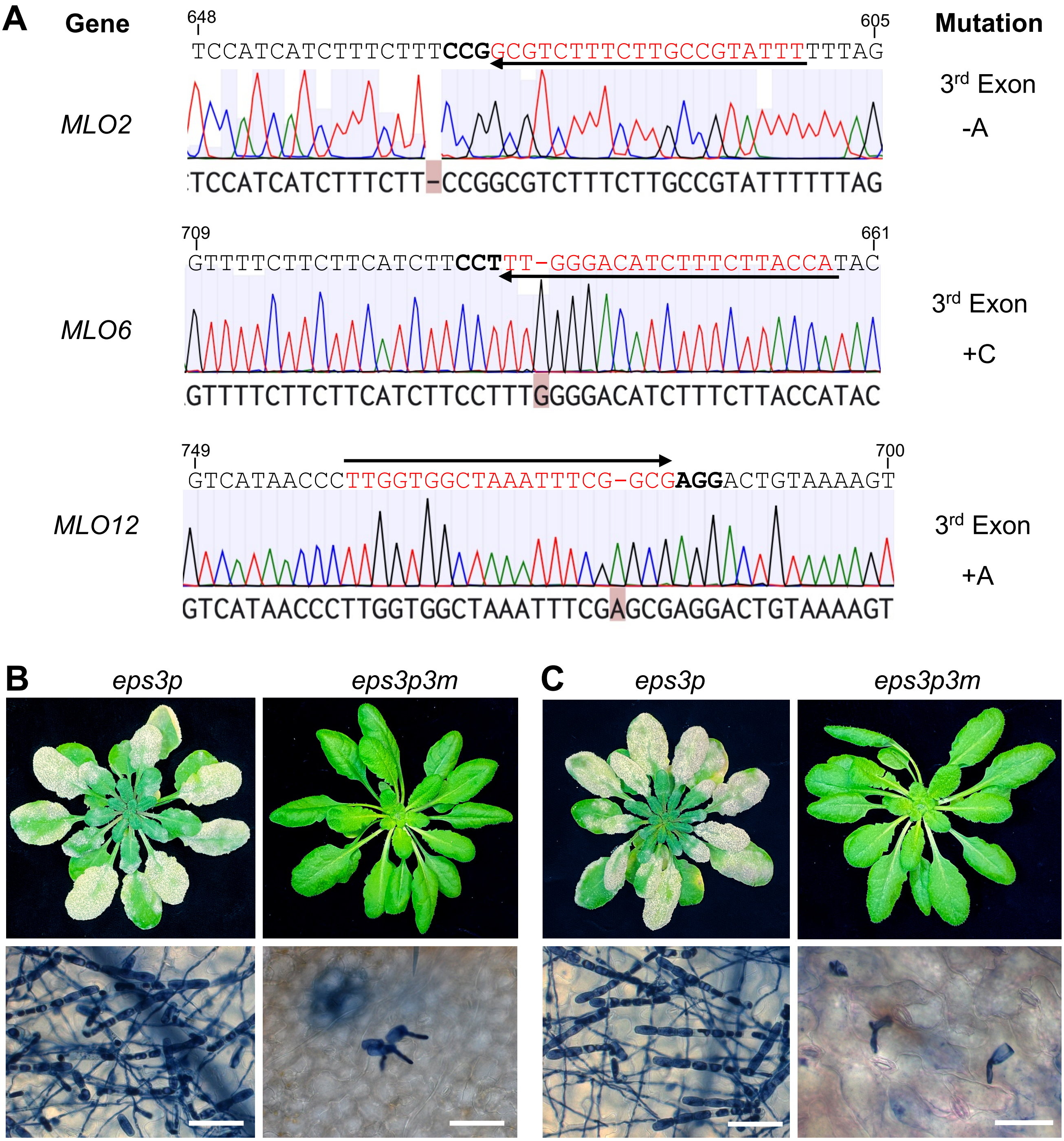


**Supplementary Figure S4.** Knocking out *MLO2*, *MLO6* and *MLO12* in a higher-order immuno-compromised mutant background also results in failure of powdery mildew pathogenesis.

**A**, CRISRP/Cas9-mutagenesis was employed to knock out *MLO2*, *MLO6* and *MLO12* in the *esd1/pad4/sid2/ pen1/pen2/pen3* (*eps3p*) background to generate the nonuple mutant *eds1/pad4/sid2/pen1/pen2/pen3/mlo2/mlo6/mlo12* (*eps3p3m*). Shown are chromatograms of the targeted regions of the three genes in line #2. Arrowed lines indicate protospacers; Bold-faced are PAM motifs.

**B**,**C**, Plants of *eps3p* and *eps3p3m* were inoculated with the adapted PM isolate *Gc* UCSC1 (**B**) or a non-adapted isolate *Gc* UMSG1 (**C**). Photos of infected plants were taken at 11 dpi. Micrographs were representative sections of infected leaves stained with trypan blue. Bar = 50 μm.

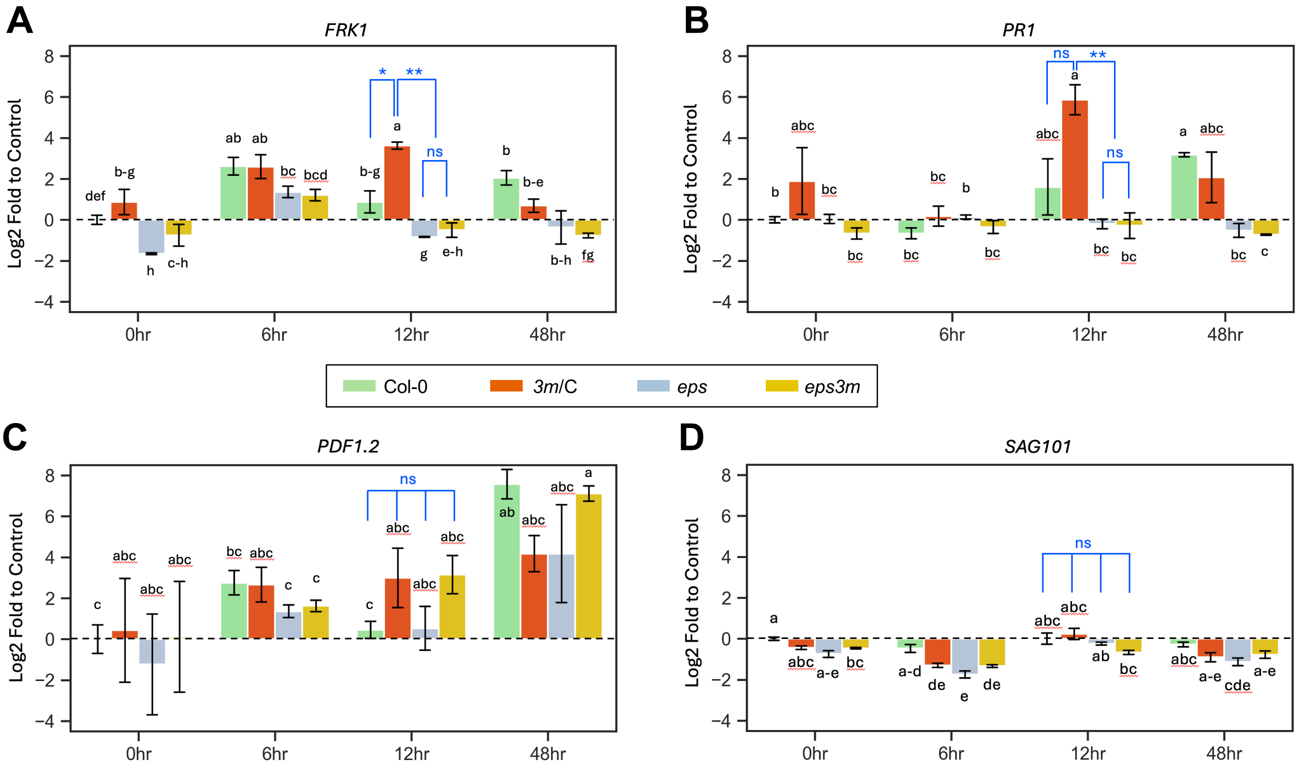


**Supplementary Figure S5.** No activation of PTI and ETI marker genes in *eps3m.*

Six-week-old, short-day grown plants of the four indicated genotypes were inoculated with *Gc* UCSC1. Inoculated leaves were collected at the indicated four timepoints after inoculation and subjected to RT-qPCR analysis to measure expression of four indicated marker genes. The control group is defined as Col-0, 0hr. Data represent mean ± standard deviation of three replicates. All gene groups pass ANOVA with $p<0.05$. Post-hoc analyses (multiple comparisons) are conducted through *T*-test adjusted by Benjamini-Hochberg FDR procedure. *$p<0.05$, **$p<0.01$, ***$p<0.001$". ns, no significant difference.

**A**, Expression of *FRK1*, reporting activation of PTI.
**B**, Expression of *PR1*, reporting activation of salicylic acid-dependent defenses, mostly ETI.
**C**, Expression of *PDF1.2*, reporting activation of the Jasmonic acid- and ethylene-dependent defenses.
**D**, Expression of *SAG101*, reporting the onset of leaf senescence.

This experiment was repeated twice with similar results.

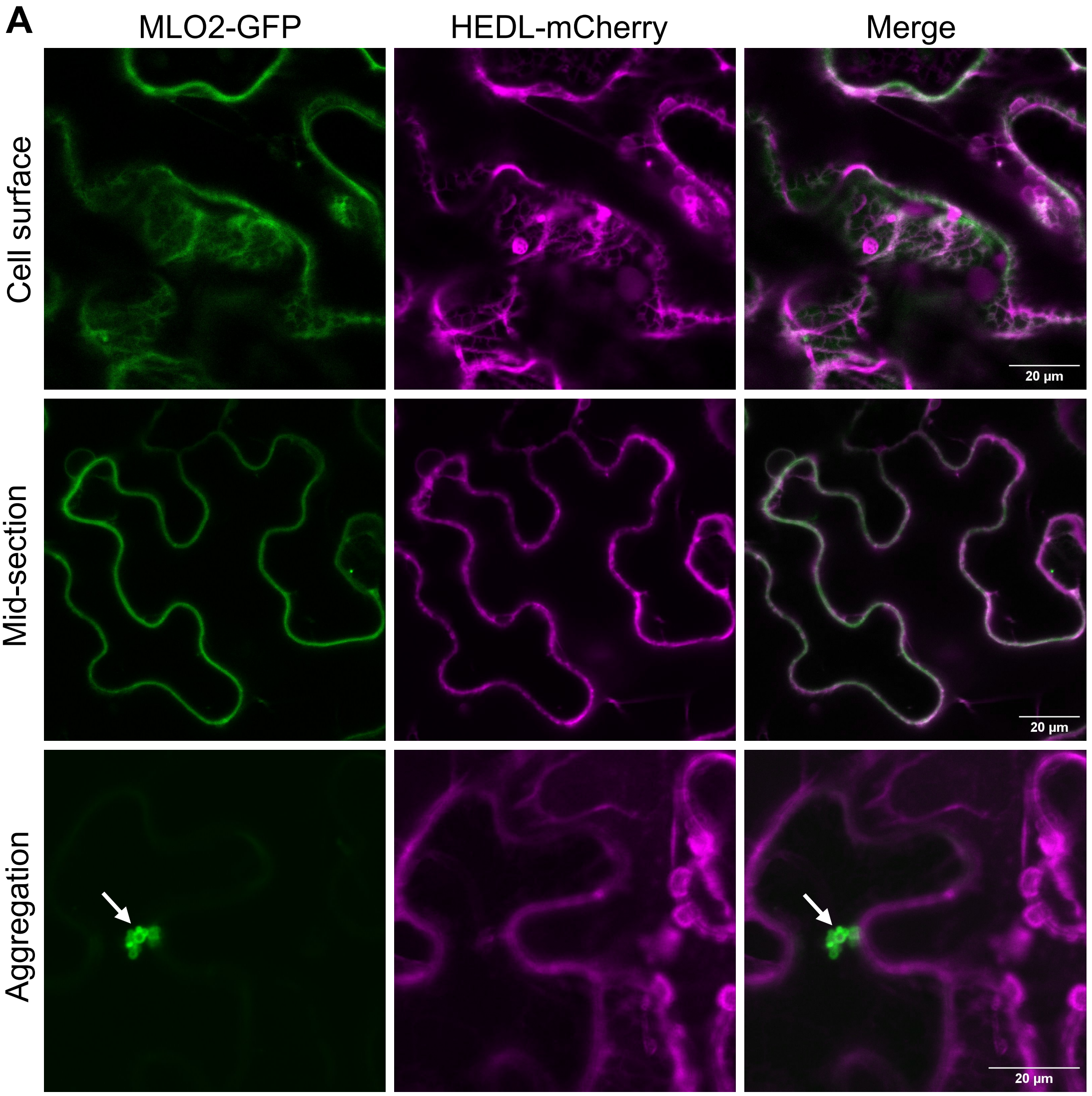


**Supplementary Figure S6.** MLO2-GFP exhibits partial ER- and partial Golgi-localization in leaf epidermal cells of *N. benthamiana.*

Agrobacterium cells harboring *pMLO2::MLO2-GFP* were mixed in equal concentration ($OD_{600}$=0.5) with those harboring the ER marker *35S::HEDL-mCherry* (**A**) or the Golgi marker *35S::Man1-mCherry* (**B**). The mixtures were infiltrated into leaves of *N. benthamiana*. Confocal images were acquired at 2 days after agroinfiltration. Shown are Z-stack projections of 3-5 optical sections. Note when MLO2-GFP was detected as “grape string”-like aggregations (indicated by white arrow) shown in the lower panel, there was little if any co-localization with the ER marker, but good colocalization with the Golgi marker.

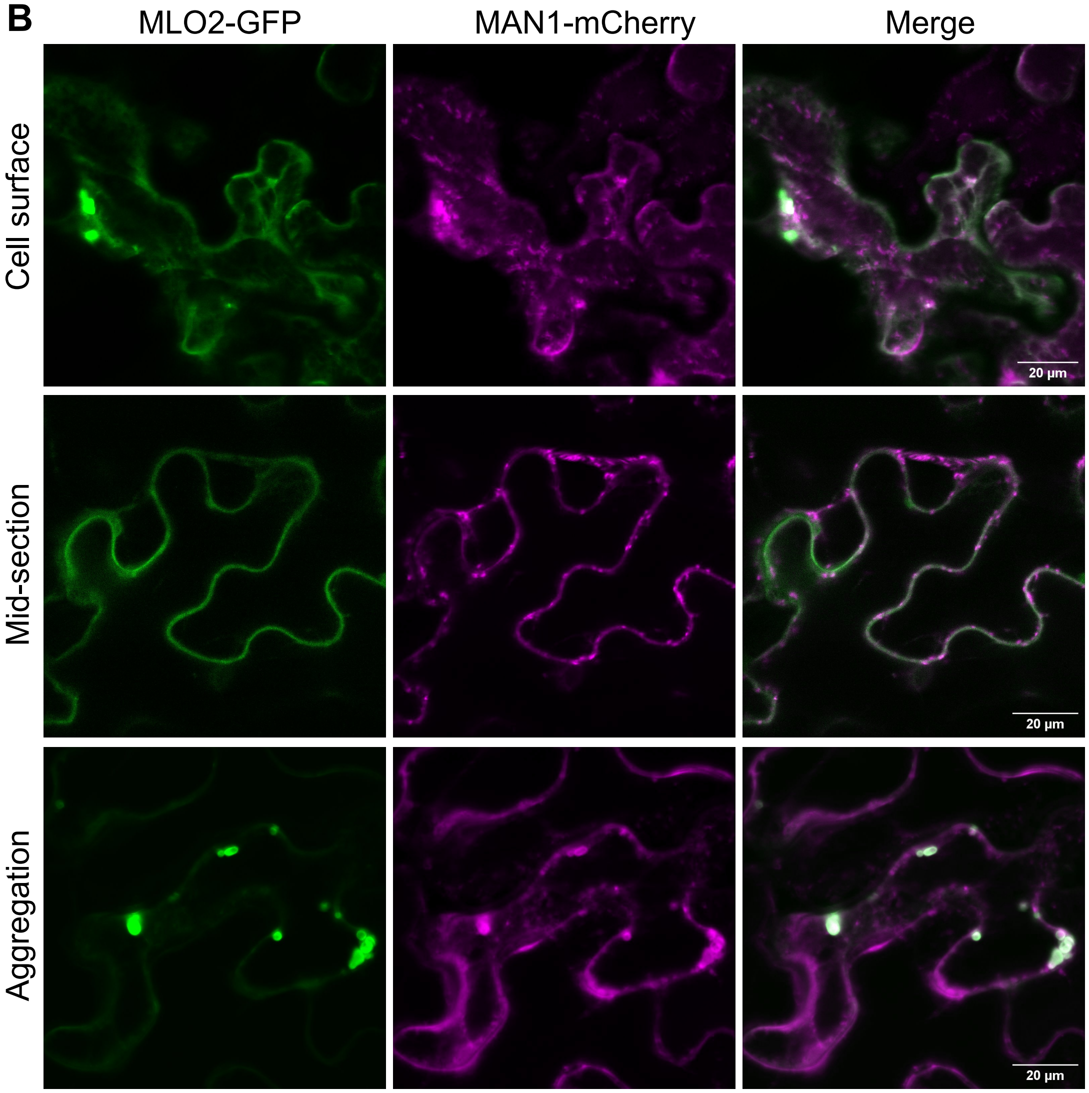


**Supplementary Figure S6 (continued).** MLO2-GFP exhibits partial ER- and partial Golgi-localization in leaf epidermal cells of *N. benthamiana.*

Agrobacterium cells harboring *pMLO2::MLO2-GFP* were mixed in equal concentration ($OD_{600}$=0.5) with those harboring the ER marker *35S::HEDL-mCherry* (**A**) or the Golgi marker *35S::Man1-mCherry* (**B**). The mixtures were infiltrated into leaves of *N. benthamiana*. Confocal images were acquired at 2 days after agroinfiltration. Shown are Z-stack projections of 3-5 optical sections. Note when MLO2-GFP was detected as “grape string”-like aggregations (indicated by white arrow) shown in the lower panel, there was little if any co-localization with the ER marker, but good colocalization with the Golgi marker.

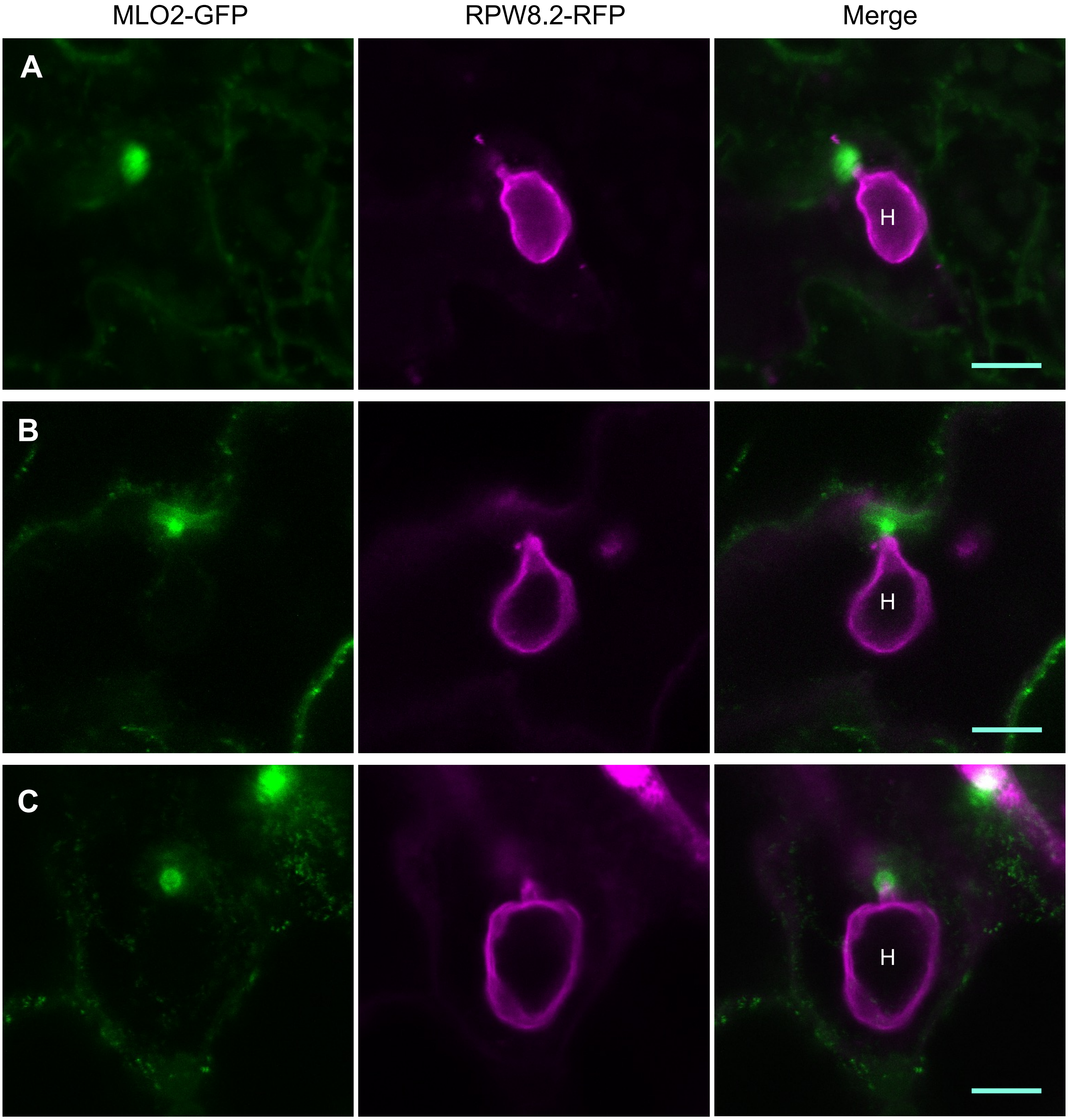


**Supplementary Figure S7.** MLO2-GFP is localized to the peri-penetration peg membranous space (PPM) next to the haustorial neck.

Plants of *eps3m* expressing MLO2-GFP and RPW8.2-RFP were inoculated with *Gc* UCSC1. Infected leaves were subjected to confocal microscopy at 2-3 dpi.
**(A-C)** Confocal z-stack (3–5) projections showing individual GFP and RFP channels and the merged image. Note, the merged images are also shown in **Figure 6F**. H, haustorium. Bar = 10 µm.

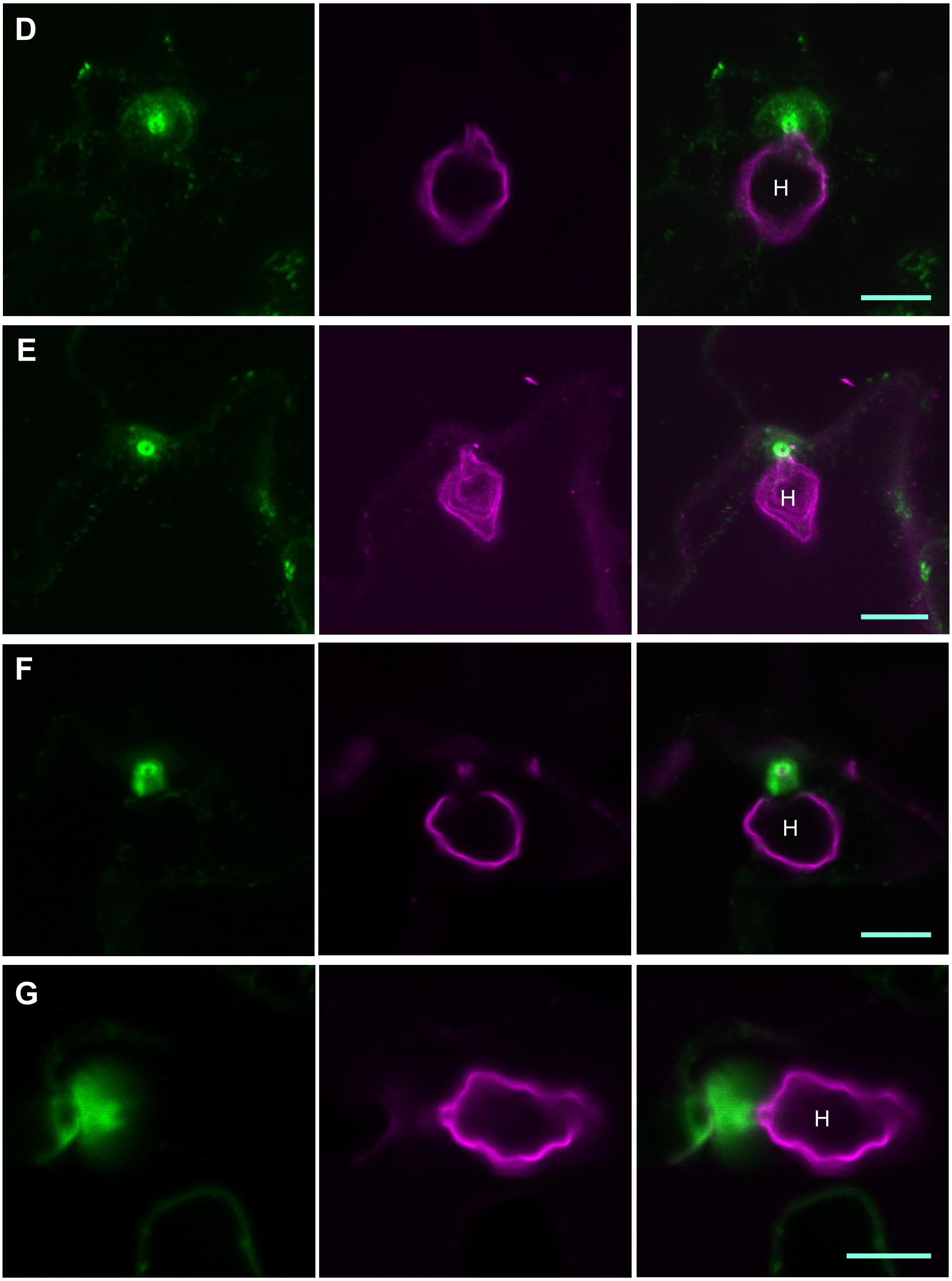


**Supplementary Figure S7 (Continued).** MLO2-GFP is localized to the peri-penetration peg membranous space (PPM) next to the haustorial neck.

Plants of *eps3m* expressing MLO2-GFP and RPW8.2-RFP were inoculated with *Gc* UCSC1. Infected leaves were subjected to confocal microscopy at 2-3 dpi.
**(D-G)** Additional confocal z-stack (3–5) projections showing individual GFP and RFP channels and the merged image. H, haustorium. Bar = 10 µm.

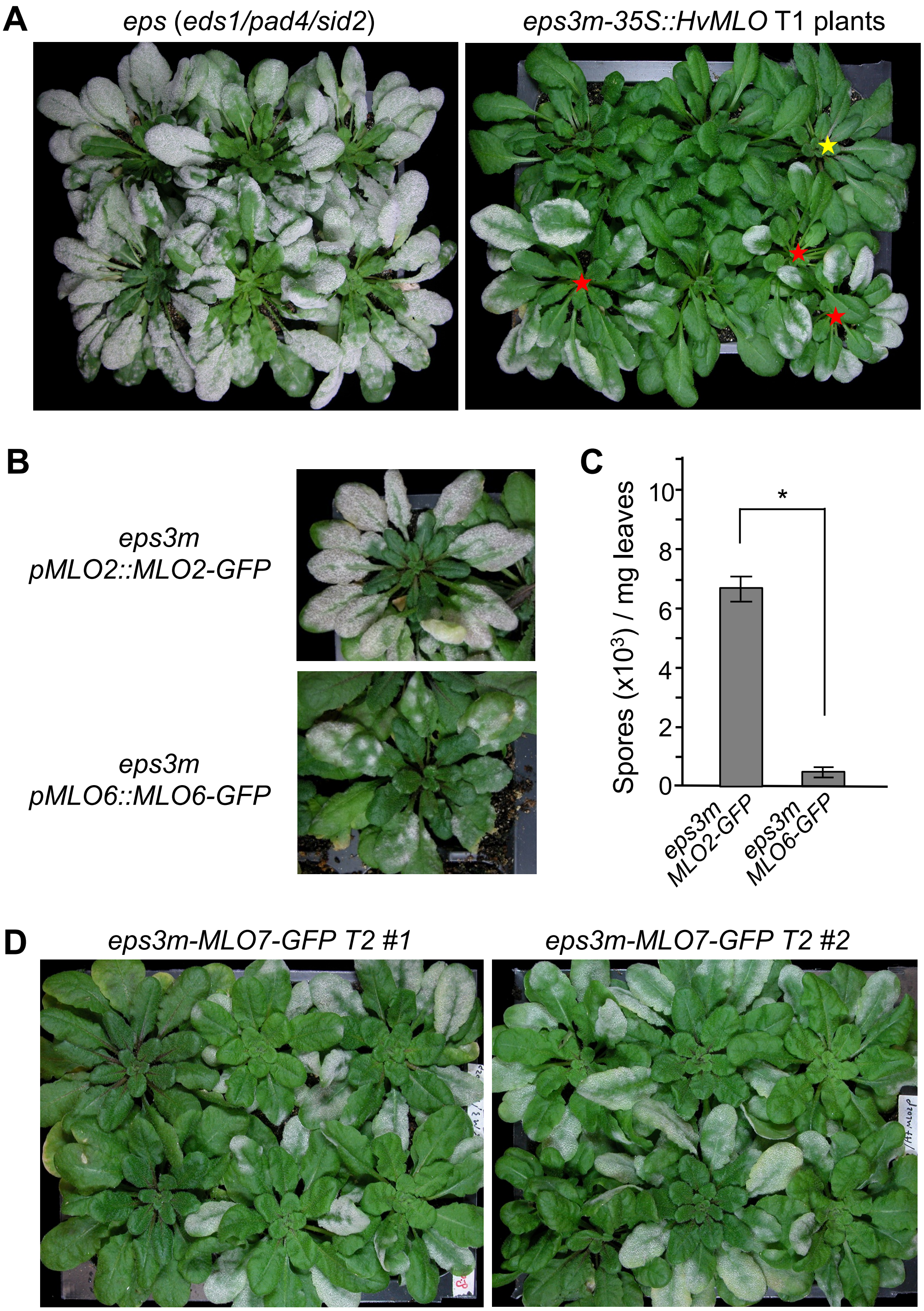


**Supplementary Figure S8.** Expression of HvMLO1-GFP, MLO6-GFP or MLO7-GFP in *eps3m* partially restored susceptibility to *Gc* UCSC1.

**A**, Representative T1 plants of *eps3m* transgenic for *35S::HvMLO1* infected with *Gc* UCSC1 at 12 dpi. Note, among nine T1 plants shown, five were resistant as *eps3m*, three were moderately susceptible (red stars) while one was weakly susceptible (yellow star) compared to *eps* plants.
**B**,**C**, Representative photos of the indicated transgenic lines infected with *Gc* UCSC1 at 11 dpi (B) and their levels of susceptibility (C). Data represent mean ± standard error (n=4). Asterisk indicates significant difference ($p$<0.001; unpaired Student's $t$-test).
**D**, Infection phenotypes of the T2 progenies of two *eps3m* lines transgenic for *pMLO2::MLO7-GFP*. Photos were taken at 11 dpi with *Gc* UCSC1.

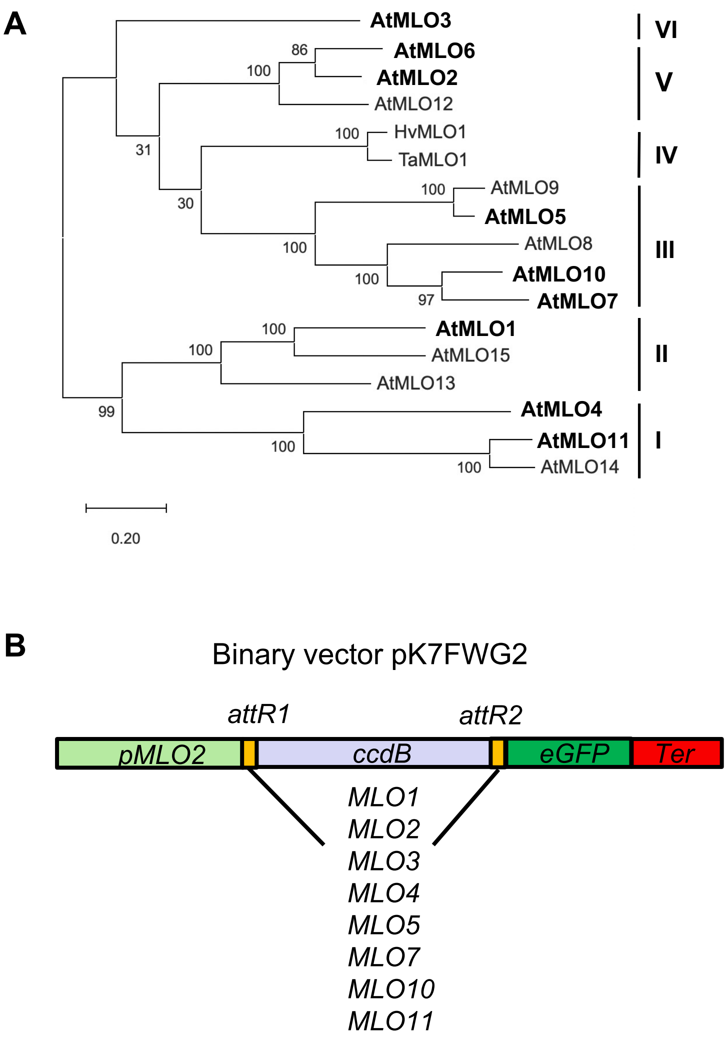


**Supplementary Figure S9.** Selection and cloning of seven *MLO* family members from five different clades for ectopic expression in leaves of *eps3m* from the *MLO2* promoter.

**A**, A phylogenetic tree of the Arabidopsis MLO family plus barley and wheat clade IV MLO1 constructed based on deduced amino acid sequences using MEGA12 [Kumar S., Stecher G., Suleski M., Sanderford M., Sharma S., and Tamura K. (2024). *Molecular Evolutionary Genetics Analysis Version 12 for adaptive and green computing*. Molecular Biology and Evolution 41:1-9]. Bold-faced are *MLO* family members (belonging to different clades) that were subjected to expression and localization analyses.
**B**, Schematic showing the binary vector for expressing the eight indicated *MLO* genes from the *MLO2* promoter. Note, *ccdB* is a lethal gene that targets bacterial DNA gyrase; attR1 and attR2 are specific bacteriophage (lambda) DNA sequences used in the Gateway Cloning system to facilitate directional, high-efficiency gene cloning. Ter, terminator.

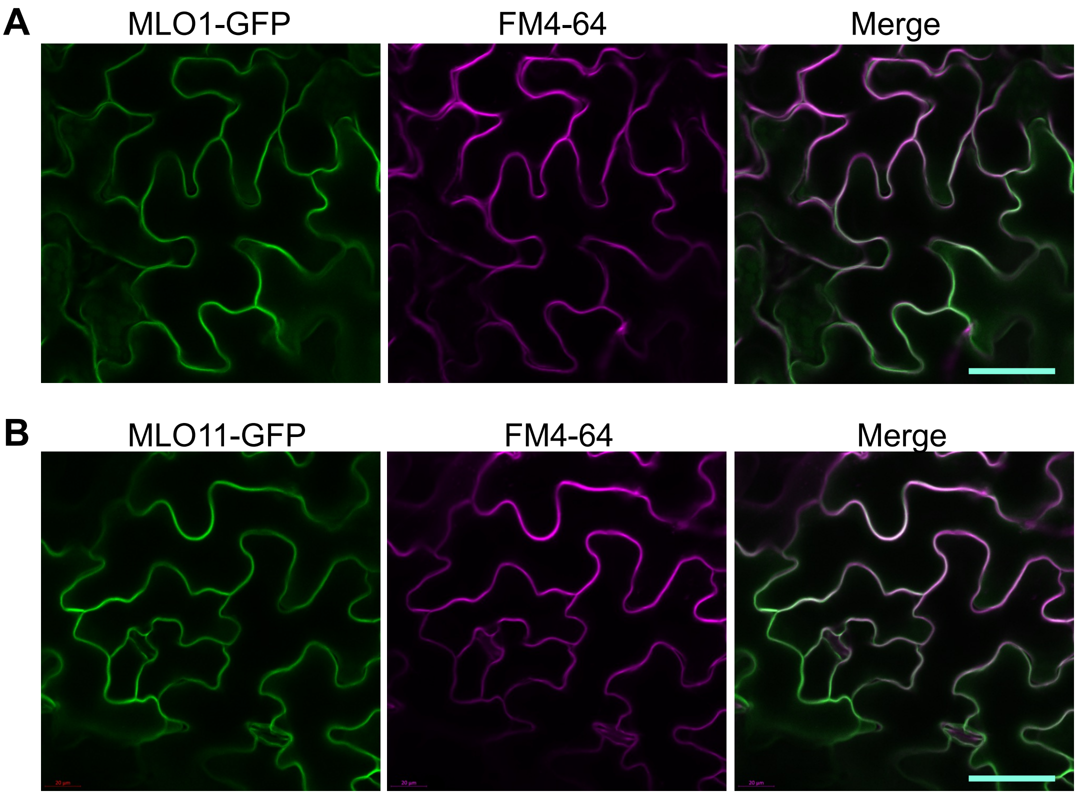


**Supplementary Figure S10.** MLO1-GFP and MLO11-GFP are localized to the plasma membrane.

Leaves of *eps3m* transgenic plants expressing MLO1-GFP (**A**) or MLO11-GFP (**B**) from the *MLO2* promoter were stained with 20 μM FM4-64 for 15 min before confocal imaging. Shown are representative z-stack confocal images projected from 3-5 thin optical sections. Bar = 50 μm.

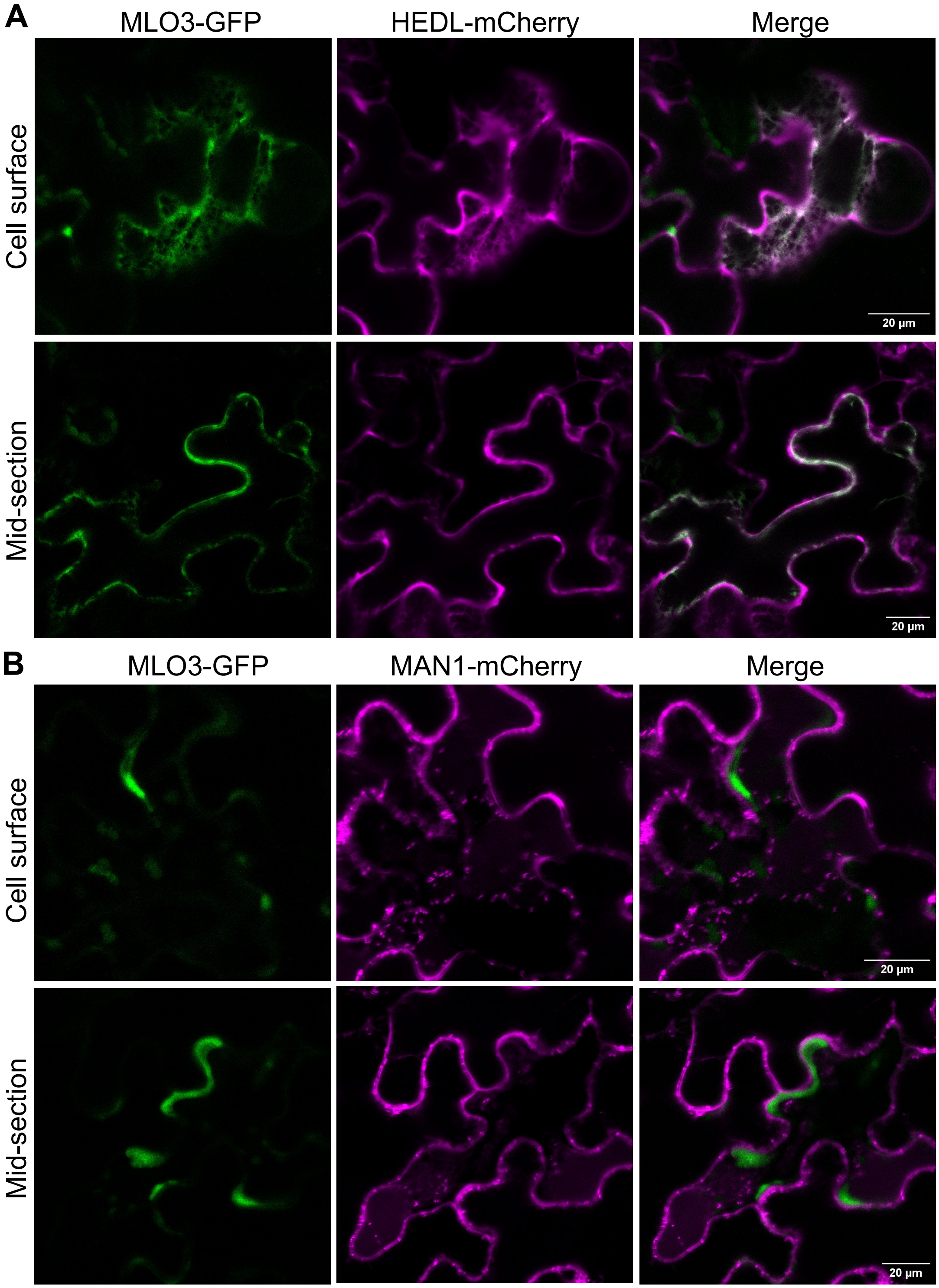


**Supplementary Figure S11.** MLO3-GFP exhibits ER-localization in leaf epidermal cells of *N. benthamiana.*

Agrobacterium cells harboring *pMLO2::MLO3-GFP* were mixed in equal concentration ($OD_{600}$=0.5) with those harboring the ER marker *35S::HEDL-mCherry* (**A**) or the Golgi marker *35S::Man1-mCherry* (**B**). The mixtures were infiltrated into leaves of *N. benthamiana*. Confocal images were acquired at 2 days after agroinfiltration. Shown are Z-stack projections of 3-5 optical sections.

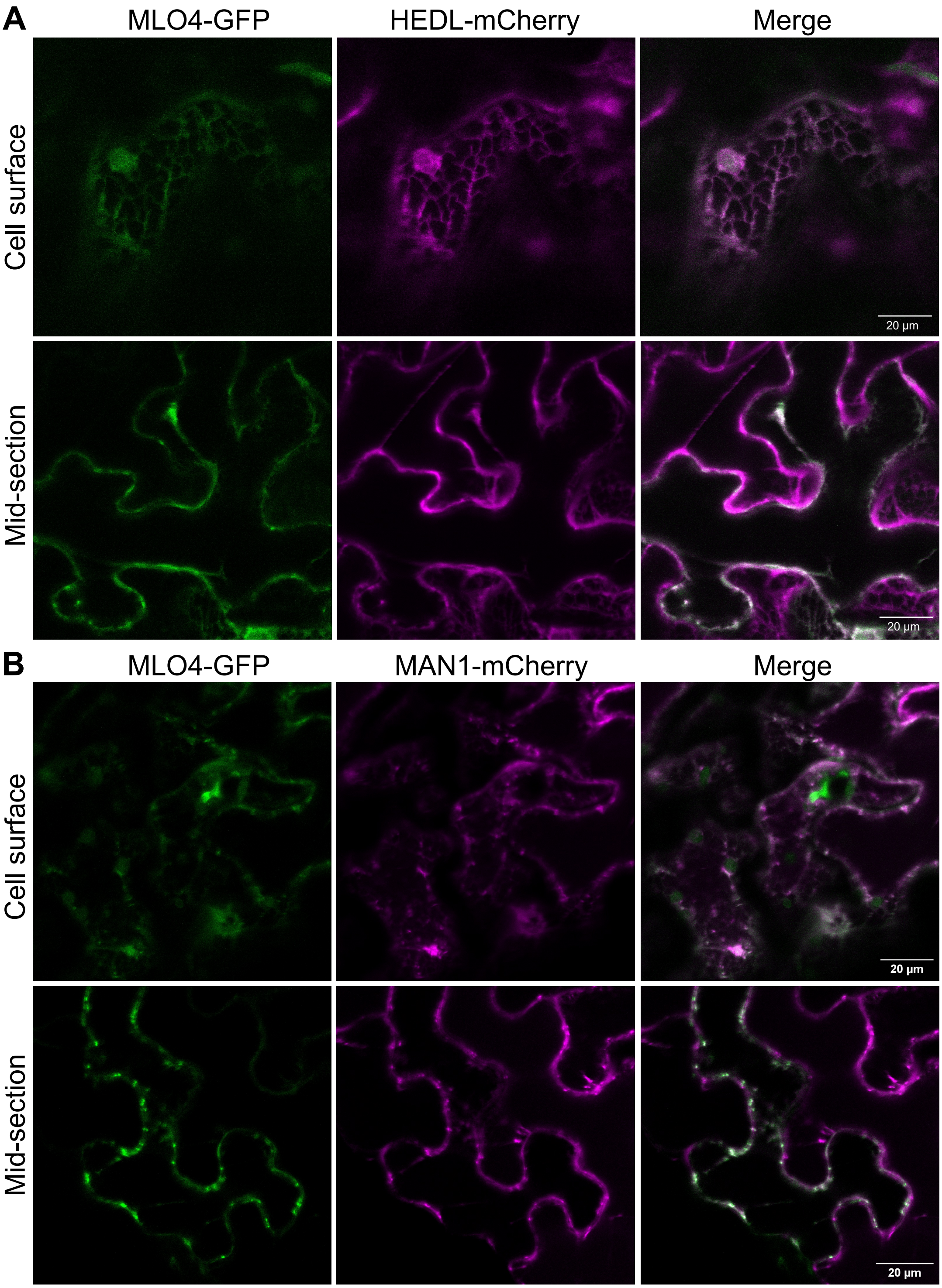


**Supplementary Figure S12.** MLO4-GFP exhibits partial ER- and partial Golgi-localization in leaf epidermal cells of *N. benthamiana.*

Agrobacterium cells harboring *pMLO2::MLO4-GFP* were mixed in equal concentration ($OD_{600}$=0.5) with those harboring the ER marker *35S::HEDL-mCherry* (**A**) or the Golgi marker *35S::Man1-mCherry* (**B**). The mixtures were infiltrated into leaves of *N. benthamiana*. Confocal images were acquired at 2 days after agroinfiltration. Shown are Z-stack projections of 3-5 optical sections.

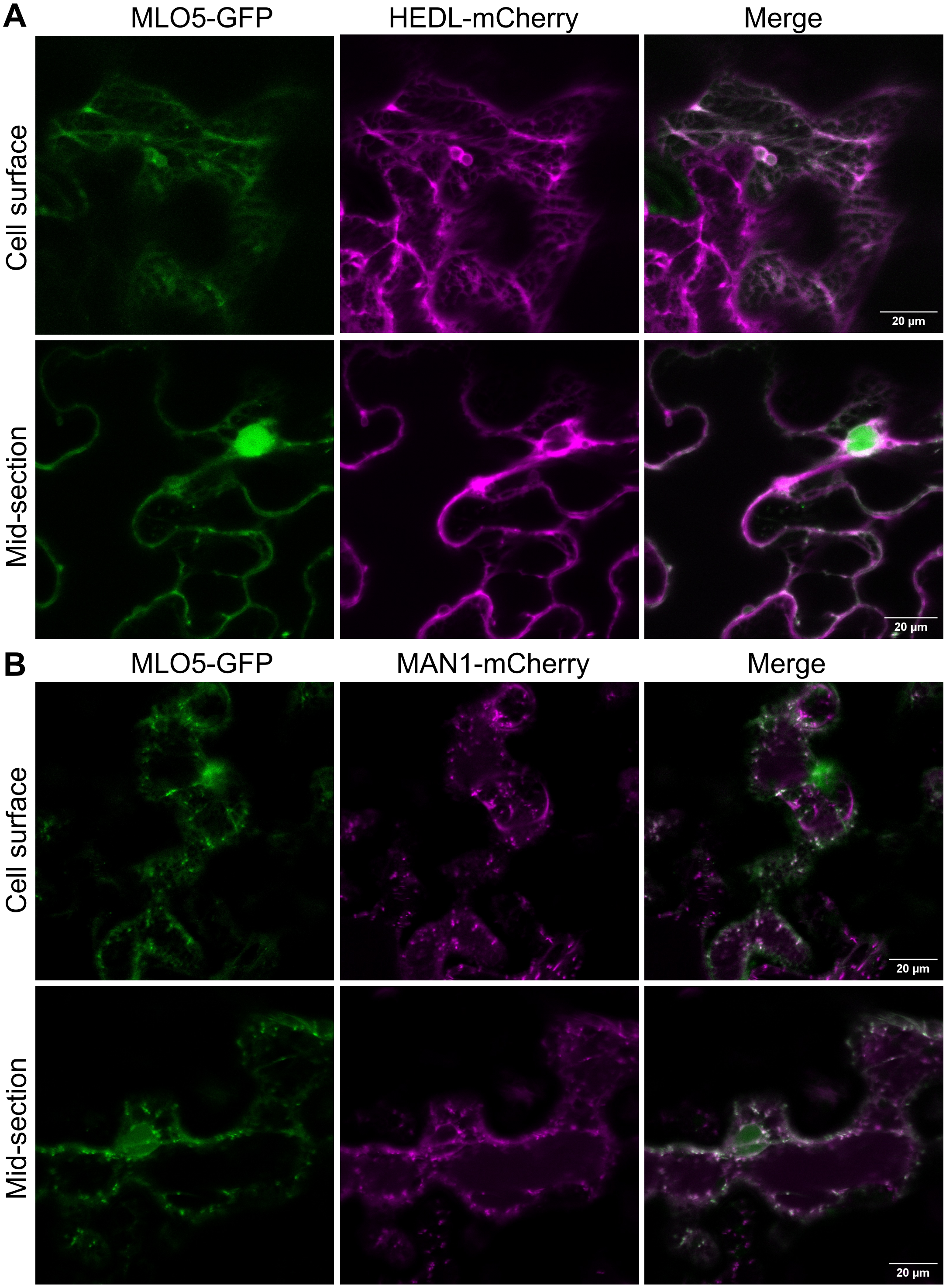


**Supplementary Figure S13.** MLO5-GFP exhibits partial ER- and partial Golgi-localization in leaf epidermal cells of *N. benthamiana.*

Agrobacterium cells harboring *pMLO2::MLO5-GFP* were mixed in equal concentration ($OD_{600}$=0.5) with those harboring the ER marker *35S::HEDL-mCherry* (**A**) or the Golgi marker *35S::Man1-mCherry* (**B**). The mixtures were infiltrated into leaves of *N. benthamiana*. Confocal images were acquired at 2 days after agroinfiltration. Shown are Z-stack projections of 3-5 optical sections.

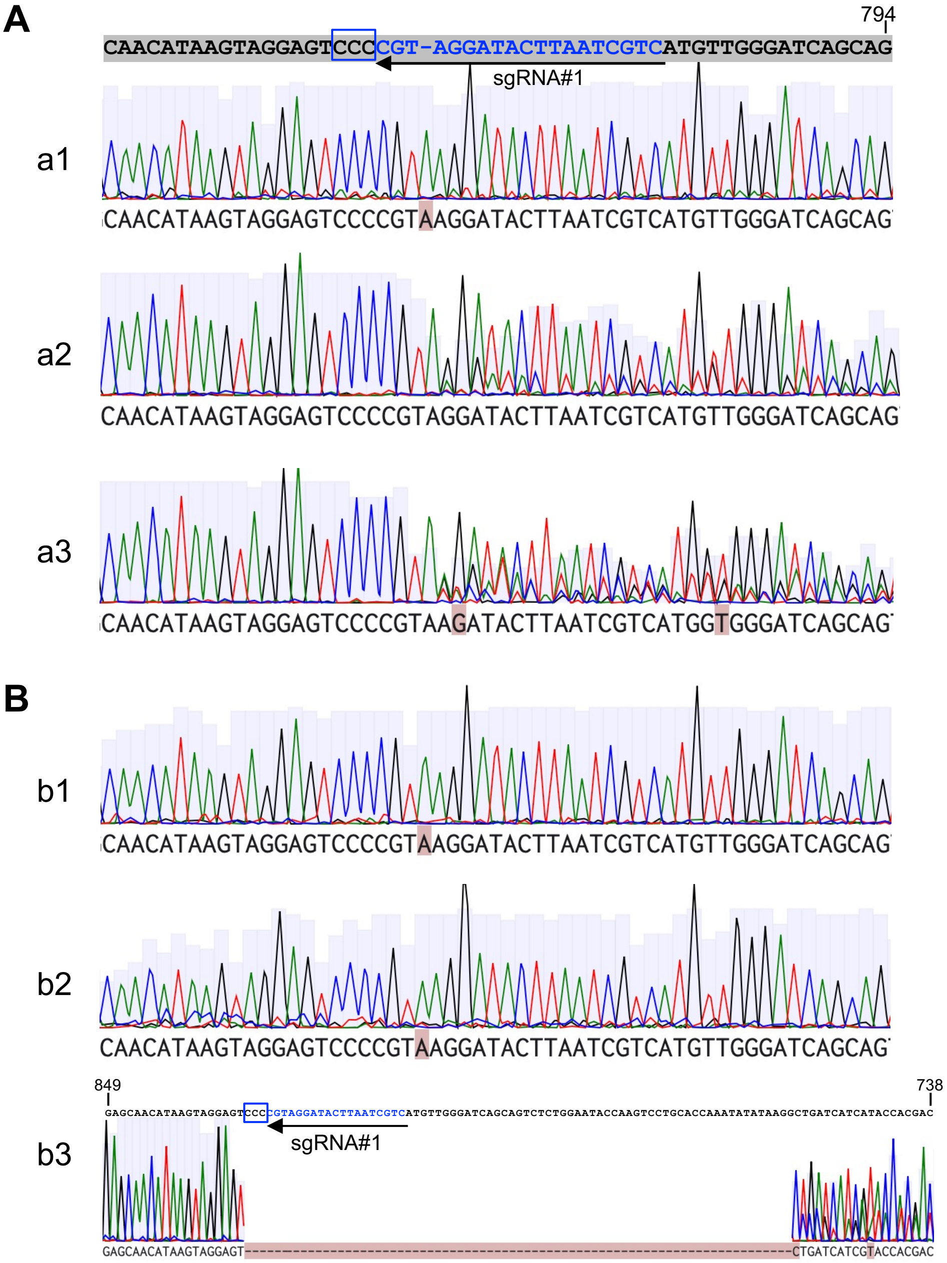


**Supplementary Figure S14.** CRISPR/Cas9-targeted mutagenesis of *FER.*

The CRISPR construct was introduced into the background of *eds1/pad4/sid2* (*eps*) or *eds1/pad4/sid2/mlo2/mlo6/mlo12* (*eps3m*) *eps3m* expressing MLO2-GFP and RPW8.2-RFP.

**A**, Sanger sequencing chromatograms of the sgRNA target regions in three independent T1 lines with a compact rosette in the *eps* triple mutant background.
**B**, Sanger sequencing chromatograms of the sgRNA target regions in three independent T1 lines with a compact rosette in the background of *eps3m* transgenic for *pMLO2::MLO2-GFP* and *pRPW8.2::RPW8.2-RFP.*

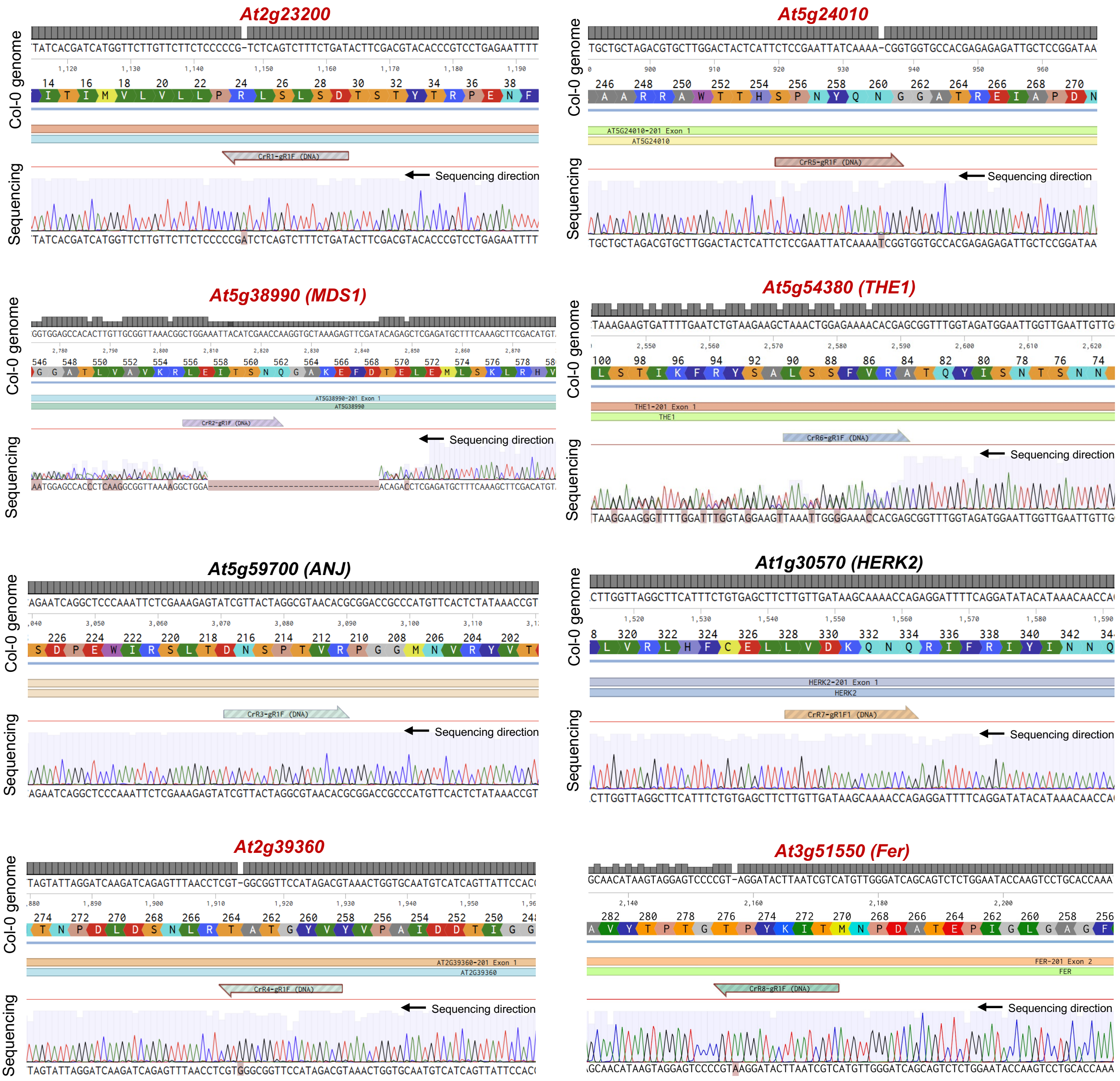


**Supplementary Figure S15.** Multiplexed CRISPR targeting eight *CrRLK1L* family members.

The multiplexed CRISPR construct was introduced into *eps3m* expressing MLO2-GFP and RPW8.2-RFP. Shown are the sequence alignments between the wild-type (Col-0) sequence and the Sanger sequencing chromatograms of the sgRNA-targeting regions of eight *CrRLK1L* genes in one mutant line (e2) exhibiting compact rosette and susceptibility phenotypes. Notably, six (highlighted in red) of the eight targeted *CrRLK1L* genes contain disruptive indels.

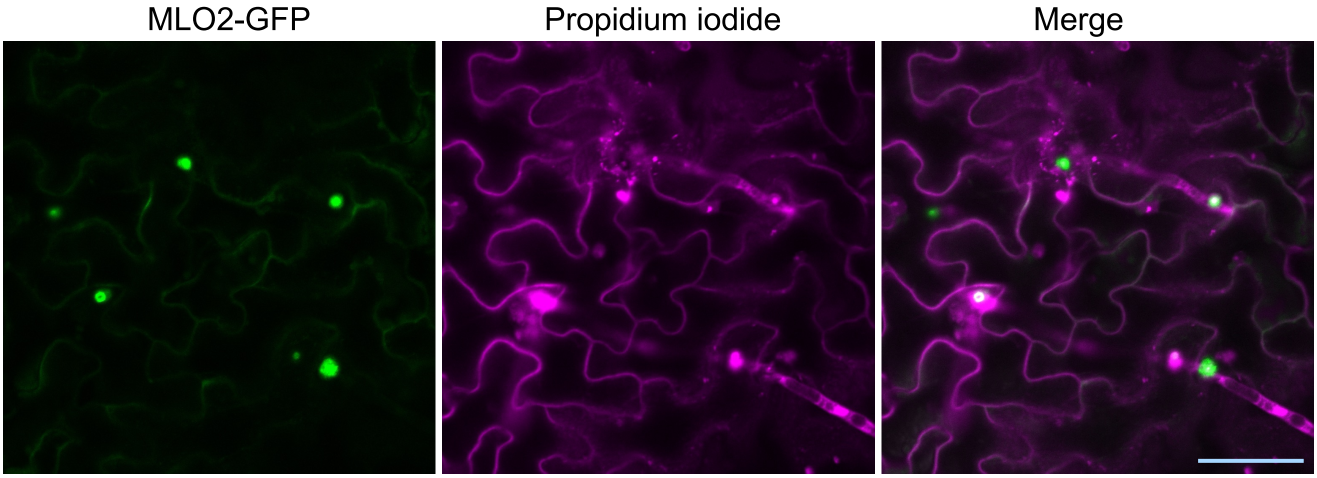


**Supplementary Figure S16.** Six CrRLK1L family members including FER are dispensable for MLO2-GFP's focal accumulation.

A representative confocal image showing normal focal accumulation of MLO2-GFP at the fungal penetration site in PM-infected leaves of plants of *eps3m/pMLO2-MLO2-GFP/pRPW8.2-RPW8.2-RFP* line e2 that is transgenic for the CRISPR construct targeting eight *CrRLK1L* family genes. Note, six *CrRLK1L* family genes including *FER* contain disruptive indels in this line. Bar = 50 μm.

**Supplementary Table S1**. Primers for cloning *MLO* genes.

| Gene name | Gene ID | Primer name | Sequence (5' to 3') |
|---|---|---|---|
| *MLO1* | AT4G02600 | TP-MLO1F | CACCATGGGTCACGGAGGAGAAG |
| | | MLO1R | GTTGTTATGATCAGGTGTAATCTCATTGTT |
| *MLO2* | AT1G11310 | TP-MLO2F | TTTCTTAAAAGaaaaaTCTCTTAAGCTTTT |
| | | MLO2-nsR | CTCTTTTACTTGATCTGCCATGGTG |
| *MLO3* | AT3G45290 | TP-MLO3F | CACCATGACGGATAAAGAAGAAAGCAACCA |
| | | MLO3R | CCTTTCAGTTTTCTCTTGTATCTGTATCT |
| *MLO4* | AT1G11000 | TP-MLO4F | CACCATGGAGCATATGATGAAAGAAGGAAG |
| | | MLO4R | AGTCCTCCTAAACAACTCAAGAAGT |
| *MLO5* | AT2G33670 | TP-MLO5F | caccaTGGCTGGAGGAGGAGGT |
| | | MLO5-nsR | GGGACCGCTTAAGAGGTCT |
| *MLO6* | AT1G61560 | TP-MLO6pF | CACCGGACATTTGTGCCAATAGGAAA |
| | | MLO6-nsR | TCGCTTAAACGAAAAATCCCTTAAGCT |
| *MLO7* | AT2G17430 | TP-MLO7F | cacCATGATCACAAGAAGCAGGTGT |
| | | MLO7-nsR | TGTGGAATTGCATCTCCTGTTGT |
| *MLO10* | AT5G65970 | TP-MLO10F | CACCATGGCCACAAGATGCTTTTGG |
| | | MLO10-nsR | GTCAATATCATTAGCAGGAACGTTCTT |
| *MLO11* | AT5G53760 | TP-MLO11F | caccATGGGAGAAGGAGAAGAAAATGGA |
| | | MLO11-nsR | GACTCTCTTCTCACTTGGCAAG |
| *MLO2p* | AT1G11310 | Xba-Mlo2p-F | GCGATCTAGACTCTTTGAACTTAAGTTGTGTCCAA |
| | | BamH-Mlo2p-R | TGCGGATCCTAAGAAAAAACCCCAAATAAGAT |
| *MLO2n-1c* | not available | Mlo2-Ct1-F | AATGAAAATTGTGTGCAGATGGGAAGTAGCTTCAAGAAA |
| | | Mlo2-Ct1-R | TTTCTTGAAGCTACTTCCCATCTGCACACAATTTTCATT |
| *MLO1n-2c* | not available | Mlo1-Ct2-F | ACCCTTTTCTTATATTACAACAGATGGGTAGTAAAATGAAGCC |
| | | Mlo1-Ct2-R | GGCTTCATTTTACTACCCATCTGTTGTAATATAAGAAAAGGGT |

**Supplementary Table S2**. Primers for RT-qPCR.

| Gene name | Gene ID | Primer Name | Sequence (5' to 3') |
|---|---|---|---|
| *FRK1* | AT2G19190 | FRK1-e76R | TTCAACGTTTAATTCGGTCAAGTT |
| | | FRK1-e5F | AGAGGCCAGATAGATCCAGC |
| *PDF1.2* | AT5G44420 | PDF1.2-e12F | TTGCTGCTTTCGACGCAC |
| | | PDF1.2-e2R | GCTCCTTCAAGGTTAATGCACT |
| *SAG101* | AT5G14930 | SAG101-e23F | CCTCAAATCAGAGTTGCTCCAA |
| | | SAG101-e3R | GTCGGTTCGATGGTTTCAAGA |
| *PR1* | AT2G14610 | AtPR1-F | AGAGGCAACTGCAGACTCATACAC |
| | | AtPR1-R | AGCCTTCTCGCTAACCCACAT |
| *UBC9* | AT4G27960 | AtUBC9-F | CAGTGGAGTCCTGCTCTCACAA |
| | | AtUBC9-R | CATCTGGGTTTGGATCCGTTA |

**Supplementary Table S3**. Primers for cloning small guide RNAs.

| Gene name | Gene ID | Primer name | Sequence (5' to 3') |
|---|---|---|---|
| *MLO2* | AT1G11310 | Mlo2-gRNA1F | ATATATGGTCTCGATTGAAATACGGCAAGAAAGACGCGTTTTAGAGCTAGAAATAGC |
| | | Mlo2-gRNA1R | ATTATTGGTCTCGAAACGCTTGTGCTTCTTTTTAAACCAATCTCTTAGTCGACTCTAC |
| *MLO6* | AT1G61560 | Mlo6-gRNA1F | ATATATGGTCTCGATTGTGGTAAGAAAGATGTCCCAAGTTTTAGAGCTAGAAATAGC |
| *MLO12* | At2g39200 | Mlo12-gRNA2R | ATTATTGGTCTCGAAACCGCCGAAATTTAGCCACCAACAATCTCTTAGTCGACTCTAC |
| not available | At2g23200 | CrR1-gR1F | attgTATCAGAAAGACTGAGACGG |
| | | CrR1-gR1R | aaacCCGTCTCAGTCTTTCTGATA |
| *MDS1* | At5g38990 | CrR2-gR1F | attgCTGGAAATTACATCGAACCA |
| | | CrR2-gR1R | aaacTGGTTCGATGTAATTTCCAG |
| *ANJ* | At5g59700 | CrR3-gR1F | attgTCGTTACTAGGCGTAACACG |
| | | CrR3-gR1R | aaacCGTGTTACGCCTAGTAACGA |
| *CYV1* | At2g39360 | CrR4-gR1F | attgACGTCTATGGAACCGCCACG |
| | | CrR4-gR1R | aaacCGTGGCGGTTCCATAGACGT |
| not available | At5g24010 | CrR5-gR1F | attgTCTCCGAATTATCAAAACGG |
| | | CrR5-gR1R | aaacCCGTTTTGATAATTCGGAGA |
| *THE1* | At5g54380 | CrR6-gR1F | attgTAAACTGGAGAAAACACGAG |
| | | CrR6-gR1R | aaacCTCGTGTTTTCTCCAGTTTA |
| *HERK2* | At1g30570 | CrR7-gR1F | attgTTGTTGATAAGCAAAACCAG |
| | | CrR7-gR1R | aaacCTGGTTTTGCTTATCAACAA |
| *FER* | At3g51550 | CrR8-gR1F | attgTGACGATTAAGTATCCTACG |
| | | CrR8-gR1R | aaacCGTAGGATACTTAATCGTCA |
| | | CrR8-gR2F | attgTGTTGGACCCATAGACCTCG |
| | | CrR8-gR2R | aaacCGAGGTCTATGGGTCCAACA |

**Supplementary Table S4.** Primers for genotyping or sequencing (continued).

| Gene name | Gene ID | Primer name | Sequence (5' to 3') |
|---|---|---|---|
| *MLO1* | AT4G02600 | MLO1-e5R | GAGTGACCCTTCTTTTCCTGAGA |
| *MLO2 prom* | AT4G02600 | AtMlo2p-F2 | GACAAGATCTCTGGTCTGGAATTAGA |
| *MLO2* | AT1G11310 | MLO2-e1F | TCAAAAGAAGAACACGAAACTCTG |
| | | MLO2e2F | GAAGCACAAGCAGGCTCTTTTT |
| | | MLO2-e3R | GGGTTTATCTCCATCTCCATCATCTT |
| | | MLO2-e6F | GGGACACATCTTTTGGGAGA |
| | | MLO2-e8R | GCACAGCGACAAACCAGATA |
| | | MLO2-e9F | ACCTCTGGTTACCATTCATTCC |
| | | MLO2-e11R | TCCAGGCAAAGAATGCAAGT |
| | | cipi3-testmRNA-R | TGGTGGCTAAACTTCTTCTATGGA |
| | | cipi2_testmRNA-F | CATTTTGCTCCCGGTAACGA |

**Supplementary Table S4.** Primers for genotyping or sequencing (continued).

| Gene name | Gene ID | Primer name | Sequence (5' to 3') |
|---|---|---|---|
| *MLO6* | AT1G61560 | MLO6-e4F | CCTTGTAGTGCATCCGAAGAA |
| | | MLO6-e4R | TGACAAACCGCAAGAACAAA |
| *MLO12* | AT2G39200 | MLO12-e3F | CCAGTCTCCGAGATTTGCATT |
| | | MLO12-e4R | TGCAGCTGGTGGATACCATA |
| *MLO3* | AT3G45290 | MLO3-e2R | TAGAAAAACGTCGCTACTTGAAGC |
| *MLO4* | AT1G11000 | MLO4-e3R | GCGCTTTGTGACAACAGAAGT |
| *MLO5* | AT2G33670 | MLO5-e3R | CTTCTTGCACTGAGCTGGAG |
| *MLO7* | AT2G17430 | MLO7-e2R | GGAGAGAGTTTTTATGCTTCTTCCAT |
| *MLO10* | AT5G65970 | MLO10-e3R | TCTCCGAAGGTGAGCAACAA |
| *MLO11* | AT5G53760 | MLO11-e6R | GCTACATCTTCCCATATCCTCCA |
| not available | AT2G23200 | CrRLK1-tp-F | caccATGGAGAATTTCTGTTTTCAAGACTCT |
| | | CrRLK1-e1R | GCGAGTTCATCATCAGGAGAAA |
| | | CrRLK1_gR1seq | TATTTCAGGCGCAACGGACGA |
| *MDS1* | AT5G38990 | CrRLK2_gR1PCR-F | TCTTCCATCAGATCTCTGCCG |
| | | CrRLK2_gR1PCR-R2 | TGATCTGATCAACGGTTCTTTTATTGA |
| | | CrRLK2_gR1seq | TGGAGGATCAGAGGCCTTGT |
| *ANJ* | AT5G59700 | CrRLK3-e2F | CCAGTAATCGCAATTCAAATTCAGATATTT |
| | | CrRLK3-e2R | GCAAAACCAGGAACATAATCAACAGA |
| | | CrRLK3_gR1seq | CGGATCATCTGGAGCTCACGT |
| *CVY1* | AT2G39360 | CrRLK4-tpF | caccATGATAAACCTCAAGCTATTCTTAGAGCT |
| | | CrRLK4-e1R | AGTGAAACCGAACAAAGTAATCGA |
| | | CrRLK4_gR1seq | CCTGAAGATGCAAAACTCATTGGT |
| not available | AT5G24010 | CrRLK5-e1F | CGAAATTGACGCCGTTTAATGATA |
| | | CrRLK5-e1R | CCTCATGATCTCAACTCCATTCAA |
| | | CrRLK5_gR1seq | AGAGCTGATTAAGCGACGAACT |
| *THE1* | AT5G54380 | CrRLK6-tpF | caccATGGTGTTCACAAAATCATTACTTGTTCT |
| | | CrRLK6-e1R | GTTACGACAGTGATTGAAGCAGA |
| | | CrRLK6_gR1seq | GCTTTGTTTAATCCGCCAGACA |
| *HERK2* | AT1G30570 | CrRLK7_gR1PCR-F | GGTGGAAGTGAAGTGGAGCT |
| | | CrRLK7-e1R | GATGAGTCAGGTCCAAGTTGAATC |
| | | CrRLK7_gR1seq | GAGAACGCTGGTGTAGAAGTCA |
| *FER* | At3g51550 | CrRLK8-e2R | CTAACAAGGTAAGTGAAACCAGAGT |
| | | CrRLK8-e2F | CTCCGTCTCCTTTGGTCCTT |
